# Proteomic Examination for Gluconeogenesis Pathway-Shift during Polyhydroxyalkanoate Formation in *Cupriavidus necator* Grown on Glycerol

**DOI:** 10.3390/bioengineering7040154

**Published:** 2020-12-01

**Authors:** Nuttapol Tanadchangsaeng, Sittiruk Roytrakul

**Affiliations:** 1 College of Biomedical Engineering, Rangsit University, 52/347 Phahonyothin Road, Lak-Hok, Pathumthani 12000, Thailand; 2 Proteomics Research Laboratory, National Center for Genetic Engineering and Biotechnology (BIOTEC), 113 Thailand Science Park, Khlong Luang, Pathumthani 12120, Thailand; sittiruk@biotec.or.th

**Keywords:** proteomics, gluconeogenesis, glycerol, polyhydroxyalkanoate, protein regulations, mass spectrometry, *Cupriavidus necator*

## Abstract

Because of availability and inexpensiveness, glycerol can be considered as a suitable raw material for polyhydroxyalkanoate (PHA) production with bacterial fermentation. Nevertheless, compared to the production of glucose as a raw precursor, PHA produced from glycerol by *Cupriavidus necator* was found to produce lower PHA with low bacterial growth rates. According to our study, *C. necator* was able to synthesize glucose-like intermediates from glycerol via gluconeogenesis. This resulted in a decrease of the cell dry weight and the yield of PHA polymers, especially in the active cell growth phase. It was indicated that glycerol used as a carbon source of the PHA synthesis pathway has glucogenesis-shift, which causes a decrease of the PHA content and productivity. In this research, we investigated the proteins that were closely expressed with the increase of the intracellular PHA and glucose content. For solving the above problem, the proteins inside the bacterial cells were analyzed and compared to the database proteins via mass spectrometry. The proteins were isolated by 1-D SDS-polyacrylamide gel electrophoresis (PAGE) technique and identified by the liquid chromatography mass spectrometry (LC-MS) technique. By using bioinformatics validation, a total number of 1361 proteins were examined and found in the culture bacterial cells. Selective protein expression was correlated with the amount of PHA at each cultivation time and generating glucose by studying the 1361 proteins was elucidated in proteomic information. The results of the cluster of proteins were found to contain 93 proteins using the multiple array viewer (MEV) program with the KMS data analysis model. Protein species with the same expression pattern for PHA and six proteins with similar expression patterns were found to be correlated with generating glucose content. The associations of the two protein groups were then determined through a Stitch program. The protein and chemical associations were analyzed both directly and indirectly through different databases. The proteins of interest were found with research data linked between glycerol and glucose. Five protein types are connecting to glucose and glycerol shift pathway, two of which are glycosyl hydrolase (H16_B1563) and short-chain dehydrogenase (H16_B0687), both of which are enzymes used to break the bonds of complex sugars, possibly related to the partial conversion of glycerol to glucose. The two proteins found in the strains used in the *Cupriavidus necator* H16 experiment give rise to the break down the bonds of α,α-1,1-glucoside of malto-oligosyltrehalose and short-chain sugar molecules such as mannitol (C_6_H_14_O_6_), respectively. In this research, finding the associated expression proteins which is involved in changing the pathway of gluconeogenesis shift to PHA synthesis will be useful information on genetically modifying microorganisms to produce PHA more efficiently, leading to reduction of the production costs.

## 1. Introduction

In the view of sustainable materials, bioplastics production can generate substantial interesting remark from renewable resources [1,2]. Polyhydroxyalkanoates (PHAs) stored by many microorganisms as storage materials assure biodegradable alternatives for conventional polymers [3,4]. Because of some specific properties, such as biodegradability, biocompatibility, and water resistance, PHAs can be employed in various disposable packaging and can have high-value healthcare and medicare [5,6,7]. Choosing the right carbon substrate is the most essential factors for increasing PHA production cost-effectively. A significant by-product of biodiesel production is glycerol, which acts as affordable carbon sources to synthesize biochemicals and biomaterials [8,9,10]. For 10 tons of produced biodiesel, approximately 1 ton of crude glycerol with 85% glycerol can be obtained from the waste-stream of 10-ton-biodiesel production [9,11]. PHA manufacturing from glycerol is one of the alternatives to recover the impact of high production cost PHA and can be ideal for PHA synthesis. *Cupriavidus necator* is a gram-negative bacteria that convert sugars, organic acids, and also glycerol to poly(3-hydroxybutyrate) (PHB), which is accumulated as microscopic granules inside the microorganism [12,13,14,15]. Recently, the deciphering of the entire 7,416,678-bp genome of *C. necator* was completed [1,16]. A disproportionately large fraction of the total coding capacity is devoted to transport systems. *C. necator* utilizes a wide range of organic carbon and energy sources for heterotrophic growth, including tricarboxylic acid cycle (TCC) intermediates, fatty acids, sugar acids, amino acids, and others [16]. From our previous study [17], there is a problem posing a challenge to the microbial biosynthesis of PHA from the glycerol. We found that PHA has low productivity when producing by *C. necator* grown on glycerol with a low specific growth rate while compared to glucose-based PHA formation. The study also suggested that the protein expression representing the central metabolic pathways was not markedly different in cells which were lithotrophically grown on CO_2_/H_2_ or chemotrophically grown on glycerol. *C. necator*, therefore, synthesized glucose-like intermediates from glycerol through a gluconeogenesis pathway, an essential metabolic pathway for biosynthesis of cellular components like glucose from non-carbohydrate precursors (i.e., acetate, lactate, formate, and glycerol) [18]. This occurrence reduces the cell dry weight and PHA productivity, especially in the high cell density growth phase. The neo-glucose intermediates would be synthesized via gluconeogenesis while the cells stop growing under a nutrient limitation or other physiological stresses.

This research investigated the limitations of fermentation cultures grown on glycerol and found evidence to overcome this PHA reduction from gluconeogenesis by using proteomic examination to elucidate metabolic pathways and protein regulations relating to glycerol utilization in bacterial synthesis. *C. necator* cells were collected at various time points with different amounts of glucose expression. Nowadays, proteomics could determine the entire protein complement of a cell, tissue, or organism under a specific, defined set of conditions [19]. A number of previous research have investigated the protein regulations during the *C. necator* grown on various carbon source exposure such as formic acid [20], gas mixture of H_2_/O_2_/CO_2_ [18], short chain organic acid [21], as well as levulinate and glucose [22] via proteomic examination. In this study, a proteomic examination was employed to elucidate the key proteins regulating the pathway shift when the bacterial cells grown on glycerol started producing glucose for maintaining cell growth and PHA formation. Then, the proteomic study could compare and examine differential protein expressions that occur during gluconeogenesis pathway-shift based on MS-based protein profiling. Several proteins induced in *C. necator* were determined using one-dimensional SDS-polyacrylamide gel electrophoresis (1-D PAGE) and liquid chromatography tandem mass spectrometry (LC-MS/MS). The vital specific proteins important in the pathway mapping were validated in two methods: bioinformatics validation and experimental validation. Additionally, we used the information obtained from LC-MS to confirm bioinformatic validation and examined differential protein expressions that occur during gluconeogenesis pathway-shift based on MS-based protein profiling. Furthermore, we determined the gluconeogenesis correlation during simultaneous occurrences of cell growth and PHA formation from glycerol in the *C. necator* cells.

## 2. Materials and Methods 

### 2.1. Bacterial Strain and PHA Synthesis

**Figure 1:**
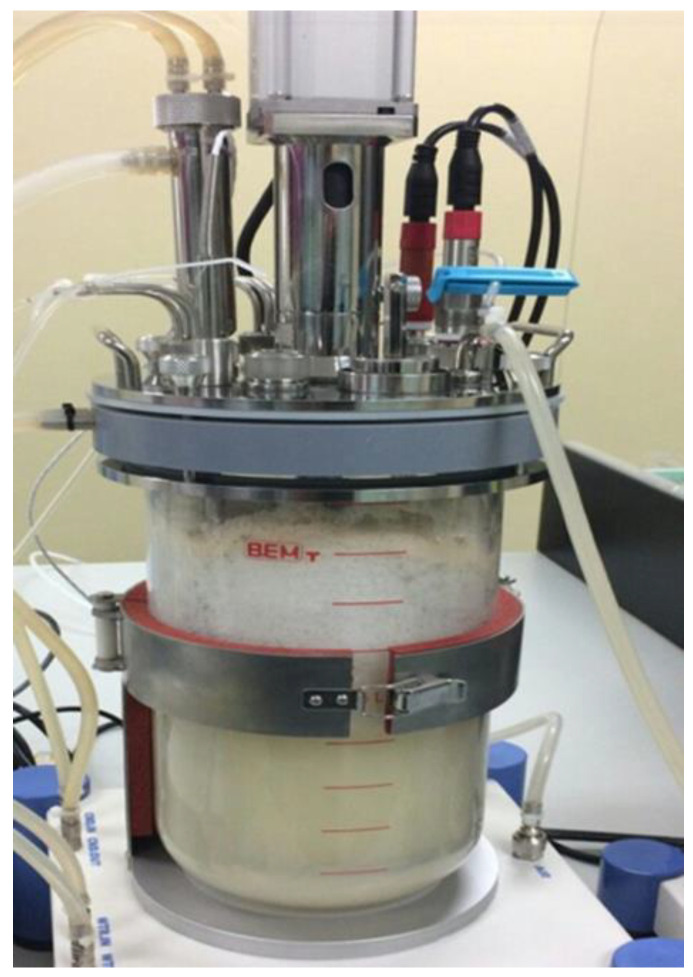
*C. necator* cells during fed batch cultivation.

As described in the previous research [17], a laboratory mutant strain of *Cupriavidus necator* (ATCC 17699) was used in this study. The strain was adapted in a glycerol-rich environment and can effectively form PHA from glycerol. An inoculum of *C. necator* was prepared by transferring the cells from a slant into 5 mL of nutrient-rich medium in a test tube and incubated for 48 h at 30 °C. The cells were cultivated in a flask containing 200 mL of a mineral solution supplemented with 20 g/L glycerol and shaken at 30 °C and 200 rpm for 36 h. The flask culture was repeated two times to obtain high-active cells as the inoculum for the glycerol fermentation in a bench-top bioreactor. Fed-batch fermentation was carried out in a 5 L bioreactor (BE Marubishi, Japan), monitored and controlled the temperature, pH, and dissolved oxygen. The culture conditions were maintained at 30 °C and pH 6.8. The cultivation was started with 2.5 L of the mineral solution with 20 g/L glycerol and 250 mL of inoculum, as evaluated from the previous research [17]. After the cell density reached the desired level, two glycerol solutions were used to keep the residual glycerol concentration above 10 g/L. As the glycerol was utilized, the medium pH declined and maintained at the desired level of pH 6.8 by adding two base solutions, depending on the nitrogen nutrient control. When the cell density reached the desired level of OD_620_ at 60–80, and no additional nitrogen was needed, the ammonia nitrogen solution was replaced with a NaOH/KOH solution to initiate nitrogen limitation for PHA accumulation. The cells were collected for cell/PHA characterization and proteomic analysis at various time points depending on different amounts of neosynthesized-glucose starting from feeding time until OD_620_ or cell density started decreasing, as shown in Figure 1.

### 2.2. Analytical Procedures

Aliquots of 30 mL culture medium were taken in pre-weighed polypropylene centrifuge tubes. Microbial growth was monitored with a UV/Vis spectrophotometer by measuring the optical density of culture medium at 620 nm. The medium was centrifuged at 5000× *g* for 10 min to separate the supernatant (culture solution) from wet pellets (PHA-containing cells). The wet pellets were subsequently washed with distilled water two times and separated for two measurements: (1) lyophilized to determine the dry cell mass and PHA content and to purify the PHA polymer; and (2) stored at −80 °C for proteomic examination. The supernatant solution was fractionated and lyophilized in order to be dissolved in deuterated water (D_2_O) for nuclear magnetic resonance (NMR) analysis to identify the metabolic intermediates released by the cells as described in the previous report [17].

The PHA content was determined with a gas chromatographer (Shimadzu GC-174, Shimadzu Corporatoin, Japan) equipped with an FID detector. Briefly, approximately 50 mg of PHA-containing dry biomass were weighed, mixed with 2 mL of methanol solution (H_2_SO_4_ 3% *v*/*v*; Benzoic acid 10 mg/mL as the internal standard) and 2 mL of chloroform, and incubated at 100 °C for 240 min. The extracted polymer was then subjected to a methanolysis reaction to form methyl ester monomers. The mixture was then cooled to room temperature, 1 mL of distilled water was added, and the solution was vortexed for 1 min. The mixture was separated into an aqueous and an organic phase. The organic layer (1 mL) was removed, filtered (PTFE membrane, 0.45 mL), and then subjected to GC analysis [23].

The purified polymers that accumulated in the cells were extracted with chloroform (10 mL chloroform/1 g dry cells) for at least 48 h at 60 °C and purified by precipitation with methanol two times. Then, they were analyzed to obtain the chemical structure with NMR spectroscopy in a CDCl_3_ solution by dissolving PHA in the D-Chloroform solution (10 mg/mL) via mixing and mild heating.

### 2.3. Protein Assay with 1-D SDS–PAGE

As the first step, the SDS-PAGE gel was prepared. SDS-PAGE gel preparation was performed by two gel preparations: resolving gel and stacking gel. The resolving gel was prepared by mixing the following solutions in sequence: 3.125 mL 40% monomer (acrylamide), 2.5 mL 0.5% Tris-HCl pH 8.8, 125 μL 10% SDS, 4.225 mL distilled water, 50 μL 10% APS and 3.3 μL TEMED to make Gel solidification. The mixture was left to form a gel for 45 min. The preparation of stacking gel was carried out by mixing the following substances: 40% monomer (acrylamide) solution (400 μL), 5% Tris-HCl pH 6.8 742 μL, 10% SDS 30 μL, 1816 μL distilled water, 10% APS solution (23 μL), and 1.7 μL TEMED. The mixture was left to form gel for 15 min. The 1-D SDS–PAGE gel run was performed by taking 10 μL of samples prepared at −20 °C with the addition of 5 μL 5× SDS loading dye and boiling in water for 10 min.

The 13 μL sample was loaded and tuned at 200 V and 20 mA for approximately 1 h 30 min. The loaded sample was stained with Coomassie blue R250 for 2 h and then destain until the background faded. The dyed samples were scanned under the GS-710 calibrate scanner for gel digestion before being imported into the LC-MS. Gel digestion was performed by bringing the cut-down gel to destain. The sample was destained with 50% methanol + 10 mmol Ambic 200 μL, which was shaken for 20–30 min, then 200 μL of Acetonitrile (ACN) was added to dehydrate gel, and it was left at room temperature for 5 min. It was absorbed and left until dry. The disulfide bond was removed by adding 10 mm of DTT and 200 μL of Ambic, incubated at 56 °C for 1 h, and the solution was extracted. Ten-millimole samples of 200 μL of DTT and Ambic were incubated at room temperature in the dark for 1 h to alkylate protein to prevent disulfide bonds. The solution was extracted after incubation and replaced with a 200 μL CAN and left at room temperature for 5 min before the solution was rinsed again. The sample was added to 200 μL of 100 mmol Ambic, left at room temperature for 10 min, and removed. The sample was added to 200 μL of ACN, left at room temperature for 5 min, and removed. Samples were added to a concentration of 200 mM of trypsin and 10 mmol Ambic enzyme per gel and 20 μL of 10 mmol Ambic and incubated at 37 °C for 24 h. The incubation sample was added to 30 μL of 10 mmol Ambic and shaken for 30 s. The shaken solution was put into a tube and dried at 40 °C for 24 h. The tubes were kept at −20 °C until further LC-MS analysis. The samples prepared at −20 °C were dissolved by 0.1% formic acid 25 μL, and then 10 μL of the solution was sucked into the vial in the LC-MS machine, where the column used was monolytic column.

### 2.4. Analysis of Proteomic Information

Protein identified with LC-MS were used for analysis with multiple array viewer (MEV) program. The KMS model was used to determine the correlation of the proteins that were expressed by the increased PHA content of GC investigations. The analyses were also used to determine the relationship between proteins and the amount of glucose monitored with NMR. The protein types were compared to the expressions related to PHA and glucose through the information stored in the database. The proteins associated with these substances were analyzed with a Stitch program. The Stitch program was used to analyze the substance-protein cross-link, which was created as a chain diagram of the proteins. The analyses were used to determine the expressions linked to glucose and glycerol for further analysis.

### 2.5. Statistical Analysis 

Data were analyzed using the GraphPad Prism software version 5.0 (GraphPad; San Diego, CA, USA). A paired *t*-test was used for comparison of two datasets. For the dataset with more than two samples, one-way analysis of variance followed by Tukey’s multiple comparison test was used. For both analyses, differences between means at the 5% confidence level (*p* value < 0.05) were considered statistically significant.

## 3. Results

### 3.1. PHA Synthesis from C. necator with Glycerol as a Carbon Source

**Figure 2:**
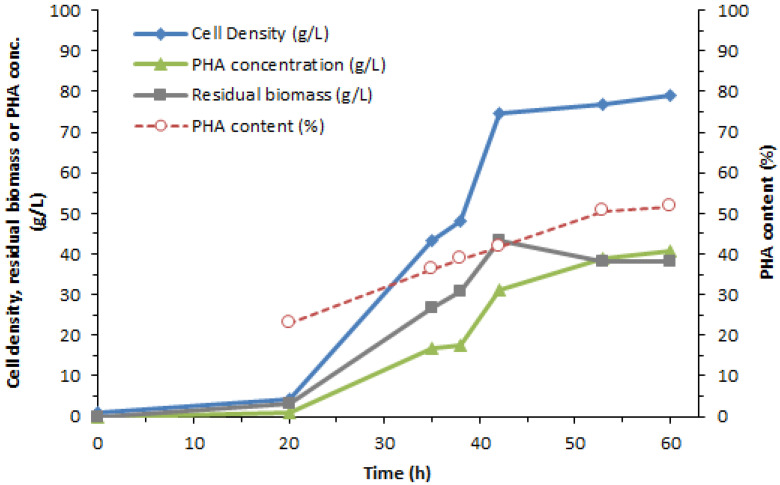
The relationship between PHA yield, production rate, and time.

Table 1 shows the different quantities obtained from the synthesis of PHA: cell density, PHA content, and rate of production. Such data can be displayed as a graph of the relationship with time as shown in Figure 2.

Table 1 exhibited the increased glucose content during the synthesis phase. The glucose content in the culture medium was examined with NMR, which is shown in Figure S1, in which the chemical shifts from the other glucose-compound peaks and the prominent glucose-compound peaks at multiplets between 3.4 to 3.9 ppm have been illustrated. Furthermore, the amount of glucose shown in Table 1 is derived from the calculation of the area beneath the glucose curve relative to deuterium oxide (D_2_O) from Figure S1. 

**Figure 3:**
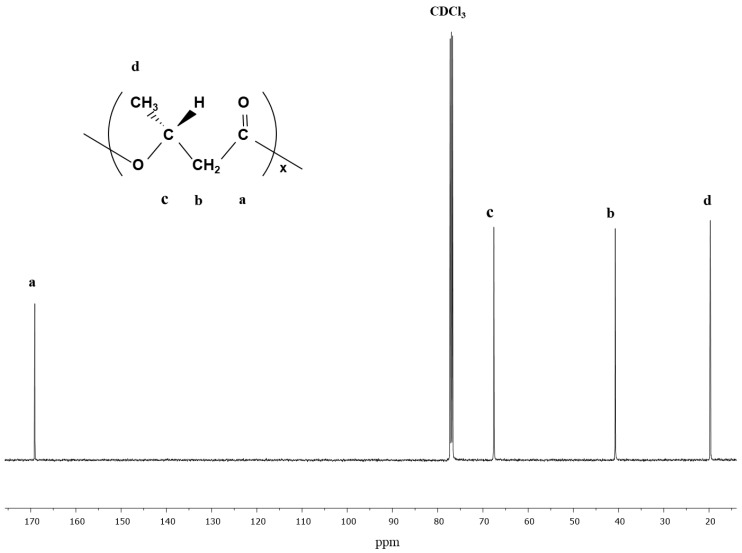
^13^C NMR spectrum of glycerol-based PHB harvested from *C. necator*.

In Figure 3, the data from carbon-NMR of purified PHA from *C. necator* cultured on glycerol in the fermentation. The spectrum shows the resonances for PHB, as described by the methyl group around 20 ppm, the methylene group around 41 ppm, the methine group around 68 ppm, and the carbonyl group around 169 ppm.

### 3.2. Proteomic Analysis of C. necator during PHA Synthesis

**Figure 4:**
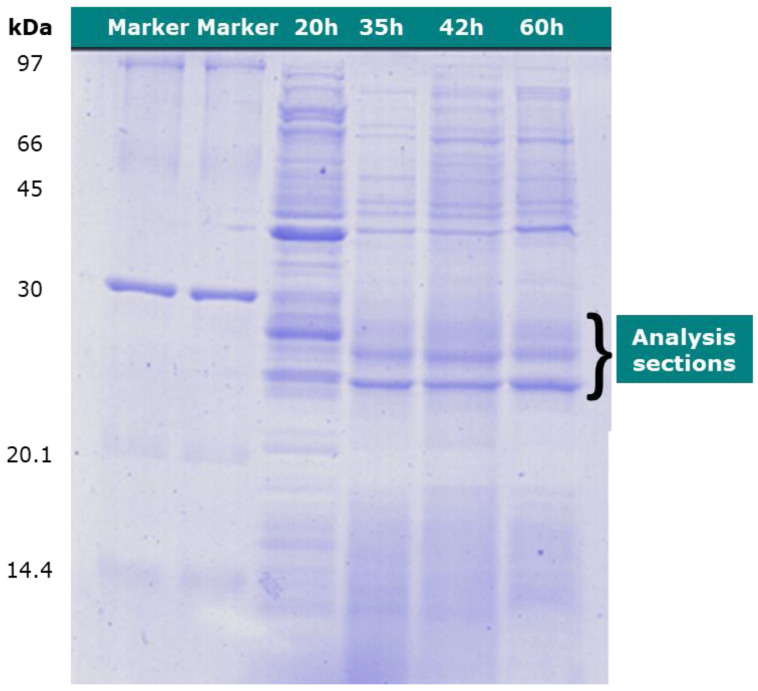
SDS-PAGE of *Cupriavidus necator* proteins sampled at 20, 35, 42, and 60 h, respectively.

The protein-type analysis was performed using LC-MS. The culture medium at 20, 35, 42, and 60 h were examined by cutting sections at approximately 30 kDa of the protein marker, as shown in Figure 4.

**Figure 5:**
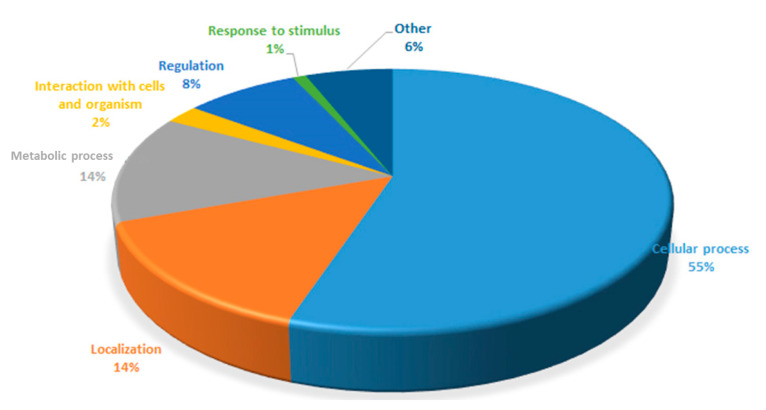
Proteins analyzed from the *C. necator* classified according to different expressions.

The protein analysis inside *Cupriavidus necator* bacterial cells was elucidated that 1361 proteins with different expressions, as shown in Table S1, were confirmed in this study. They can be divided by the protein expression according to the diagram in the Figure 5.

In total, 1361 different expressed proteins were expressed in terms of PHA production at 20, 35, 42, and 60 h. The generated 21 proteins listed in Table 2 were associated with increased PHA content when comparing to from protein expression values, and the protein expression was related to the pathway for the PHA formation.

**Figure 6:**
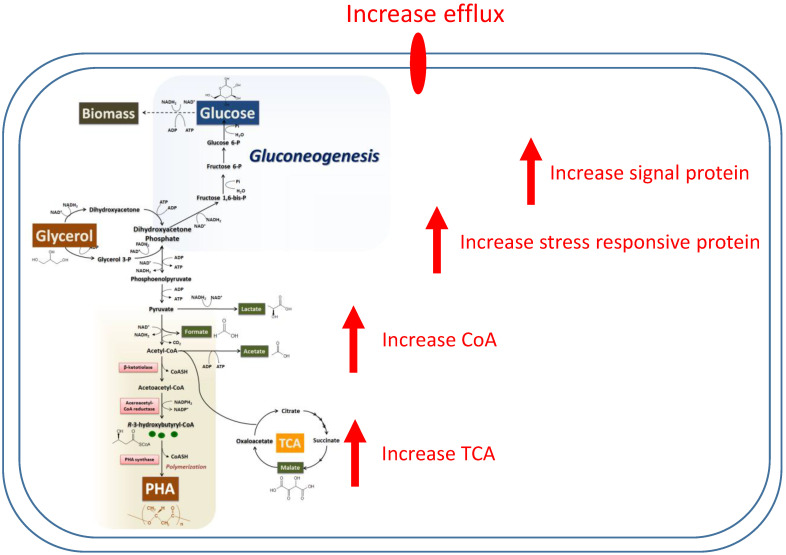
Quantitative map of the proteomic analysis of 1361 differential expressed proteins extracted from whole cells of *Cupriavidus necator* growth on glycerol during polyhydroxyalkanoate formation and a model of the cells during the PHA production period.

The expression of many proteins is associated with an increase in PHA synthesis within the production pathway. Figure 6 provides a schematic diagram of an increase in the PHA synthesis pathway based on protein expression.

**Figure 7:**
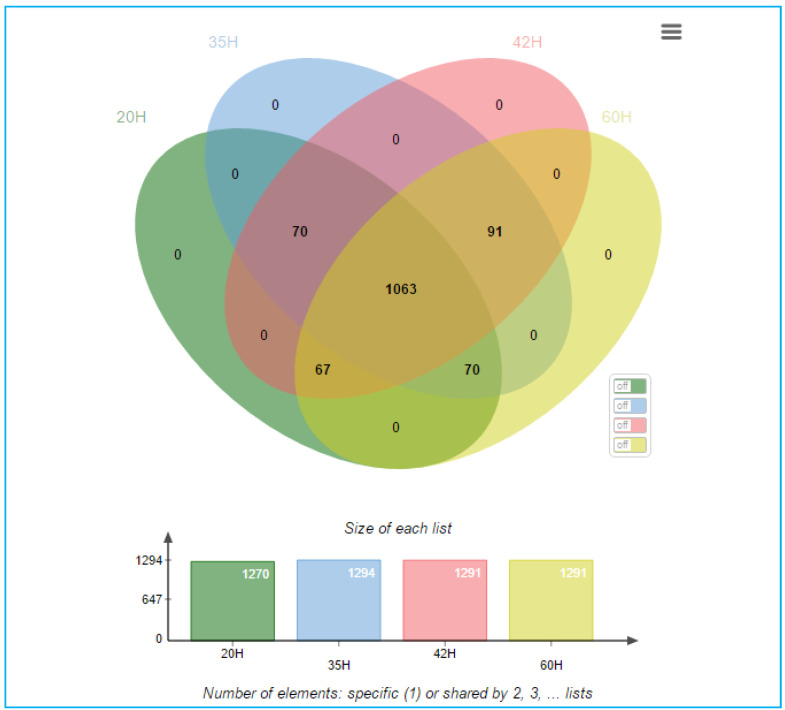
Venn diagram shows the number of differentials expressed proteins found between groups of each cultivation time of 20, 35, 42, and 60 h.

**Figure 8:**
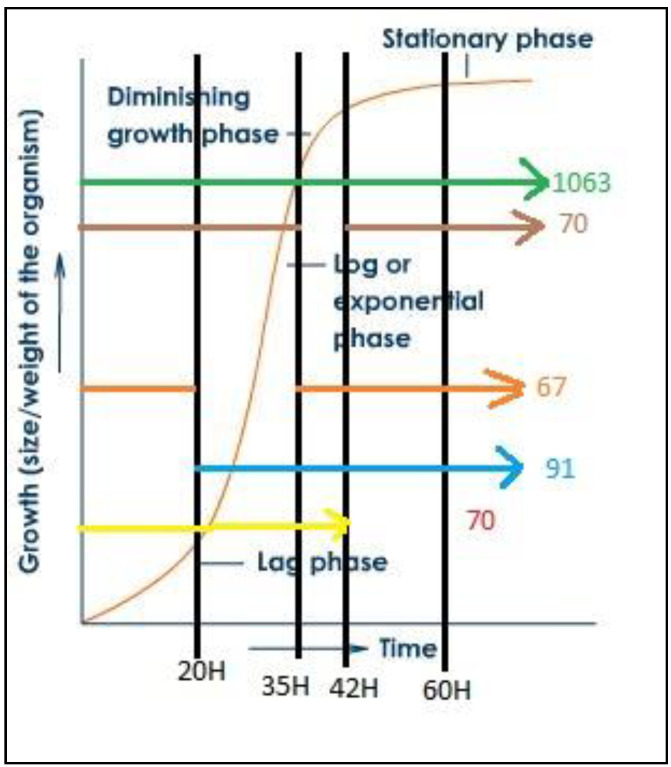
Growth curve of *C. necator* and expressed protein amount at each cultivation time.

In addition, the 1361 detected proteins were analyzed for their relevance to the continuous production of PHA and glucose due to the presence of some proteins that were not expressed at any time other than the initial period. The analysis was performed through a multiple array viewer (MEV) program to determine the correlation of expressions with both positive and negative PHA and glucose. The alignment of protein clusters was relative to increased PHA content. The clustering of proteins relative to the increased content of PHA was performed using the KMS analysis model through the MEV program, as shown in Figure S2. Protein selection was performed by J VENN method. The Venn diagram shows the number of differentially expressed proteins at each time as shown in Figure 7, where the time of interest was 35 h, which was the growth phase of *C. necator* and PHA was accumulated, as shown in Figure 8.

Analysis of the MEV program found that the first group of 93 proteins had expression patterns in the same direction as PHA, as shown in the Table S2, while six proteins that were expressed in the same direction as the amount of glucose increased in the culture medium.

**Figure 9:**
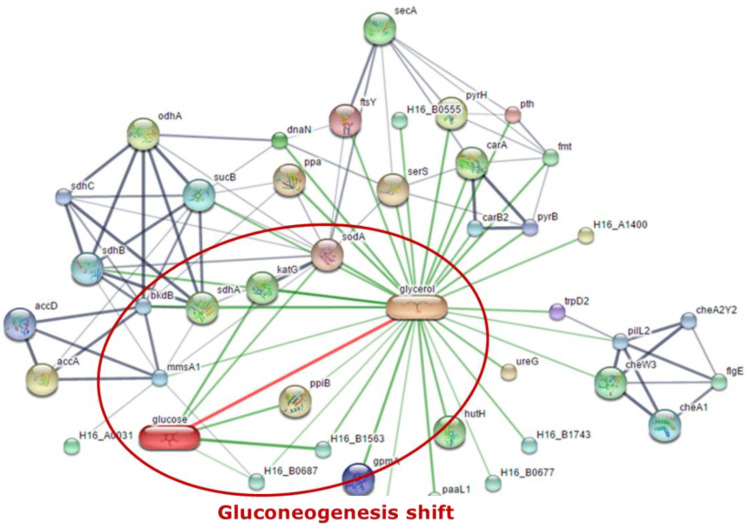
Linkage of the two protein groups was obtained through the Stich program.

MEV analysis data were used for further analysis with Stich program. The program was able to find the link between proteins associated with the expressed protein relative to the protein profile in the previously studied database. This study analyzed the expressed proteins binding to glycerol and glucose, which signaled to influence the glucogenesis that occurs in the PHA pathway produced from glycerol. The pathway link diagram is shown in Figure 9, and five proteins that are linked between glycerol and glucose were observed as follows: glycosyl hydrolase (H16_B1563), short-chain dehydrogenase (H16_B0687), superoxide dismutase (sodA), catalase (peroxidase I) (katG), and peptidyl-prolyl cis-trans isomerase (ppiB).

Stitch 4.0 software (http://stitch.embl.de, Heidelberg, Germany) was applied to predict the biological protein–protein interaction networks of the differential expressed proteins found between groups of each cultivation time. The thicker lines are used to present the associations that are stronger. The blue line represents protein–protein interactions, green line shows chemical–protein interactions, and the red line indicates an interaction between chemicals. As shown in Figure 9, five binding proteins associated between glycerol and glucose give rise on two proteins that were involved in *Cupriavidus necator*: glycosyl hydrolase (H16_B1563) and short-chain dehydrogenase (H16_B0687).

## 4. Discussion and Conclusions

PHA synthesis by *C. necator* from glycerol as a carbon source has been reported in our previous studies [17]. The results of PHA synthesis from these strains show that the increased PHA content was associated with the increased time of fermentation. However, the synthesis of *C. necator* by glycerol yielded less PHA than glucose synthesis. Analysis of the culture medium with NMR showed that the glucose peak increased over time. Therefore, it can be indicated that glycerol used as a carbon source of the PHA synthesis pathway has glucogenesis-shift, which causes the PHA content to decrease.

Protein analysis of *C. necator* by LC-MS revealed 1361 proteins in the culture medium. Analysis of protein expression associated with increased PHA content revealed 21 species with the most similar function to the PHA synthesis pathway. These proteins have the effect of increasing enzymes that influence the synthesis pathways. In addition, the proteins that were expressed at each time of the PHA synthesis were not expressed at the time of substantially high cell density at the end of the synthesis. This indicates that there are positive and negative correlations with the PHA synthesis and the 298 candidate proteins. The results of the clustering of proteins using the multiple array viewer (MEV) program and using the KMS data analysis model were found to contain 93 proteins. Proteins with the same expression pattern for PHA and six proteins with similar expression patterns were found to be correlated with generating glucose content. By the Stitch program, the proteins of interest were found with research data linked between glycerol and glucose. Five protein types are connecting to glucose and glycerol shift pathway, two of which are glycosyl hydrolase (H16_B1563) and short-chain dehydrogenase (H16_B0687).

Glycosyl hydrolase (H16_B1563) is an enzyme group involved in complex sugar hydrolysis reactions [24]. It is associated with the conversion of glycerol to glucose due to the degradation of the double-chain sugars with shorter molecular chain lengths. While short-chain dehydrogenase (H16_B0687) is a group of enzymes involved in the breakdown of short-chain sugars. It signals that sugar bonds are disintegrated following glycosyl hydrolase (H16_B1563) degradation and is in the process of transition to glucose as a single sugar molecule. Both of those are enzymes used to break the bonds of complex sugars, possibly related to the partial conversion of glycerol to glucose. Based on the linked protein data, it was assumed that both proteins had the effect of converting glycerol into glucose, gradually breaking down the bonds of the complex sugars until their molecular chains were shortened. As a result, the amount of PHA synthesized with glycerol decreased. Both the protein data may be useful in future modifications to optimize PHA pathways.

In conclusion, the investigation of these associated proteins can be obtained useful information in the pathway analysis of PHA synthesis. Genetic modifications of *C. necator* to inhibit the activity of the analyzed proteins may improve PHA productivity. In this study, it was assumed that the proteins could accelerate the digestion of complex sugars like glycerol, resulting in a decrease in the PHA synthesis. The reduced bond breakdown can cause glycerol to be used more in the PHA synthesis pathway and reduced glucogenesis shift, which causes the PHA formation to increase. Based on the above analysis, necessary information on the protein expression associated with PHA was found to be linked among the active carbon source, glycerol, and glucose generation. It is possible that other low-cost carbon sources, such as wastewater or industrial wastes, may encounter the same problem. Finding the associated expression proteins would provide useful information for genetically modifying microorganisms to produce PHA more efficiently, leading to industrial production that can compete with conventional plastics.

## Figures and Tables

**Table 1:** PHA content, biomass and synthetized glucose measured during fermentation of *C. necator*.

Cultivation Time (h)	Cell Dry Mass (g/L)	PHA Content (%)	PHA Concentration (g/L)	PHA Productivity (g/L/h)	Glucose/D_2_O NMR Area Ratio
0	0.8	ND	ND	ND	ND
20	4.1	23.0	0.9	0.05	0
35	43.5	36.3	16.8	0.48	1.13
38	48.2	18.7	17.5	0.46	ND
42	74.7	41.8	31.2	0.74	2.26
53	77.0	50.6	38.9	0.73	ND
60	78.9	51.6	40.7	0.68	4.55

**Table 2:** The generated 21 proteins that expressed level similar to PHA productivity in the fermentation.

No.	Protein Name	Accession Number	Function
1	methylmalonate-semialdehyde dehydrogenase (Jannaschia sp. CCS1)	gi|89054404	Amino acid metabolism
2	acyl-CoA dehydrogenase domain-containing protein (Methylobacterium radiotolerans JCM 2831)	gi|170749664	Lipid metabolism
3	succinate dehydrogenase flavoprotein subunit (gamma proteobacterium IMCC1989)	gi|497354190	Respiration
4	capsular exopolysaccharide family protein (Thioalkalivibrio sp. K90mix)	gi|289207806	Signal
5	integral membrane sensor signal transduction histidine kinase (Burkholderia sp. CCGE1002)	gi|295676684	Signal
6	membrane protease subunit stomatin/prohibitin-like protein (Magnetospirillum magneticum AMB-1)	gi|83312588	Signal
7	protein YhiI (Pasteurella multocida subsp. multocida str. HN06)	gi|383310937	Signal
8	glycosyl transferase family protein (Burkholderia vietnamiensis G4)	gi|134291866	Signal
9	short-chain dehydrogenase (marine gamma proteobacterium HTCC2143)	gi|494429549	Signal
10	phosphoserine phosphatase SerB (Methylotenera versatilis 301)	gi|297537989	Stress response
11	superoxide dismutase (Burkholderia cenocepacia AU 1054)	gi|107023480	Stress response
12	beta-lactamase class C-like protein (Shewanella amazonensis SB2B)	gi|119774488	Stress response
13	chemotaxis protein (Burkholderia thailandensis E264)	gi|83717148	Stress response
14	preprotein translocase subunit SecA (Idiomarina baltica)	gi|494014389	Transport
15	ABC-type transporter, auxilary periplasmic component involved in toluene tolerance (Ralstonia eutropha H16)	gi|113869370	Transport
16	type II secretion system protein E (Pectobacterium carotovorum subsp. carotovorum PC1)	gi|253687071	Transport
17	hypothetical protein (Vibrio mimicus)	gi|445940548	Unknown
18	HrpA protein (Neisseria gonorrhoeae NCCP11945)	gi|194099492	Unknown
19	hypothetical protein G157_07140 (Campylobacter coli CVM N29710)	gi|543941344	Unknown
20	flagellar scaffolding protein FlgD (Loktanella vestfoldensis)	gi|494388140	Unknown
21	hypothetical protein (Candidatus Regiella insecticola)	gi|493755517	Unknown

## Supplementary Materials

The following are available online at https://www.mdpi.com/2306-5354/7/4/154/s1, Table S1: The protein analysis inside *Cupriavidus necator* cells discovered 1361 proteins with different expressions. Table S2: Analysis of the MEV program of 93 proteins having expression patterns in correlated with glucose or PHA formation. Figure S1: NMR spectra of culture medium showed glucose peak during PHA synthesis. Figure S2: Cluster of proteins using the multiple array viewer (MEV) program using the KMS data analysis model. The above is for PHA synthesis correlation, while the below is for the glucose synthesis correlation.
