# Supplementary material for: Proteomic Examination for Gluconeogenesis Pathway-Shift during Polyhydroxyalkanoate Formation in Cupriavidus necator Grown on Glycerol
Source: Bioengineering (Basel). 2020 Dec 1;7(4):154. doi: 10.3390/bioengineering7040154 (PMC7712004; doi:10.3390/bioengineering7040154)

Supplementary Figure

Proteomic Examination for Gluconeogenesis Pathway-Shift during Polyhydroxyalkanoate Formation in *Cupriavidus necator* Grown on Glycerol

Nuttapol Tanadchangsaeng [1,*] and Sittiruk Roytrakul [2]

[1] College of Biomedical Engineering, Rangsit University, 52/347 Phahonyothin Road, Lak-Hok, Pathumthani 12000 Thailand; nuttapol.t@rsu.ac.th

[2] Proteomics Research Laboratory, National Center for Genetic Engineering and Biotechnology (BIOTEC), 113 Thailand Science Park, Khlong Luang, Pathumthani 12120 Thailand; sittiruk@biotec.or.th

***** Correspondence: nuttapol.t@rsu.ac.th; Tel.: +66-(0)2-997-2200 ext. 1428, Fax: +66-(0)2-997-2200 ext. 1408

Supplementary Figure

FIGURE CAPTIONS

Figure S1 NMR spectra of culture medium showed glucose peak during PHA synthesis.

Figure S2 Cluster of proteins using the multiple array viewer (MEV) program using the KMS data analysis model. The above is for PHA synthesis correlation, while the below is for the glucose synthesis correlation.

Supplementary Figure

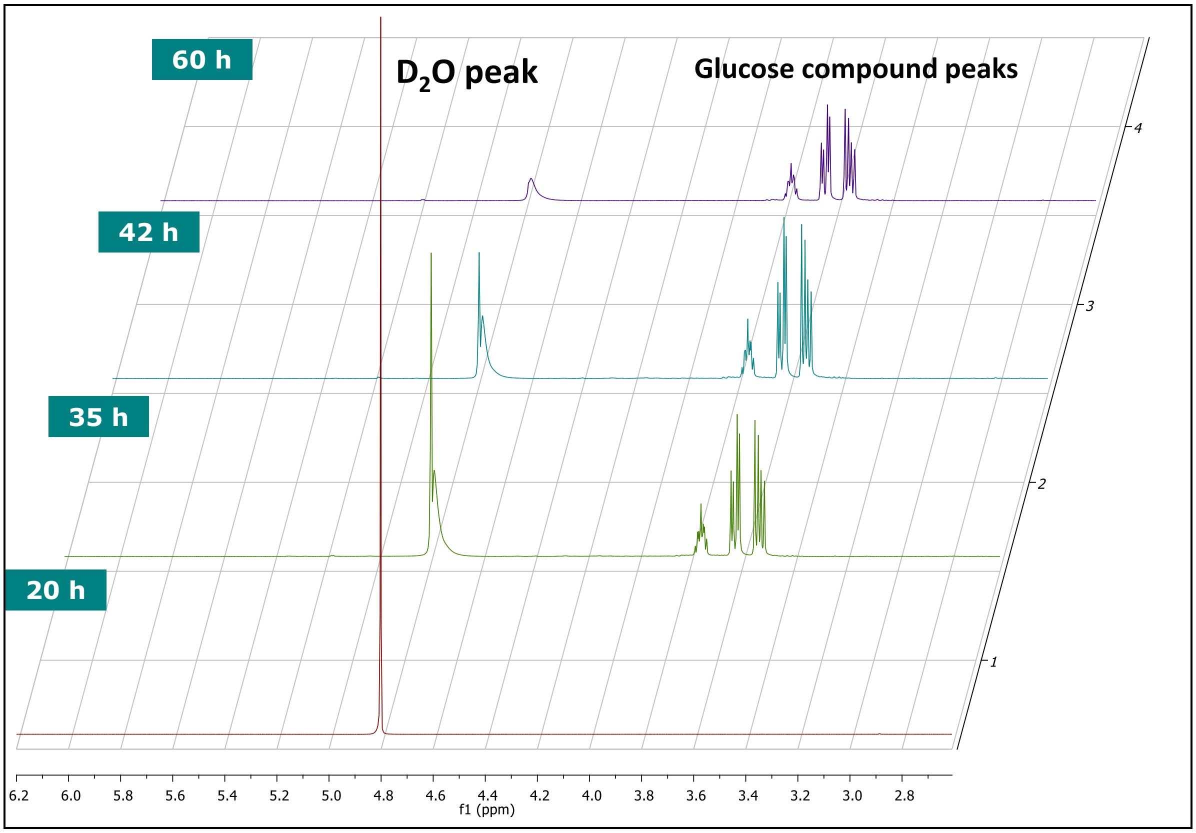

Figure S1 Tanadchangsaeng et al.

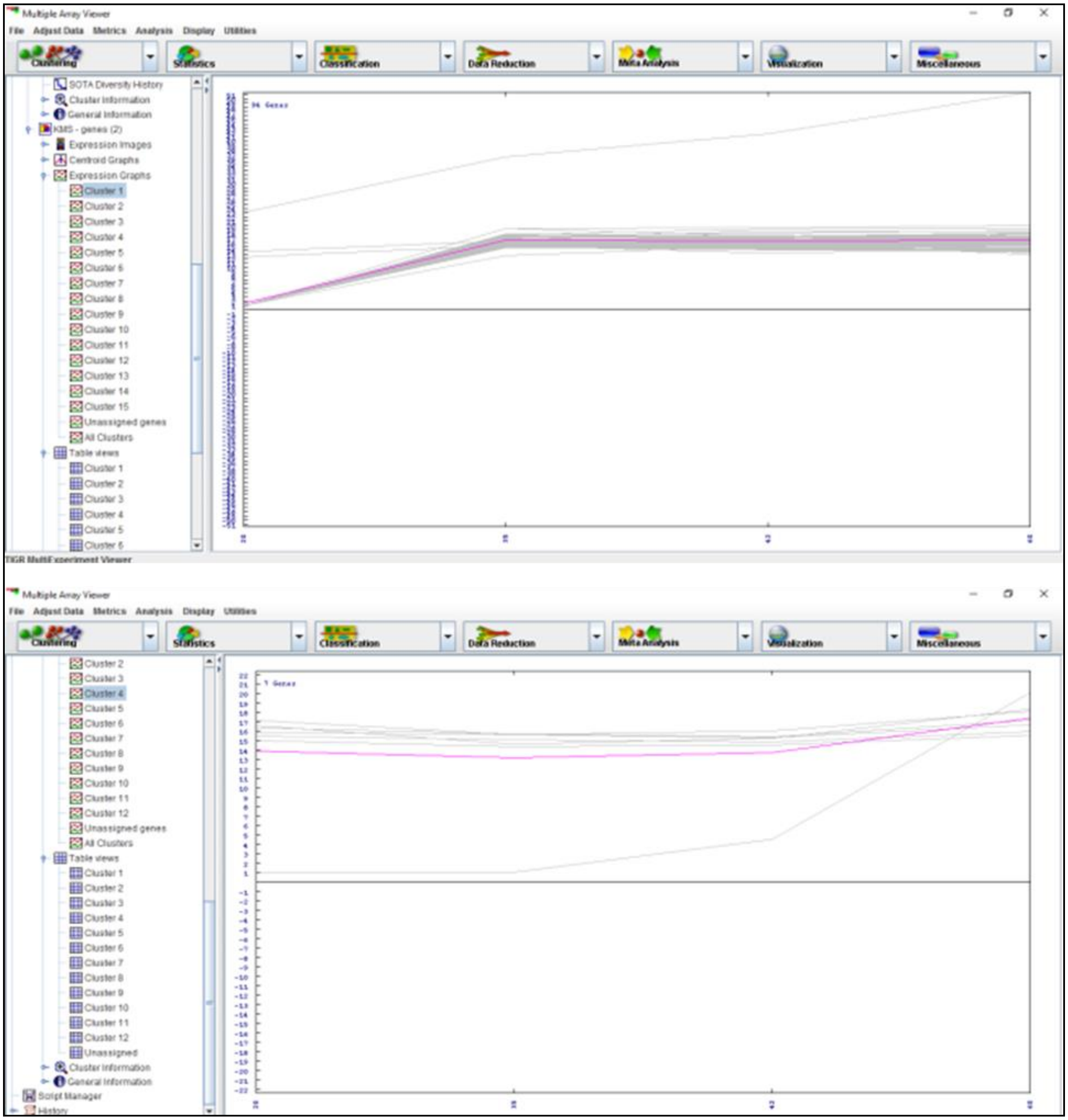

Figure S2 Tanadchangsaeng et al.

Table S1: The protein analysis inside Cupriavidus necator cells discovered 1361 proteins with different expressions.

Number	Protein name	Accession numbe	ID Score	Peptide	20H	35H	42H	60H	35H/20H	42H/20H	60H/20H
1	cytochrome b/b6-like protein [Sphingopyxis alaskensis RB2256]	gi|103486830	0.589999974	SAATA	17.2005	16.3885	16.00849	16.45532	0.95279207	0.930699108	0.956676841
2	glucose-6-phosphate isomerase [Pseudomonas entomophila L48]	gi|104783681	9.350000381	EAFAADGK	16.39732	15.47513	15.28156	15.7844	0.943759712	0.931954734	0.962620721
3	superoxide dismutase [Burkholderia cenocepacia AU 1054]	gi|107023480	44.36000061	KADGSLDIVSTSNAATPLTTADK	0	16.6045	16.76372	17.1114		1.009588967	1.030527869
4	lysyl-tRNA synthetase [Burkholderia cenocepacia AU 1054]	gi|107028702	14.14999962	TIASIR	16.16004	15.55554	15.78673	15.61882	0.962592914	0.976899191	0.966508746
5	hypothetical protein MXAN_6470 [Myxococcus xanthus DK 1622]	gi|108758819	0.800000012	VEGPPR	17.53032	17.00232	14.83054	17.93197	0.969880755	0.845993684	1.022911732
6	beta-lactamase [Myxococcus xanthus DK 1622]	gi|108759551	20.37999916	ARLDMPLSEVIPRSLLADATAGR	16.29893	13.76899	13.73833	0	0.844778768	0.842897663	0
7	phage integrase site specific recombinase [Myxococcus xanthus DK 1622]	gi|108761635	21.54000092	KSAAHKR	16.93215	15.83266	15.95003	16.28379	0.93506495	0.941996734	0.961708348
8	putative invasin [Yersinia pestis Antiqua]	gi|108809764	21.54999924	LTHIKAGESGILAR	17.28456	16.47659	15.98208	15.84568	0.953254812	0.924644885	0.916753449
9	riorf43 [Agrobacterium rhizogenes]	gi|10954689	9.960000038	ATSAKLVAGAITDLNMMAKR	15.53593	14.01532	14.21605	14.76262	0.902123014	0.915043387	0.950224415
10	EtpD [Escherichia coli O157:H7 str. Sakai]	gi|10955268	9.109999657	HASAEK	16.75799	15.95015	15.0434	16.14315	0.951793741	0.897685224	0.963310636
11	GA24 protein	gi|1096270	73.94000244	ANLETLFGLTTK	22.38636	20.44584	20.90705	20.95085	0.913316859	0.933919136	0.935875685
12	DNA mismatch repair protein MutL [Pseudoalteromonas atlantica T6c]	gi|109900275	1.74000001	GGHKR	16.95969	15.35283	15.36995	15.98339	0.905254164	0.906263617	0.942434089
13	NERD domain-contain protein [Pseudoalteromonas atlantica T6c]	gi|109900397	18.82999992	QMQNGLPPTTR	18.21003	16.90232	16.89584	17.17192	0.928187378	0.92783153	0.942992406
14	peptide ABC transporter periplasmic protein, partial [Helicobacter acinonychis str. Sheeba]	gi|109946854	8.319999695	MMVLDKYR	16.54361	15.27732	15.4124	15.62713	0.923457456	0.931622542	0.944602176
15	phage transcriptional regulator AlpA [Chelativorans sp. BNC1]	gi|110632857	7.429999828	AKPIPR	15.05679	13.01154	15.13033	14.73301	0.864164274	1.004884175	0.978496081
16	5'-nucleotidase [Chelativorans sp. BNC1]	gi|110635059	15.14000034	ECQMGNLVTDAMLAR	18.0096	15.90002	16.49712	16.79311	0.882863584	0.916018124	0.932453247
17	spermidine/putrescine ABC transporter ATPase [Chelativorans sp. BNC1]	gi|110636068	13.39999962	TEVLAAQPELR	16.95578	13.62592	15.38067	16.30086	0.80361505	0.907104834	0.961374823
18	lytic murein transglycosylase [Roseobacter denitrificans OCh 114]	gi|110678651	4.840000153	GHAPASVDR	0	16.02721	15.75135	15.96341		0.982788021	0.99601927
19	acyl-CoA dehydrogenase [Alcanivorax borkumensis SK2]	gi|110833982	18.65999985	MTGAFGRIR	15.96983	15.51756	15.15566	15.51203	0.971679724	0.949018243	0.971333446
20	outer membrane protein OmpH [Alcanivorax borkumensis SK2]	gi|110834013	11.21000004	LKKAIESVAKR	17.28561	16.54422	17.14135	16.84954	0.957109411	0.99165433	0.974772658
21	acetylglutamate kinase [Ralstonia eutropha H16]	gi|113866239	20.64999962	MNADNPGPAATTVAAIAPALK	18.80626	16.04957	16.64484	17.03636	0.853416362	0.885069121	0.90588772
22	N-acetyl-gamma-glutamyl-phosphate reductase [Ralstonia eutropha H16]	gi|113866251	19.11000061	GLAVTVPVFTDR	16.57207	17.24876	16.07186	15.82295	1.040833161	0.969816082	0.954796232
23	organic solvent tolerance protein [Ralstonia eutropha H16]	gi|113866540	17.94000053	MFIQPSISYPIVR	17.10752	14.22188	13.64969	14.99618	0.831323301	0.797876606	0.876584099
24	ABC transporter periplasmic protein [Ralstonia eutropha H16]	gi|113866786	36.49000168	VSNPALEALGTFK	19.41727	17.0827	19.52984	20.23041	0.879768371	1.005797416	1.041877154
25	triosephosphate isomerase [Ralstonia eutropha H16]	gi|113867068	48.75	VVLAYEPVWAIGTGK	18.13315	14.27912	17.26643	17.53949	0.787459432	0.952202458	0.967261066
26	ABC-type transporter, periplasmic component [Ralstonia eutropha H16]	gi|113867414	24.88999939	GEKPGAIASQTSDNLELFVNTGAAAK	16.57651	14.70333	0	15.47928	0.886997927	0	0.933808142
27	lactaldehyde dehydrogenase [Ralstonia eutropha H16]	gi|113867899	71.75	VVEGFTVGDPLQEASK	18.42329	17.17837	18.86889	18.00678	0.932426836	1.024186777	0.977392203
28	elongation factor Ts [Ralstonia eutropha H16]	gi|113868033	22.18000031	EVSLFNQPFVK	19.39898	16.81465	17.84233	18.47851	0.866780109	0.91975609	0.952550598
29	amidase [Ralstonia eutropha H16]	gi|113868463	23.78000069	GLPNSFAGTTLADELAR	0	15.77117	15.22931	14.67993		0.965642371	0.930807924
30	ABC-type sugar transporter, periplasmic component [Ralstonia eutropha H16]	gi|113868465	7.179999828	GDPGK	16.4177	15.55863	15.09679	15.55091	0.947674157	0.919543541	0.947203932
31	outer membrane protein peptidoglycan-associated(lipo)proteins [Ralstonia eutropha H16]	gi|113868899	36.18000031	VNIPSQVTFDTDSATIKPSFR	20.08697	18.6665	19.14944	19.96802	0.929284008	0.95332646	0.994078251
32	extra-cytoplasmic solute receptor [Ralstonia eutropha H16]	gi|113868935	18.48999977	ANTLQELIAYAK	18.5272	14.20205	16.79794	17.18404	0.766551341	0.906663716	0.927503346
33	hypothetical protein H16_A3121 [Ralstonia eutropha H16]	gi|113869075	39.08000183	GPDMFDVEAFPEAVYK	15.70457	14.64777	14.49872	15.19187	0.932707486	0.923216618	0.967353452
34	ABC-type transporter, auxilary periplasmic component involved in toluene tolerance [Ralstonia eutropha H16]	gi|113869370	24.29000092	GSFNTIVGQQGVEGVIK	0	17.39219	18.03707	18.69442		1.037078712	1.036444389
35	glutamate synthase subunit beta [Ralstonia eutropha H16]	gi|113869377	10.31000042	NAKASTEGERAYHTNVPK	17.26723	14.33875	14.62058	15.25674	0.830402444	0.846724113	0.883566154
36	UbiH/UbiF/VisC/COQ6 family ubiquinone biosynthesis hydroxylase [Alkalilimnicola ehrlichii MLHE-1]	gi|114319394	14.51000023	YEARRRPANLMMMHGMDLFK	15.66336	15.4391	14.42175	14.29544	0.98568251	0.920731567	0.912667525
37	DNA polymerase subunit beta [Alkalilimnicola ehrlichii MLHE-1]	gi|114320024	13.15999985	ETGVAL	17.80774	17.16628	0	16.99697	0.963978585	0	0.954470921
38	putative mercury transport protein MerC [Nitrosomonas eutropha C91]	gi|114326590	11.26000023	CELPPK	0	15.57152	15.62759	15.9459		1.003600805	1.020368464
39	nitrogen assimilation regulatory protein, NtrC [Granulibacter bethesdensis CGDNIH1]	gi|114327890	26.93000031	ESGLPAK	18.91416	17.98663	17.635	18.08099	0.950961079	0.932370245	0.955949934
40	general secretion pathway protein N [Granulibacter bethesdensis CGDNIH1]	gi|114328428	6.210000038	SSSVA	17.08882	16.50269	15.16153	15.99922	0.965700967	0.887219246	0.936239015
41	two component system histidine kinase [Granulibacter bethesdensis CGDNIH1]	gi|114328565	17.21999931	DTGGMGLGLAIVR	20.08377	17.63911	19.09861	19.68842	0.878276837	0.950947457	0.980314951
42	oligopeptide transport system permease oppC [Granulibacter bethesdensis CGDNIH1]	gi|114328652	12.18999958	NWLGTDDRAR	17.81542	15.20609	15.3292	15.82384	0.853535308	0.860445614	0.888210326
43	thioredoxin domain-containing protein [Shewanella frigidimarina NCIMB 400]	gi|114562715	11.72999954	KLYTLLY	16.00288	13.73881	14.7312	15.3573	0.858521091	0.920534304	0.959658511
44	PTS system glucose-specific transporter [Shewanella frigidimarina NCIMB 400]	gi|114562942	16.97999954	GKVDSLLTPVVLANMEDVKQLDK	16.75677	14.16473	13.74683	13.97039	0.845313864	0.82037469	0.833716164
45	hypothetical protein Sfri_2842 [Shewanella frigidimarina NCIMB 400]	gi|114564006	19.55999947	QIAMSMVKEAK	16.76724	15.8095	15.5359	16.001	0.942880283	0.92656275	0.954301364
46	NADH dehydrogenase [Shewanella frigidimarina NCIMB 400]	gi|114564590	19.34000015	MTNIVVIGGGAGGMELLGKISK	15.40816	15.24941	15.30008	15.01138	0.989697018	0.992985535	0.97424871
47	Chain A, Heterotetrameric Sarcosine: Structure Of A Diflavin Metaloenzyme At 1.85 A Resolution	gi|114794040	13.63000011	LSAEQSSRARINREEALSLTVDGAK	15.04143	14.33759	14.49685	15.54834	0.953206577	0.963794666	1.033700918
48	D-isomer specific 2-hydroxyacid dehydrogenase family protein [Hyphomonas neptunium ATCC 15444]	gi|114797371	1.50999999	SAPGS	16.98788	15.89795	14.55557	0	0.935840729	0.856820863	0
49	isochorismatase family protein [Hyphomonas neptunium ATCC 15444]	gi|114798038	4.159999847	AWIEGYKG	16.76643	15.67972	15.75077	16.25682	0.935185367	0.939423002	0.969605336
50	inosine-5'-monophosphate dehydrogenase [Hyphomonas neptunium ATCC 15444]	gi|114800464	17.52000046	NVVTAQMDMDPAEARRLLHK	18.64223	16.46883	15.67227	15.70516	0.883415235	0.840686441	0.842450715
51	peptidase S10, serine carboxypeptidase [Burkholderia ambifaria AMMD]	gi|115359385	3.670000076	TGGHA	16.41493	17.4447	14.58977	15.23617	1.062733743	0.888810979	0.928189764
52	periplasmic sensor hybrid histidine kinase [Burkholderia ambifaria AMMD]	gi|115359890	10.53999996	RIAISQLVEPHDLAIRSDRK	16.60722	16.11792	15.47631	0	0.970536911	0.93190251	0
53	hypothetical protein Bamb_5397 [Burkholderia ambifaria AMMD]	gi|115360140	10.57999992	LAEWGVTFDDACDVAIDNLR	18.13506	0	16.70212	16.46952	0	0.920985097	0.908159113
54	F0F1 ATP synthase subunit delta [Rhodopseudomonas palustris BisA53]	gi|115522311	16.18000031	AMLAESPDLTR	17.5633	16.45101	16.42756	16.69998	0.936669646	0.935334476	0.950845228
55	histone family protein DNA-binding protein [Rhodopseudomonas palustris BisA53]	gi|115525842	21.29999924	SQLIEKIATQTELAKR	17.57419	14.62619	14.76171	15.24832	0.832254004	0.839965313	0.867654213
56	marR family transcriptional regulator [Rhizobium leguminosarum bv. viciae 3841]	gi|116248889	26.10000038	LVGTLAK	18.17269	17.13856	17.03389	17.32162	0.94309428	0.937334539	0.953167638
57	ribonuclease-L-PSP family protein [Rhizobium leguminosarum bv. viciae 3841]	gi|116250934	17.65999985	EAIFPADRHALYEKHGYSAAIR	17.62509	15.72792	15.09633	14.24403	0.892359699	0.856524988	0.808167788
58	chemotaxis two-component response regulator protein-glutamate methylesterase [Rhizobium leguminosarum	gi|116253767	18.79000092	AAGTIR	17.60881	16.8707	16.40485	16.9711	0.958082914	0.931627407	0.963784606
59	H-NS-like DNA-binding protein [Ralstonia eutropha H16]	gi|116694182	15.01000023	IEQYGLTAEDLGFGAK	16.16683	15.52891	14.53814	16.63275	0.96054143	0.899257306	1.028819503

60	fructose-1,6-bisphosphate aldolase [Ralstonia eutropha H16]	gi|116695327	22.19000053	ETYGVPVEEILR	19.9423	16.78939	19.07931	18.66286	0.841898377	0.956725654	0.935842907
61	5-methyltetrahydropteroyltriglutamate--homocysteine S-methyltransferase [Ralstonia eutropha H16]	gi|116695522	36.83000183	ALNQGDAVVAEALAASDAAQASR	19.42792	16.52209	15.0537	16.0885	0.850430206	0.774848774	0.828112325
62	hypothetical protein H16_B2196 [Ralstonia eutropha H16]	gi|116696132	3.809999943	ANGRPVQAGMTLDETTR	15.36734	13.52079	14.17178	15.19634	0.879839322	0.92220124	0.988872505
63	4Fe-4S ferredoxin [Syntrophobacter fumaroxidans MPOB]	gi|116749232	3.400000095	VDEIK	16.86323	15.49282	15.32487	16.05314	0.918733837	0.908774298	0.95196116
64	multi-sensor signal transduction histidine kinase [Syntrophobacter fumaroxidans MPOB]	gi|116750316	21.70000076	IVEIGGQLGLR	18.17714	16.74908	17.28062	17.99509	0.921436486	0.95067871	0.989984673
65	DNA mismatch endonuclease vsr [Syntrophobacter fumaroxidans MPOB]	gi|116751271	18.56999969	SRIMSRVGQK	18.01163	16.97912	16.797	17.42818	0.942675371	0.932564127	0.967607041
66	hypothetical protein Shewana3_2918 [Shewanella sp. ANA-3]	gi|117921358	9.460000038	FGELSETLSEMGLMPR	17.619	15.88332	16.47852	16.76219	0.901488166	0.935269879	0.951370112
67	molybdenum cofactor guanylyltransferase [Magnetococcus marinus MC-1]	gi|117925432	9.020000458	QTGFATCSFMRR	17.30515	15.83802	16.03485	16.28804	0.915220036	0.926594106	0.941225011
68	response regulator receiver protein [Magnetococcus marinus MC-1]	gi|117925995	3.25	MRLAA	15.64615	14.5333	14.45628	15.11635	0.928873876	0.92395126	0.966138635
69	PAS/PAC sensor hybrid histidine kinase [Magnetococcus marinus MC-1]	gi|117926231	5.130000114	GVAGR	15.846	15.67749	15.34934	16.66522	0.989365771	0.968657074	1.051698851
70	argininosuccinate synthase [Magnetococcus marinus MC-1]	gi|117927016	26.89999962	IAAMLRNGPKV	18.59797	17.54287	17.27219	0	0.943268002	0.928713725	0
71	heavy metal transport/detoxification protein [Campylobacter fetus subsp. fetus 82-40]	gi|118475757	20.94000053	VIKEID	21.35506	20.37706	19.62696	20.47843	0.954202891	0.919077727	0.958949776
72	radical SAM domain-containing protein [Pelobacter propionicus DSM 2379]	gi|118580197	4.929999828	GVPVIR	16.69548	13.76486	15.114	15.89415	0.824466263	0.905274961	0.952003177
73	outer membrane efflux protein [Pelobacter propionicus DSM 2379]	gi|118580436	23.5	AFAEERLRAFMR	16.93239	14.96913	14.83666	15.44402	0.88405299	0.876229522	0.912099237
74	surface antigen (D15) [Pelobacter propionicus DSM 2379]	gi|118581666	8.479999542	ILNPAVSTLIR	18.10181	17.57823	16.62998	17.21489	0.971075821	0.918691556	0.951003795
75	sulfite reductase, dissimilatory-type alpha subunit [Candidatus Ruthia magnifica str. Cm (Calyptogena magnif	gi|118602842	5.03000021	AEASA	15.56983	14.44349	14.21125	14.72687	0.927658812	0.912742785	0.945859396
76	ketosteroid isomerase-related protein [[Polyangium] brachysporum]	gi|118764446	20.20999908	SHASKPT	17.30827	16.46078	15.80779	16.54871	0.951035545	0.913308494	0.956115776
77	RecName: Full=UvrABC system protein C	gi|119367679	20.52000046	VSLMAVAKGPDR	17.94448	16.88944	16.72833	17.37899	0.941205318	0.932227069	0.968486688
78	aromatic amino acid aminotransferase [Paracoccus denitrificans PD1222]	gi|119383382	18.80999947	LGATPEQVK	16.93046	16.65604	16.00943	14.31863	0.983791344	0.945599234	0.845731894
79	helix-turn-helix domain-containing protein [Paracoccus denitrificans PD1222]	gi|119384169	9.829999924	VMLAEGEHSVTEIGWR	17.3295	14.73897	14.58832	14.92634	0.850513287	0.841820018	0.861325485
80	sulfoacetaldehyde acetyltransferase [Paracoccus denitrificans PD1222]	gi|119384379	18.37999916	STGRMSMAIAQNGPGVTGFVTPVK	16.71052	16.02837	15.75363	15.66157	0.95917841	0.94273727	0.937228165
81	FGGY-family pentulose kinase [Paracoccus denitrificans PD1222]	gi|119384888	16.69000053	DQYLIGIDVGTGSARAGVFDR	16.9571	14.70802	14.42428	14.96101	0.867366472	0.850633658	0.882285886
82	cytochrome c, class I [Paracoccus denitrificans PD1222]	gi|119385719	3.109999895	SSGEDQS	18.32224	17.42913	17.0849	17.56975	0.951255414	0.932467864	0.95893024
83	hypothetical protein Pden_3616 [Paracoccus denitrificans PD1222]	gi|119386324	11.64999962	AGGMSFGWR	16.12618	15.01988	14.86402	15.40783	0.931397268	0.921732239	0.955454423
84	DXP reductoisomerase [Klebsiella pneumoniae CG43]	gi|1197840	4.630000114	YAVMDDAQSAER	16.11308	14.52328	14.63402	15.45447	0.901334816	0.908207494	0.959125754
85	UDP-N-acetylmuramate:L-alanyl-gamma-D-glutamyl-meso-diaminopimelate ligase [Shewanella amazonensis	gi|119773906	12.67000008	GDQLVVMSNGGFGGIHGKLK	16.74689	13.87144	13.21569	14.51276	0.828299463	0.789142939	0.866594335
86	beta-lactamase class C-like protein [Shewanella amazonensis SB2B]	gi|119774488	21.54999924	LMNSLLTEKANK	0	15.57652	15.84997	16.31759		1.017555269	1.047576095
87	putative acyltransferase family protein [Azoarcus sp. BH72]	gi|119896440	6.769999981	SEPGHR	15.9937	14.61392	14.87783	15.39004	0.913729781	0.930230653	0.962256388
88	nitrogenase iron-molybdenum cofactor biosynthesis protein NifE [Azoarcus sp. BH72]	gi|119896853	22.70999908	ILELMGDKTIMLNSEGAR	17.37149	17.14579	16.62045	15.86579	0.987007447	0.956765942	0.913323497
89	lipid A biosynthesis lauroyl acyltransferase [Azoarcus sp. BH72]	gi|119896881	13.84000015	VALQPLMEAGRARGNMR	17.26905	14.83082	14.86649	15.17679	0.858809257	0.860874802	0.878843364
90	hypothetical protein Ping_0439 [Psychromonas ingrahamii 37]	gi|119944217	9.619999886	GIHNVLTK	16.52456	15.57995	0	15.85235	0.942835997	0	0.959320551
91	CTP synthetase [Psychromonas ingrahamii 37]	gi|119944440	14.68999958	MTARNNFTTGRVYSEVMAK	20.22103	14.11922	14.47899	15.49804	0.698244353	0.716036226	0.766431779
92	crossover junction endodeoxyribonuclease RuvC [Psychromonas ingrahamii 37]	gi|119944487	13.94999981	GDTLAPK	15.67879	14.51413	14.38716	15.11702	0.925717482	0.917619281	0.964170067
93	FM98_ECOLX RecName: Full=Fimbrial protein 987P	gi|120374	5.449999809	SAGTP	16.23319	16.06444	15.18046	15.55998	0.989604631	0.93514953	0.958528792
94	hypothetical protein Maqu_1503 [Marinobacter aquaeolei VT8]	gi|120554423	16.82999992	MGSQQGGCSMR	19.81148	16.99884	18.94806	19.25886	0.858029789	0.956418198	0.972106072
95	hypothetical protein Maqu_3158 [Marinobacter aquaeolei VT8]	gi|120556068	13.56999969	GIGMADTSR	15.02461	14.49157	13.80436	14.1426	0.964522207	0.91878325	0.941295648
96	sodium/proline symporter [Shewanella sp. W3-18-1]	gi|120600778	5.670000076	TTKMFHKAGRVLAEEG	16.39779	15.64441	15.16908	15.72067	0.954056004	0.925068561	0.958706631
97	glycine--tRNA ligase [Desulfovibrio vulgaris DP4]	gi|120602312	11.01000023	LEALAAFSRR	17.29308	15.40182	16.49044	16.63132	0.890634867	0.953586059	0.96173267
98	F0F1 ATP synthase subunit gamma [Acidovorax citrulli AAC00-1]	gi|120609074	11.98999977	SNDAKTAGFIVVTTDK	16.76028	15.66188	15.80638	16.15796	0.934464102	0.943085676	0.964062653
99	diadenosine tetraphosphatase [Acidovorax citrulli AAC00-1]	gi|120609837	29.75	WRAIVNALTR	17.1915	15.10832	15.7691	16.95463	0.878825001	0.917261437	0.986221679
100	propionyl-CoA carboxylase [Acidovorax citrulli AAC00-1]	gi|120610793	10.68000031	ARNPTPMR	15.4106	14.23833	14.54499	14.79382	0.923930931	0.943830221	0.959976899
101	sugar ABC transporter periplasmic sugar-binding protein [Acidovorax citrulli AAC00-1]	gi|120610928	10.28999996	MKRTFLK	16.99135	15.58775	15.49678	16.38147	0.917393262	0.912039361	0.964106442
102	hypothetical protein Aave_2555 [Acidovorax citrulli AAC00-1]	gi|120611224	13.97999954	SLGGGGGRHIRSQGAQR	17.2453	16.25876	16.36945	16.42999	0.942793689	0.949212249	0.952722771
103	glucose-1-phosphate adenylyltransferase [Acidovorax citrulli AAC00-1]	gi|120611645	14.68000031	LEHCIVMERSRIGRGAQVR	16.47127	15.34282	15.2768	15.50634	0.9314898	0.927481609	0.941417389
104	D-serine dehydratase (plasmid) [Polaromonas naphthalenivorans CJ2]	gi|121583347	3.180000067	GALTS	16.03356	15.35005	14.96627	15.2915	0.957370041	0.933433997	0.953718326
105	helicase c2 [Acidovorax sp. JS42]	gi|121594195	13.85999966	DLPLLQQALALPLR	17.11114	15.08018	15.46978	15.92936	0.881307733	0.904076526	0.930935052
106	DNA polymerase I [Polaromonas naphthalenivorans CJ2]	gi|121604384	12.46000004	GMVNMMQKLRKDVR	16.78346	16.31066	15.49027	0	0.971829408	0.922948546	0
107	hypothetical protein Pnap_2413 [Polaromonas naphthalenivorans CJ2]	gi|121605312	13.53999996	VPIRERARCLPHMK	17.07938	15.76952	15.24813	16.98709	0.923307521	0.892780066	0.994596408
108	gamma-glutamyl phosphate reductase [Polaromonas naphthalenivorans CJ2]	gi|121606639	11.85000038	TQKYSPCNATEGLLVAR	16.3237	14.51942	0	14.48638	0.889468687	0	0.887444636
109	molecular chaperone DnaK [Verminephrobacter eiseniae EF01-2]	gi|121607967	61.36999893	LLGEFNLEGIPPAAR	17.89412	13.96392	14.41118	14.74232	0.780363605	0.805358408	0.823863928
110	hypothetical protein Veis_1693 [Verminephrobacter eiseniae EF01-2]	gi|121608659	10.52999973	QKGVDMR	17.76672	0	16.18331	14.75876	0	0.910877753	0.830696943
111	alkaline phosphatase [Verminephrobacter eiseniae EF01-2]	gi|121609923	9.960000038	NLVTPAKAKVAETLDDFR	16.44012	15.8032	14.4493	15.76299	0.96125819	0.878904777	0.958812344
112	integral membrane protein [Neisseria meningitidis FAM18]	gi|121635434	17.12999916	VAGMISASYTGGGVNFAAMSAK	18.24315	0	16.55462	16.24107	0	0.907443068	0.890255795
113	substrate-binding protein [Yersinia enterocolitica subsp. enterocolitica 8081]	gi|123442181	25.02000046	AYAEKIAALDAPLRER	0	17.32665	16.69205	16.1977		0.96337434	0.934843146
114	cysteine synthase [Methylibium petroleiphilum PM1]	gi|124266324	9.25	LEGNNPAGSVKDRPAISMIR	15.25566	0	13.68859	13.04389	0	0.897279436	0.855019711
115	assimilatory nitrite reductase (NAD(P)H) large subunit [Methylibium petroleiphilum PM1]	gi|124267504	18.11000061	EDLPAVWK	16.19157	15.10571	15.15863	15.41258	0.932936707	0.936205075	0.951889162
116	hypothetical protein BMA10229_0272 [Burkholderia mallei NCTC 10229]	gi|124381860	1.519999981	MALGSGS	15.85493	14.86448	14.83029	15.10669	0.937530472	0.935374045	0.952807108
117	hypothetical protein BURPS668_1343 [Burkholderia pseudomallei 668]	gi|126439265	8.420000076	APTMMK	17.1819	16.31846	15.93728	16.42465	0.949747118	0.927562144	0.955927459
118	hypothetical protein BURPS668_A0698 [Burkholderia pseudomallei 668]	gi|126443270	9.479999542	RPPRAAGAK	17.09626	15.91529	15.77814	16.18473	0.930922319	0.922900096	0.946682491
119	DNA polymerase III subunit alpha [Rhodobacter sphaeroides ATCC 17029]	gi|126463991	20.60000038	GVSPIDAVAADAGAMSLR	17.34046	14.36307	15.86684	16.74123	0.828298096	0.915018402	0.965443247
120	Fis family GAF modulated sigma54 specific transcriptional regulator [Rhodobacter sphaeroides ATCC 17029]	gi|126464784	14.64999962	SGLYMGAEWSEAR	15.78408	14.79019	14.49422	14.97396	0.937032124	0.918280951	0.948674867

121	molydopterin dinucleotide-binding region [Shewanella loihica PV-4]	gi|127511179	16.42000008	MWGAHKLGVVAASPVVTPR	17.85321	16.51017	16.7214	17.39987	0.924773192	0.936604678	0.974607368
122	Glu/Leu/Phe/Val dehydrogenase [Shewanella loihica PV-4]	gi|127512484	14.86999989	NALAGLAMGGGKSVIIADPK	16.95135	15.8335	16.04204	16.44899	0.9340554	0.946357665	0.970364602
123	chemotaxis protein cheA [Herminiimonas arsenicoxydans]	gi|134094526	13.47000027	GFGTTISISLPLTLAILDGMSIK	17.31582	16.22297	16.12619	16.47086	0.936887193	0.931298085	0.951203004
124	uridylate kinase [Herminiimonas arsenicoxydans]	gi|134094562	17.60000038	ATADYMGMLATVMNSLALADAMR	15.49409	15.0485	14.83686	15.96662	0.971241293	0.957581891	1.030497435
125	hypothetical protein Bcep1808_6840 [Burkholderia vietnamiensis G4]	gi|134288367	16.88999939	MRPGRELLGLLQQVAEQDSR	17.98701	16.88753	16.64022	17.31423	0.938873665	0.925124298	0.96259634
126	hypothetical protein Bcep1808_6948 [Burkholderia vietnamiensis G4]	gi|134288475	8.390000343	ASISAPVR	16.48424	13.85395	14.05557	13.39375	0.840436077	0.852667154	0.812518503
127	two component, sigma54 specific, Fis family transcriptional regulator [Burkholderia vietnamiensis G4]	gi|134291828	16	MASLGIER	18.14986	16.49929	17.30781	17.349	0.909058803	0.953605703	0.955875142
128	glycosyl transferase family protein [Burkholderia vietnamiensis G4]	gi|134291866	3.200000048	SAGHSK	0	14.59731	15.05413	15.23979		1.031294807	1.044013589
129	hypothetical protein Bcep1808_4022 [Burkholderia vietnamiensis G4]	gi|134292732	13.18999958	ALAAQQVARSDQNGPSK	17.15536	15.80926	16.48002	16.46746	0.921534727	0.960633878	0.959901745
130	molybdate ABC transporter inner membrane subunit [Burkholderia vietnamiensis G4]	gi|134292944	15.02000046	AARSEPT	16.41976	11.02111	15.00844	16.09976	0.671210176	0.914047465	0.980511286
131	RecT protein [Burkholderia vietnamiensis G4]	gi|134293198	19.12000084	EVLLDA	19.36001	18.59718	18.24934	18.59359	0.960597644	0.942630711	0.960412211
132	hypothetical protein Bcep1808_4903 [Burkholderia vietnamiensis G4]	gi|134293589	2.710000038	AGGRY	16.39377	15.48926	15.16291	15.63646	0.944825992	0.924919039	0.953805013
133	3-hydroxy-acyl-CoA dehydrogenase [Burkholderia vietnamiensis G4]	gi|134293749	8.729999542	VVGVGDVPGMVAMR	16.67158	15.36872	15.28724	15.83371	0.921851438	0.916964079	0.949742616
134	sulfate adenylyltransferase subunit 1 [Burkholderia vietnamiensis G4]	gi|134296650	18.59000015	NMVTGASTAHAAIILVDATR	16.02511	15.63288	15.28341	14.87427	0.975524037	0.953716386	0.928185204
135	FAD dependent oxidoreductase [Burkholderia vietnamiensis G4]	gi|134297299	5.170000076	GDASGR	16.10211	14.94493	13.05522	15.71032	0.928134884	0.810776973	0.975668406
136	DNA repair protein RecN [Mesorhizobium loti MAFF303099]	gi|13471539	9.449999809	AAFIVEMK	15.85588	11.54795	14.5361	14.58854	0.728307101	0.916764002	0.920071292
137	trimethylamine methyltransferase MttB [Mesorhizobium loti MAFF303099]	gi|13474510	20.38999939	LPRADMGG	19.01575	16.60013	17.97994	18.47656	0.872967409	0.945528838	0.971645084
138	Protein of unknown function UPF0153 [Magnetospirillum gryphiswaldense MSR-1]	gi|144897704	9.93999958	GMSTSQR	16.05885	15.01006	14.40124	15.06169	0.93469084	0.896779035	0.93790589
139	hypothetical protein MGR_0876 [Magnetospirillum gryphiswaldense MSR-1]	gi|144897800	14.25	ASGEQDK	16.63769	16.14406	15.47931	15.96324	0.970330617	0.930376152	0.959462522
140	Hydrogenase expression/formation protein HypE [Magnetospirillum gryphiswaldense MSR-1]	gi|144898850	13.47000027	TAMGGTR	17.655	16.89674	0	16.84208	0.95705126	0	0.953955253
141	Binding protein component of ABC phosphonate transporter [Magnetospirillum gryphiswaldense MSR-1]	gi|144900933	7.920000076	IKAFFAPDYAGIIEGMR	16.54513	0	14.79728	14.29877	0	0.894358642	0.864228326
142	Ureashort-chain amide or branched-chain amino acid uptake ABC transporter permease protein, possibly fusi	gi|144901056	13.22999954	AAVEAIMNSPGPEAADMVRAALAK	16.9728	13.99493	13.6121	0	0.824550457	0.801994957	0
143	GacA [uncultured Pseudomonas sp.]	gi|144925956	13.68999958	VQIISDKLCLSPKT	17.85593	0	16.42298	16.44235	0	0.919749349	0.920834143
144	4'-phosphopantetheinyl transferase [Aeromonas salmonicida subsp. salmonicida A449]	gi|145298829	6.860000134	RFGLGDHVVAIGSAR	17.43767	14.85155	16.83888	17.02346	0.851693489	0.965661123	0.976246253
145	enoyl-CoA hydratase/isomerase [Polynucleobacter necessarius subsp. asymbioticus QLW-P1DMWA-1]	gi|145589139	14.27999973	ALNALSPDLTR	17.70796	16.12821	16.67242	16.90274	0.910788707	0.941521214	0.954527794
146	hypothetical protein Rsph17025_3044 [Rhodobacter sphaeroides ATCC 17025]	gi|146279074	14.35000038	KPLITDISAAR	17.84062	16.77443	0	16.76051	0.940238063	0	0.939457822
147	hypothetical protein Rsph17025_3575 [Rhodobacter sphaeroides ATCC 17025]	gi|146279591	7.150000095	GTGHIRMKDGK	17.58871	15.22023	15.1255	15.42668	0.865340892	0.859955051	0.877078535
148	S-adenosylmethionine:diacylglycerol 3-amino-3-carboxypropyl transferase-like protein [Rhodobacter sphaeroi	gi|146279632	15.65999985	MTVGPHAALMDATYR	16.99719	15.28801	15.65307	16.4346	0.899443379	0.920921046	0.966901
149	hypothetical protein PST_3344 [Pseudomonas stutzeri A1501]	gi|146283661	12.5	LVEMGFLNFVEER	16.74803	15.58491	0	15.97548	0.930551832	0	0.953872187
150	two-component response regulator CbrB [Pseudomonas mendocina ymp]	gi|146308604	0.460000008	SGSAEK	16.35002	15.30891	15.08331	15.66372	0.936323625	0.922525477	0.958024516
151	outer membrane lipoprotein [Enterobacter sp. 638]	gi|146310398	0.430000007	STATPPK	16.26275	14.14444	14.41444	14.42556	0.869744662	0.88634702	0.887030791
152	hypothetical protein Ent638_2614 [Enterobacter sp. 638]	gi|146312259	15.06999969	ALRIEPEAIR	17.3623	12.51492	14.51	13.32149	0.720810031	0.83571877	0.767265282
153	preprotein translocase subunit YajC [Dichelobacter nodosus VCS1703A]	gi|146328728	17.36000061	EEKIIS	18.49442	17.53322	17.20697	17.36841	0.948027567	0.930387111	0.939116231
154	hypothetical protein DNO_0757 [Dichelobacter nodosus VCS1703A]	gi|146329183	20.26000023	KSQSDPDK	14.42572	14.05094	13.5241	13.93073	0.974020014	0.937499133	0.965686981
155	carboxy-terminal-processing protease [Bradyrhizobium sp. ORS 278]	gi|146337570	4.380000114	MGSTAR	15.95749	14.89874	0	15.11911	0.933651846	0	0.947461662
156	two component sensor histidine kinase osmolarity sensor [Bradyrhizobium sp. ORS 278]	gi|146342542	15.63000011	SKLRPAA	16.6818	16.08952	15.5211	15.83829	0.964495438	0.930421178	0.949435313
157	hypothetical protein COSY_0371 [Candidatus Vesicomyosocius okutanii HA]	gi|148244526	8.319999695	ETLTTVTEARK	16.83109	15.58965	16.00426	16.22005	0.926241259	0.950874839	0.963695756
158	cation-transporting ATPase [Bradyrhizobium sp. BTAi1]	gi|148252261	14.56999969	MPIDTQPAPAASLGLSAAEAARR	18.16643	16.22364	16.14051	16.27622	0.893056038	0.888480015	0.895950388
159	phage related integrase [Bradyrhizobium sp. BTAi1]	gi|148252808	9.779999733	IAASISAAMGDDAGHVAQSRNVAPK	16.98047	14.35724	0	14.33544	0.845514877	0	0.844231049
160	pyrrolidone-carboxylate peptidase [Bradyrhizobium sp. BTAi1]	gi|148254460	12.43999958	QIARKGVIVPGAHPMPFGPHMGR	16.29866	15.34264	15.97079	14.13226	0.941343644	0.979883622	0.867081097
161	short chain dehydrogenase [Bradyrhizobium sp. BTAi1]	gi|148254682	11.51000023	EEIDHAYLGFLNLAQGFGPAMR	17.98535	17.15952	17.02654	17.96022	0.954083184	0.946689389	0.998602752
162	aliphatic nitrilase [Bradyrhizobium sp. BTAi1]	gi|148256113	15.77999973	KTIALIEEAADKGAK	17.64861	0	16.36987	16.20945	0	0.927544436	0.918454768
163	5-methyltetrahydropteroyltriglutamate/homocysteine S-methyltransferase [Acidiphilium cryptum JF-5]	gi|148261198	1.830000043	AEAIPA	18.40256	17.70113	17.13665	17.57136	0.961884107	0.931210114	0.954832371
164	DNA methylase N-4/N-6 domain-containing protein [Geobacter uraniireducens Rf4]	gi|148262672	9.130000114	KEGLEEIYAAFNGFR	18.51595	0	15.43358	15.80699	0	0.83352893	0.853695868
165	aspartate ammonia-lyase [Geobacter uraniireducens Rf4]	gi|148263013	15.39000034	NSVKQFTESCIK	15.47732	12.13093	14.11834	15.18978	0.783787503	0.912195393	0.981421848
166	outer membrane lipoprotein carrier protein LolA [Geobacter uraniireducens Rf4]	gi|148265617	19.61000061	ELQISFDGEGK	17.35879	14.57773	16.33533	16.81885	0.839789525	0.941040821	0.968895297
167	FKBP-type peptidylprolyl isomerase [Orientia tsutsugamushi str. Boryong]	gi|148284322	9.520000458	IPDFSQITLK	16.68929	15.75419	15.62617	15.95858	0.943970055	0.936299267	0.956216831
168	chaperone protein HscA [Orientia tsutsugamushi str. Boryong]	gi|148284613	14.11999989	QAAKIADLDVLR	19.54697	16.90411	17.33983	19.1201	0.86479439	0.887085313	0.978161833
169	hypothetical protein LPC_1158 [Legionella pneumophila str. Corby]	gi|148359263	15.97999954	NLLLGILSMLIKMVK	18.85331	0	15.95898	15.8552	0	0.846481599	0.840976996
170	chemiosmotic efflux system C protein C [Legionella pneumophila str. Corby]	gi|148360314	9.979999542	LDVLKAMAEIR	17.75973	15.13964	16.02642	16.81158	0.852470167	0.902402232	0.946612364
171	methenyltetrahydrofolate cyclohydrolase [Sphingomonas wittichii RW1]	gi|148554979	16.31999969	EGAVIIDVGITR	17.09608	15.60933	15.89627	16.08556	0.91303562	0.929819584	0.940891713
172	hypothetical protein Swit_2351 [Sphingomonas wittichii RW1]	gi|148555266	13.60000038	ELRADIAGELMR	17.06012	14.9124	16.29865	15.9775	0.87410874	0.955365496	0.936540892
173	BadM/Rrf2 family transcriptional regulator [Sphingomonas wittichii RW1]	gi|148555827	9.010000229	MRLSSLADYAVVMLSAAARHGAGER	16.15038	14.97968	15.10804	0	0.927512542	0.935460342	0
174	sugar transferase [Sphingomonas wittichii RW1]	gi|148556341	10.42000008	QIGADPGSVWGR	16.58399	15.1895	15.26251	15.80003	0.91591348	0.920315919	0.952727902
175	type II secretion system protein [Sphingomonas wittichii RW1]	gi|148557757	20.10000038	AGVAASKTRRR	20.06481	17.39754	19.37509	19.40874	0.867067269	0.965625391	0.967302456
176	type 12 methyltransferase [Sphingomonas wittichii RW1]	gi|148557768	15.64999962	RALSVGCGSGGKEMRLLK	16.92488	17.04333	15.83236	16.45768	1.006998573	0.935448878	0.972395668
177	aminodeoxychorismate synthase [Brucella ovis ATCC 25840]	gi|148558359	10.51000023	ISAMKILRRLETGPR	15.8072	12.18006	15.26536	15.4357	0.770538742	0.965721949	0.976498052
178	mannose-1-phosphate guanylyltransferase/mannose-6-phosphate isomerase [Brucella ovis ATCC 25840]	gi|148558633	15.10999966	ALSMIDAMQRYAPQVLAGAR	18.11987	13.65059	0	16.88639	0.753349224	0	0.931926664
179	tRNA uridine 5-carboxymethylaminomethyl modification enzyme GidA [Brucella ovis ATCC 25840]	gi|148559142	29.59000015	MGEKPALGLSKR	19.85399	17.52601	18.71285	19.58286	0.88274498	0.942523392	0.986343803
180	phosphoribosylaminoimidazolesuccinocarboxamide synthase [Psychrobacter sp. PRwf-1]	gi|148653923	9.800000191	IGVPMN	16.75554	15.78963	15.68998	16.21482	0.942352798	0.936405511	0.967728883
181	binding-protein-dependent transport system inner membrane protein (plasmid) [Sinorhizobium medicae WSM	gi|150375742	21.53000069	SQAMY	19.62864	18.55469	18.5901	18.98011	0.945286581	0.947090578	0.966960014

182	DeoR family transcriptional regulator [Sinorhizobium medicae WSM419]	gi|150376764	7.710000038	MNMASR	16.4515	16.94017	15.90238	15.7677	1.029703674	0.966621889	0.958435401
183	beta-ketoadipate pathway transcription regulator [Sinorhizobium medicae WSM419]	gi|150377207	13.77000046	VMSISLMPGSR	0	17.17292	16.87596	17.29894		0.982707658	1.007338298
184	aromatic hydrocarbon degradation membrane protein [Sinorhizobium medicae WSM419]	gi|150396371	12.02999973	RGVLAAVAGMLVASAAQAGGLER	0	16.52612	15.87344	16.28801		0.960506156	0.985591899
185	class II aldolase/adducin family protein [Sinorhizobium medicae WSM419]	gi|150397356	10.68000031	GSVIAAPGDNASSLEEGFLR	14.57142	11.88372	11.78925	12.44712	0.815549891	0.809066652	0.85421462
186	hypothetical protein Smed_2682 [Sinorhizobium medicae WSM419]	gi|150397880	14.31999969	RVGASAIQVAGGGATGSGDTGK	16.48351	16.00335	15.37445	15.66687	0.970870282	0.932717	0.950457154
187	purine nucleoside phosphorylase [Sinorhizobium medicae WSM419]	gi|150398530	10.27999973	GDANAMR	13.75754	16.21564	14.31044	13.64546	1.178672931	1.040188871	0.991853195
188	AprA, partial [Desulfobacterium anilini]	gi|151302311	12.59000015	IMDEYAGGVTAAFKTSK	17.85182	14.91637	0	15.70153	0.835565785	0	0.879547856
189	Zn-finger, prokaryotic DksA/TraR C4 type [Beggiatoa sp. PS]	gi|152066264	12.15999985	KIDASLK	17.78866	16.82157	16.61155	16.92891	0.945634466	0.933828068	0.951668647
190	conserved hypothetical protein, membrane [Beggiatoa sp. PS]	gi|152072015	22.89999962	VYLADK	18.06894	17.0398	16.70632	17.01794	0.943043698	0.924587718	0.941833887
191	Ribosomal protein L5 [Beggiatoa sp. SS]	gi|152145316	10.78999996	MQITLNMGLGEAVGDKK	18.07954	14.8619	14.10038	15.37609	0.822028658	0.779908117	0.850469094
192	putative phosphotransferase system, lactose/cellobiose-specific IIB subunit [Klebsiella pneumoniae subsp. pn	gi|152968925	15.60999966	ADLYVTIK	16.44113	15.05243	15.18509	15.66576	0.915535003	0.923603791	0.952839616
193	hypothetical protein KPN_01118 [Klebsiella pneumoniae subsp. pneumoniae MGH 78578]	gi|152969672	20.19000053	AEKGKGK	18.19195	15.52371	16.20207	16.41856	0.853328533	0.890617553	0.902517872
194	filamentous hemagglutinin outer membrane protein [Actinobacillus succinogenes 130Z]	gi|152978680	8.340000153	VTISGK	15.96076	14.46602	15.25945	15.42969	0.906349071	0.956060363	0.966726522
195	iron-regulated membrane protein [Janthinobacterium sp. Marseille]	gi|152980545	10.64999962	VINMRRIWVK	16.77623	15.40133	15.94484	16.7632	0.918044757	0.950442382	0.999223306
196	ribosomal large subunit pseudouridine synthase B [Janthinobacterium sp. Marseille]	gi|152981088	6.429999828	ARNTPREATEPAVVK	17.20176	15.07019	16.15691	16.24646	0.876084191	0.939259122	0.944464985
197	hypothetical protein mma_1168 [Janthinobacterium sp. Marseille]	gi|152982344	16.40999985	VTLGAQPAEDGTYGDLR	18.95914	19.50957	17.01779	16.81954	1.029032435	0.897603478	0.88714678
198	ferredoxin--nitrite reductase [Sulfurovum sp. NBC37-1]	gi|152991908	15.76000023	NWGIYNIKLMPEK	17.37571	15.20791	14.36376	14.95653	0.87523963	0.826657443	0.860772308
199	hypothetical protein Mmwyl1_0195 [Marinomonas sp. MWYL1]	gi|152994237	9.289999962	RDSNE	17.3686	15.52772	16.19898	17.01724	0.894011031	0.932658936	0.97977039
200	pectinesterase [Marinomonas sp. MWYL1]	gi|152994774	12.46000004	DKTIIAKAIAAGMKGDNGK	15.81633	14.08289	13.79859	0	0.890401882	0.872426789	0
201	hypothetical protein Anae109_0579 [Anaeromyxobacter sp. Fw109-5]	gi|153003452	15.07999992	LRTLMASRRGR	16.07537	15.78147	0	15.35434	0.981717373	0	0.955146911
202	hypothetical protein Anae109_2518 [Anaeromyxobacter sp. Fw109-5]	gi|153005378	10.69999981	EMMSLLEVQGLAPEMEK	18.07385	15.73827	16.45045	16.57172	0.870775734	0.910179624	0.916889318
203	elongation factor G domain-containing protein [Anaeromyxobacter sp. Fw109-5]	gi|153005445	6.230000019	HDATVMNPR	0	14.87059	14.89133	15.63654		1.001394699	1.051507707
204	hypothetical protein Anae109_2814 [Anaeromyxobacter sp. Fw109-5]	gi|153005673	14.97999954	GHLGVIMAPAGVGK	16.38968	16.17597	15.24195	15.94795	0.986960697	0.929972397	0.973048284
205	hypothetical protein Oant_0986 [Ochrobactrum anthropi ATCC 49188]	gi|153008321	8.739999771	IQAAVDLLK	17.62323	15.08354	14.99544	15.72724	0.855889641	0.850890558	0.892415295
206	peptidase M23B [Ochrobactrum anthropi ATCC 49188]	gi|153008395	12.46000004	ARRGVLAEVLAALQR	16.93908	13.82381	14.30605	14.59803	0.816089776	0.844558854	0.861795918
207	major facilitator superfamily transporter [Ochrobactrum anthropi ATCC 49188]	gi|153009261	12.22000027	QSAGLPSR	16.68509	15.72464	15.67793	16.20044	0.942436631	0.939637125	0.970953108
208	delta-aminolevulinic acid dehydratase [Ochrobactrum anthropi ATCC 49188]	gi|153009863	18.13999939	ADWSRRLVQETR	16.33524	14.47431	14.60148	14.64529	0.886078809	0.893863818	0.89654575
209	pilin accessory protein PilO [Yersinia pseudotuberculosis IP 31758]	gi|153948492	16.29000092	ADGATHR	0	16.36649	16.08245	16.56599		0.982645027	1.012189541
210	hypothetical protein JJD26997_1049 [Campylobacter jejuni subsp. doylei 269.97]	gi|153951277	13.65999985	QTGLSYTHLSIKNMKTR	17.30959	16.1673	16.05402	16.85528	0.934008258	0.927463909	0.973753856
211	arylsulfotransferase [Campylobacter curvus 525.92]	gi|154174611	12.51000023	GTFLMRVASGDYK	17.84676	15.77191	15.74329	16.29941	0.883740802	0.88213715	0.913297988
212	methionyl-tRNA formyltransferase [Campylobacter curvus 525.92]	gi|154174950	19.14999962	IFDINGTDLLSTTFADGAKFK	0	15.48242	15.42976	15.83927		0.996598723	1.023048722
213	thioesterase [Xanthobacter autotrophicus Py2]	gi|154246397	22.31999969	AARAAVV	19.32908	18.50272	18.11818	18.60608	0.957247836	0.937353459	0.962595219
214	di-heme cytochrome c peroxidase [Xanthobacter autotrophicus Py2]	gi|154247390	11.97000027	FMAGDR	16.88147	16.04688	15.61621	16.1099	0.95056177	0.925050366	0.954294857
215	membrane-bound proton-translocating pyrophosphatase [Xanthobacter autotrophicus Py2]	gi|154248117	2.099999905	MQEIAAAIAEGAQAYLKR	16.26075	14.8543	15.54985	15.67004	0.91350645	0.956281229	0.963672647
216	Ppx/GppA phosphatase [Parvibaculum lavamentivorans DS-1]	gi|154251620	16.80999947	EGILMSLMDADTARRR	18.1112	0	14.98267	15.35818	0	0.827259928	0.847993507
217	conjugal transfer coupling protein TraG [Parvibaculum lavamentivorans DS-1]	gi|154251650	9.159999847	ISDMVGGK	16.6924	16.71578	15.58281	15.92959	1.001400637	0.933527234	0.954301958
218	hypothetical protein Plav_2168 [Parvibaculum lavamentivorans DS-1]	gi|154252615	24.06999969	FPGVEGSAYLVGGIGINYQQR	18.47764	14.99217	15.1312	15.51558	0.811368227	0.818892456	0.839694896
219	Sel1 domain-containing protein [Parvibaculum lavamentivorans DS-1]	gi|154253420	8.869999886	AGRAE	17.08615	13.10536	14.14437	16.52541	0.76701656	0.827826632	0.967181606
220	PAS/PAC sensor hybrid histidine kinase [Parvibaculum lavamentivorans DS-1]	gi|154253923	10.98999977	TVVVEMLNDLGYR	16.00389	12.21105	14.24771	16.32129	0.763005119	0.890265429	1.019832678
221	hypothetical protein A1S_3805 [Acinetobacter baumannii ATCC 17978]	gi|155030026	16.12999916	MLTPSAAK	17.41292	11.78913	15.24122	16.52924	0.67703349	0.875282262	0.949251475
222	hypothetical protein PA3414 [Pseudomonas aeruginosa PAO1]	gi|15598610	7.46999979	PRQLAGLLCVLLGLAGAPALACPFR	14.51596	14.47723	13.44949	14.14087	0.997331902	0.926531211	0.974160166
223	1-deoxy-D-xylulose 5-phosphate reductoisomerase [Pseudomonas aeruginosa PAO1]	gi|15598846	12.81999969	KISVDSASMMNK	17.07097	15.51155	0	16.36953	0.908650768	0	0.958910361
224	DNA repair protein RecO [Rickettsia prowazekii str. Madrid E]	gi|15604714	7.059999943	ALSYKVGKPYRDK	16.7494	16.60782	15.22813	16.0981	0.99154716	0.909174657	0.961115025
225	hemolysin-like protein [Vibrio cholerae O1 biovar El Tor str. N16961]	gi|15640946	12.90999985	LAVFGGNTQNEQMRAVK	16.32859	13.69718	15.19826	15.84334	0.838846465	0.930776019	0.970282186
226	vitamin B12-transporter ATPase [Vibrio cholerae O1 biovar El Tor str. N16961]	gi|15641258	6.510000229	AGSAK	16.71093	11.54275	15.25105	15.54641	0.690730558	0.912639213	0.930313872
227	leucyl aminopeptidase [Vibrio cholerae O1 biovar El Tor str. N16961]	gi|15642497	5.869999886	SGAAK	16.54262	11.87226	15.26524	15.50153	0.717677127	0.922782485	0.937066196
228	argininosuccinate lyase [Vibrio cholerae O1 biovar El Tor str. N16961]	gi|15642636	6.739999771	VNAAR	17.52302	15.06572	14.63328	15.38251	0.859767323	0.835088929	0.877845828
229	sensor protein RstB [Cronobacter sakazakii ATCC BAA-894]	gi|156934137	14.31000042	ASSFSR	17.78637	16.64822	16.4599	17.22798	0.93600999	0.925422107	0.968605736
230	hypothetical protein ESA_03080 [Cronobacter sakazakii ATCC BAA-894]	gi|156935229	11.31000042	KPINQTAPISPFAK	15.31512	14.07692	14.79939	14.37813	0.919151792	0.966325435	0.938819284
231	Flp pilus assembly protein TadB [Vibrio campbellii ATCC BAA-1116]	gi|156977405	20.64999962	ESISPTLK	17.92681	16.80174	15.95849	17.19832	0.937240926	0.89020244	0.959363099
232	RecA protein [Vibrio aestuarianus]	gi|157100331	13.43000031	AEIEGEMGDSXMGLQAR	16.38616	14.56368	15.86004	13.2946	0.888779311	0.967892417	0.811331026
233	hypothetical protein CKO_02294 [Citrobacter koseri ATCC BAA-895]	gi|157146533	10.72999954	ALGMS	16.87971	16.06398	13.66153	16.14017	0.951673933	0.809346251	0.956187636
234	hypothetical protein CKO_02459 [Citrobacter koseri ATCC BAA-895]	gi|157146693	18.44000053	ESMGGNR	0	15.19635	14.73468	15.41732		0.969619678	1.014540992
235	aldo/keto reductase [Escherichia coli E24377A]	gi|157156985	7.460000038	GGVVIPK	0	16.24352	15.88019	16.34933		0.977632311	1.006513982
236	hypothetical protein Spro_2586 [Serratia proteamaculans 568]	gi|157370826	15.52999973	DAPEVSK	16.94358	16.13857	15.41207	17.14781	0.952488789	0.909611192	1.012053533
237	major facilitator transporter [Serratia proteamaculans 568]	gi|157371815	12.59000015	KPGMSSVLKNK	17.07567	15.81368	15.73391	16.38164	0.926094262	0.921422703	0.959355621
238	branched-chain amino acid transport system II carrier protein [Serratia proteamaculans 568]	gi|157372187	3.25999999	AGTMA	17.08444	16.0887	15.53161	16.10998	0.941716556	0.909108522	0.942962134
239	respiratory nitrate reductase subunit alpha [Shewanella sediminis HAW-EB3]	gi|157375091	13.47999954	IVNVPGSEMTGTR	17.15093	16.06101	16.00698	16.56079	0.936451259	0.933300993	0.96559137
240	dehydrogenase catalytic domain-containing protein [Shewanella sediminis HAW-EB3]	gi|157375314	11.35999966	IAMGKSLDLTAIKGSGPK	16.94879	15.02716	16.58276	16.56462	0.886621405	0.978403768	0.977333485
241	1A family penicillin-binding protein [Rickettsia akari str. Hartford]	gi|157826265	8.710000038	MYKSLFFCLK	15.79125	13.71506	14.2901	15.75996	0.868522758	0.904937861	0.998018523
242	aminopeptidase N [Shewanella pealeana ATCC 700345]	gi|157961759	11.57999992	GNLSKAHHAMASLK	17.28256	15.87578	15.49557	15.69565	0.918601179	0.896601545	0.908178534

243	cytochrome oxidase assembly [Shewanella pealeana ATCC 700345]	gi|157963816	16.82999992	LPMFIAALIIFQAALGMWTVTMK	16.98928	15.01679	16.36162	16.59648	0.883897964	0.963055527	0.976879538
244	carboxylase [Escherichia coli O157:H7 str. EDL933]	gi|15800257	12.89999962	MTIIHPLLASSSAPNYR	18.05083	15.7962	16.57968	17.40308	0.875095494	0.918499593	0.964115224
245	flagellar basal body rod protein FlgG [Escherichia coli O157:H7 str. EDL933]	gi|15801195	2.319999933	DVAIK	16.54485	15.0199	15.19319	15.48559	0.907829325	0.918303279	0.935976452
246	type III effector Hrp-dependent outer protein [Azorhizobium caulinodans ORS 571]	gi|158424399	6.349999905	AFAAMS	17.42345	16.4547	16.20743	16.66156	0.944399645	0.930207852	0.95627215
247	hypothetical protein AZC_2979 [Azorhizobium caulinodans ORS 571]	gi|158424603	18.03000069	GSAGPSRATQAADLAAAVDHKVR	17.44435	0	15.20945	15.68141	0	0.87188402	0.898939198
248	methyltransferase [Azorhizobium caulinodans ORS 571]	gi|158426043	0.569999993	PGGGR	15.79376	15.08689	14.60925	14.93087	0.955243717	0.925001393	0.945365132
249	hypothetical protein Dole_0430 [Desulfococcus oleovorans Hxd3]	gi|158520447	13.90999985	WLVQNTGSRGSSGVGTGSQAMDILK	16.62174	0	15.14366	15.02492	0	0.911075495	0.903931839
250	H+transporting two-sector ATPase B/B' subunit [Desulfococcus oleovorans Hxd3]	gi|158520820	13.85000038	EAEDLAAGIMEHILDRSVTAS	16.19749	13.94532	15.51704	0	0.860955617	0.957990405	0
251	hypothetical protein Dole_1214 [Desulfococcus oleovorans Hxd3]	gi|158521227	17.18000031	GYEGMDEITR	17.99309	15.75142	17.21085	17.11901	0.875414951	0.956525533	0.951421351
252	hypothetical protein SMc01150 [Sinorhizobium meliloti 1021]	gi|15964123	13.22999954	GGLLKQLGR	17.55914	16.37565	15.51911	16.05897	0.932599774	0.883819481	0.914564722
253	hydrolase [Sinorhizobium meliloti 1021]	gi|15966370	15.51000023	TADLPFGK	18.67931	17.32181	17.87467	17.87179	0.927326009	0.956923462	0.956769281
254	hypothetical protein SMc00718 [Sinorhizobium meliloti 1021]	gi|15966407	8.890000343	TAGIDLIPPERASRSSAAQPRR	17.26571	15.91885	16.04293	16.33834	0.921992203	0.929178702	0.946288337
255	Tfp pilus assembly protein FimT-like protein [Delftia acidovorans SPH-1]	gi|160898322	15.93000031	SEAVYKR	16.03547	15.06721	14.74906	15.17601	0.93961761	0.919777219	0.946402569
256	hypothetical protein Daci_2988 [Delftia acidovorans SPH-1]	gi|160898429	6.699999809	SAKPV	16.0496	14.97854	14.8728	15.24601	0.933265627	0.9266773	0.949930839
257	response regulator receiver modulated diguanylate cyclase/phosphodiesterase [Delftia acidovorans SPH-1]	gi|160898477	6.760000229	RLASALPAGVLLAR	17.6613	16.29994	16.15104	16.64151	0.922918471	0.914487608	0.942258497
258	hypothetical protein Daci_3897 [Delftia acidovorans SPH-1]	gi|160899331	11.21000004	QMPAAPRALRLLMAGTAAK	16.19658	14.00253	15.80195	16.83937	0.864536217	0.97563498	1.039686773
259	hypothetical protein CC_2550 [Caulobacter crescentus CB15]	gi|16126789	17.23999977	DSEIMQINGRSSRK	17.21087	14.59665	0	14.25811	0.848106458	0	0.828436331
260	hypothetical protein CC_3114 [Caulobacter crescentus CB15]	gi|16127344	13	MILPAAMPSRPGRERSRR	16.0851	16.47619	0	15.60537	1.024313806	0	0.970175504
261	alkaline phosphatase isozyme conversion aminopeptidase [Salmonella enterica subsp. arizonae serovar 62:z	gi|161502004	0.330000013	AEKTP	16.29714	15.61806	15.44112	15.49013	0.958331339	0.947474219	0.950481496
262	hypothetical protein SPAB_03428 [Salmonella enterica subsp. enterica serovar Paratyphi B str. SPB7]	gi|161615644	9.319999695	AFRTSSDDSGNHR	16.65715	14.93504	14.92778	15.31603	0.896614367	0.896178518	0.919486827
263	insertion element IS2 transposase InsD [Shigella boydii Sb227]	gi|161984955	6.989999771	ANEARQFARMLGLEPK	17.27439	13.3981	0	13.53827	0.775604812	0	0.783719136
264	transglutaminase-like protein [Gluconacetobacter diazotrophicus PAl 5]	gi|162146008	22.11000061	AFEMPPHPQMAAAQMLLMR	14.90393	13.33639	15.19286	14.42139	0.894823714	1.019386162	0.967623305
265	hypothetical protein GDI_1748 [Gluconacetobacter diazotrophicus PAl 5]	gi|162147532	12.22000027	MRRLSLVVGLCLLAGCASQPSRK	16.66866	14.91474	14.36474	14.90682	0.894777385	0.861781331	0.894302241
266	hypothetical protein GDI_2753 [Gluconacetobacter diazotrophicus PAl 5]	gi|162148530	13.05000019	DGGSIRYARNLIVTLR	17.43847	15.11808	14.53898	0	0.866938441	0.833730253	0
267	TetR family transcriptional regulator [Sorangium cellulosum So ce56]	gi|162450910	16.84000015	GGPGRR	16.79866	15.59745	15.63503	16.14985	0.928493701	0.930730784	0.961377277
268	hypothetical protein sce4334 [Sorangium cellulosum So ce56]	gi|162452610	14.59000015	YVVLPGVELAPAVEQK	17.65063	15.74458	16.36089	16.39405	0.892012353	0.92692952	0.928808207
269	hypothetical protein sce5020 [Sorangium cellulosum So ce56]	gi|162453296	16.65999985	AMGDQALLDALDHAPPGMMDPK	17.52008	16.28985	15.84074	16.26027	0.929781713	0.904147698	0.928093365
270	hypothetical protein sce6639 [Sorangium cellulosum So ce56]	gi|162454921	18.94000053	AVEGGHRR	16.45253	15.8432	15.81165	0	0.962964359	0.96104672	0
271	protein kinase [Sorangium cellulosum So ce56]	gi|162457435	18.28000069	KSVLPPPMEE	17.8998	16.87743	16.75266	17.05863	0.942883719	0.93591325	0.953006738
272	asparagine synthetase (glutamine amidotransferase) protein [Sinorhizobium meliloti 1021]	gi|16264211	8.329999924	GPDASGVVVRGNVGLGHRR	15.64189	14.48137	14.52472	15.15304	0.92580692	0.928578324	0.968747383
273	hypothetical protein Mext_2864 [Methylobacterium extorquens PA1]	gi|163852282	14.11999989	SGAAAAKAAASASEAPLPANLR	18.0053	14.7347	14.8564	15.31214	0.818353485	0.825112606	0.850424042
274	transposase IS3/IS911 [Bordetella petrii DSM 12804]	gi|163855503	19.07999992	DQRLGREAPASMTRHR	17.33442	15.58859	15.54418	17.28124	0.899285352	0.896723398	0.996932115
275	phage-related integrase [Bordetella petrii DSM 12804]	gi|163858864	12.55000019	AEAEKRASGIDPK	17.7865	15.93219	16.06136	17.01342	0.895746212	0.903008461	0.956535575
276	binding-protein-dependent transport system inner membrane protein [Pseudomonas putida GB-1]	gi|167033663	18.42000008	SVKTAYGLVQAKRWPR	19.54791	16.32708	17.72553	17.93662	0.835234048	0.906773665	0.917572262
277	tetrathionate reductase subunit B [Salmonella enterica subsp. enterica serovar Typhi str. CT18]	gi|16760525	13.52999973	IATMLHQHRDAIK	17.90592	14.59665	14.55313	14.95629	0.815185704	0.812755223	0.835270681
278	hypothetical protein Fphi_1103 [Francisella philomiragia subsp. philomiragia ATCC 25017]	gi|167627328	11.09000015	MSKTIENIAEFAQR	17.20908	15.0916	16.62382	16.10886	0.876955654	0.965991209	0.936067471
279	amidohydrolase 2 [Caulobacter sp. K31]	gi|167645895	11.63000011	LSELNGVMAPDAK	15.91992	15.35349	14.74967	14.8334	0.964420047	0.926491465	0.931750913
280	bacteriophage protein [Acinetobacter baumannii SDF]	gi|169633974	13.17000008	RTMTAASIGADGK	18.13613	15.32899	16.25567	16.66895	0.845218357	0.896314165	0.919101815
281	putative ThiJ/PfpI family protein [Acinetobacter baumannii AYE]	gi|169786903	29.40999985	NNMKILIVLTSHDQLGNTGK	19.3091	16.21633	16.40193	16.3305	0.839828371	0.849440419	0.845741127
282	dissimilatory sulfite reductase alpha subunit [uncultured Desulfobacteraceae bacterium]	gi|169907871	12.35000038	LAIDTSNCTRCMHCINTMPR	17.29463	17.56809	16.52703	16.99795	1.015811844	0.955616281	0.982845542
283	TonB-dependent siderophore receptor [Yersinia pseudotuberculosis YPIII]	gi|170023064	13.44999981	MNKINMFMLSTFMR	16.82862	15.90106	15.67658	0	0.944881993	0.931542812	0
284	mgtC family protein [Escherichia coli SMS-3-5]	gi|170679634	13.5	MNTLSFLLFSEEESLFITLGK	15.96212	13.90119	13.66665	14.15374	0.870886198	0.856192661	0.886708031
285	leucyl aminopeptidase [Haemophilus somnus 2336]	gi|170718598	7.889999866	EVAENPHLMGK	16.53673	15.45347	15.26366	15.7427	0.9344937	0.923015614	0.951983857
286	hypothetical protein PputW619_2193 [Pseudomonas putida W619]	gi|170721374	14.46000004	RLALALLAGTLGAVLMTVALYGVR	17.90252	14.86014	14.72758	14.68212	0.830058562	0.822654017	0.82011471
287	outer membrane adhesin-like protein [Shewanella woodyi ATCC 51908]	gi|170724858	13.47000027	ESLVTTQNGQVR	16.23579	14.9287	14.9386	15.21683	0.919493292	0.920103056	0.937239888
288	EAL domain-containing protein [Shewanella woodyi ATCC 51908]	gi|170725185	11.96000004	FPKPPNLL	15.86143	14.20636	14.45117	14.7565	0.895654427	0.911088723	0.930338563
289	hypothetical protein Swoo_1740 [Shewanella woodyi ATCC 51908]	gi|170726094	13.42000008	GEYGSTAGTK	14.63607	13.33524	13.48796	14.03367	0.911121633	0.921556128	0.95884141
290	ABC transporter-like protein [Shewanella woodyi ATCC 51908]	gi|170728558	23.47999954	GTANMMPNELSGGMQRR	19.31039	17.50932	0	18.49982	0.906730522	0	0.958024152
291	peptidoglycan-associated outer membrane lipoprotein [Xylella fastidiosa M12]	gi|170730067	6.5	TGGSK	16.16074	15.85428	15.08253	15.44646	0.981036759	0.933282139	0.955801529
292	diguanylate cyclase/phosphodiesterase [Methylobacterium sp. 4-46]	gi|170738409	12.53999996	WDNLGSSESEFAAIIAAR	18.21205	16.57032	16.84472	16.9984	0.909854739	0.924921686	0.933360056
293	hypothetical protein M446_0727 [Methylobacterium sp. 4-46]	gi|170739065	16.57999992	RAAGPAAR	17.19625	15.96073	16.13785	16.45999	0.928151777	0.938451697	0.957184851
294	patatin [Methylobacterium sp. 4-46]	gi|170739649	7.230000019	NAMNPIHAPAADR	16.76557	15.24348	15.83425	0	0.909213346	0.944450442	0
295	peptidyl-tRNA hydrolase [Methylobacterium sp. 4-46]	gi|170740630	24.98999977	RKFQGEASEAVIGTER	0	17.26768	17.22266	17.86636		0.997392817	1.034670552
296	hypothetical protein M446_3221 [Methylobacterium sp. 4-46]	gi|170741406	11.55000019	GSSDGDGR	17.09507	16.27677	15.93164	16.26565	0.952132398	0.931943537	0.951481918
297	protochlorophyllide reductase iron-sulfur ATP-binding protein [Methylobacterium sp. 4-46]	gi|170741880	23.34000015	LAPTVIDALEAVK	18.03437	15.41417	17.10149	17.27853	0.854710755	0.948272105	0.958088916
298	CarD family transcriptional regulator [Methylobacterium sp. 4-46]	gi|170742914	23.18000031	KALDVLTGRAR	0	16.92324	16.47721	16.53525		0.973643936	0.977073539
299	hypothetical protein Mrad2831_0118 [Methylobacterium radiotolerans JCM 2831]	gi|170746568	9.109999657	NLTLRLGRVLCAAALAASLGSAAR	16.48092	14.59356	14.30977	0	0.885482121	0.868262815	0
300	hypothetical protein Mrad2831_0376 [Methylobacterium radiotolerans JCM 2831]	gi|170746821	13.38000011	RMHDLMSSAR	17.71352	15.13564	15.20482	15.45015	0.854468225	0.858373717	0.87222359
301	peptidase M16 domain-containing protein [Methylobacterium radiotolerans JCM 2831]	gi|170748220	11.27999973	VAAVGAIGAEQLAEGLNR	16.95411	15.21953	15.84365	17.37307	0.897689705	0.934502018	1.024711412
302	acyl-CoA dehydrogenase domain-containing protein [Methylobacterium radiotolerans JCM 2831]	gi|170749664	6.670000076	IAAPGTIR	0	16.08543	16.27103	16.69698		1.011538392	1.038018878
303	hypothetical protein Mrad2831_5065 [Methylobacterium radiotolerans JCM 2831]	gi|170751446	10.93000031	MVAGLGMLAAFALVSMLLSRR	16.9995	14.45359	14.27101	14.88094	0.850236183	0.839495868	0.875375158

304	TolC family type I secretion outer membrane protein [Leptothrix cholodnii SP-6]	gi|171059120	2.460000038	GVSSNAR	17.88286	17.29976	16.65643	17.13734	0.967393359	0.931418688	0.958310919
305	TRAP transporter solute receptor, TAXI family [Polynucleobacter necessarius subsp. necessarius STIR1]	gi|171463981	2.349999905	LNLVH	18.96321	17.19209	16.80396	17.33186	0.90660231	0.886134784	0.913972898
306	glycosyltransferase 36 [Burkholderia ambifaria MC40-6]	gi|172062920	14.14000034	VVAAAAAQGANRTSAAGAAAGR	16.58747	0	15.13458	15.90257	0	0.912410392	0.958709797
307	hypothetical protein RSc2471 [Ralstonia solanacearum GMI1000]	gi|17547190	10.60999966	LAIANHLAIATSTLR	16.47479	15.52569	14.87876	13.87797	0.942390768	0.903122893	0.84237614
308	hypothetical protein Bphy_6126 [Burkholderia phymatum STM815]	gi|186470911	10.44999981	GTCVDAK	17.07419	16.16082	15.81814	16.25029	0.946505808	0.926435749	0.951745881
309	hypothetical protein Bphy_7216 [Burkholderia phymatum STM815]	gi|186471944	16.39999962	NTAAPQP	17.28609	16.56882	16.1236	16.46458	0.958505943	0.932749974	0.952475661
310	extracytoplasmic-function sigma-70 factor [Burkholderia phymatum STM815]	gi|186472874	17	MAESVGSFAARVER	17.77472	14.62162	14.80536	15.24426	0.822607614	0.832944766	0.857637139
311	ATP-dependent DNA ligase [Burkholderia phymatum STM815]	gi|186473494	22.45999908	GASPETLR	17.27757	17.61496	14.94303	16.24157	1.01952763	0.864880304	0.940037864
312	integral membrane sensor signal transduction histidine kinase [Burkholderia phymatum STM815]	gi|186475206	6.699999809	ARDLP	18.68276	18.50249	17.42224	17.65369	0.990350997	0.932530311	0.944918738
313	Sua5/YciO/YrdC/YwlC family protein [Burkholderia phymatum STM815]	gi|186475233	16.39999962	DLYGLLRALDR	19.26601	0	17.95175	17.66405	0	0.931783488	0.916850453
314	hypothetical protein Bphy_1894 [Burkholderia phymatum STM815]	gi|186476649	8.449999809	GDFRDPAVVLEEKQSR	17.89209	0	16.67317	16.34597	0	0.931873806	0.913586395
315	hypothetical protein Bphy_2396 [Burkholderia phymatum STM815]	gi|186477147	5.659999847	QASAAYRTPDER	15.94291	14.71657	14.8015	0	0.923079287	0.92840642	0
316	dehydrogenase [Bordetella avium 197N]	gi|187476626	23.47999954	VKGVSGLR	17.46973	16.67336	15.66891	16.64354	0.954414293	0.896917697	0.95270734
317	general secretion pathway protein E [Bordetella avium 197N]	gi|187476848	18.95000076	VDGTLRDVVTPR	15.07396	15.50658	16.02676	12.8218	1.028699824	1.063208341	0.850592678
318	DNA polymerase III subunit delta [Bordetella avium 197N]	gi|187478982	8.770000458	QAGYTDRTTLVMDAR	16.07433	13.36127	13.48364	13.98221	0.831217849	0.838830608	0.869847141
319	capsular polysaccharide biosynthesis protein [Bordetella avium 197N]	gi|187479123	14.22999954	QVRGELSIRLGGLPR	15.55576	15.37194	15.11912	11.54045	0.988183155	0.971930655	0.741876321
320	ferrochelatase, partial [Bordetella avium 197N]	gi|187479193	19.75	GLDVPVALGMR	19.41295	14.5774	18.38639	18.00538	0.750911119	0.947119835	0.927493245
321	hypothetical protein Bphyt_7354 (plasmid) [Burkholderia phytofirmans PsJN]	gi|187921979	5.690000057	RGMSM	17.3808	16.59363	16.1486	16.61399	0.95471037	0.92910568	0.955881778
322	aldo/keto reductase [Burkholderia phytofirmans PsJN]	gi|187923424	6.920000076	NPKAV	16.65582	15.46316	15.64691	16.12042	0.928393799	0.939425978	0.96785508
323	glyoxalase/bleomycin resistance protein/dioxygenase [Burkholderia phytofirmans PsJN]	gi|187923483	22.60000038	KSDPSAQT	17.19077	16.90854	16.62398	11.68429	0.983582469	0.9670294	0.679683923
324	transmembrane anti-sigma factor [Burkholderia phytofirmans PsJN]	gi|187923775	5.78000021	TEAGS	15.91066	14.7221	15.22083	14.89493	0.925297882	0.956643533	0.936160411
325	ribonuclease BN [Ralstonia pickettii 12J]	gi|187925962	14.22000027	DVALGAVVTALLFTLGK	17.14729	16.88747	15.96284	16.39163	0.984847751	0.930924945	0.955931229
326	hypothetical protein HPSH_03445 [Helicobacter pylori Shi470]	gi|188527471	7.960000038	VPLPAASMMTENIK	16.20336	15.02464	14.74513	15.26392	0.927254594	0.910004468	0.942021902
327	outer membrane protein [Helicobacter pylori Shi470]	gi|188527964	22.51000023	TSAQAINQAVNNLNERAKK	19.48078	18.05938	18.34828	18.36505	0.927035776	0.941865777	0.942726626
328	hypothetical protein ETA_00040 [Erwinia tasmaniensis Et1/99]	gi|188532169	17.05999947	LAMSGQLAPVLADDDEAAAGR	18.30705	15.05362	15.08756	15.20792	0.822285404	0.824139334	0.830713851
329	hypothetical protein ETA_24120 [Erwinia tasmaniensis Et1/99]	gi|188534539	8.430000305	FVESPIDSKK	16.50435	14.92064	12.53215	15.14559	0.904042874	0.759324057	0.917672614
330	Cellulose synthase operon protein C [Erwinia tasmaniensis Et1/99]	gi|188535499	12.43999958	IPPRDRNTDMRDLMR	16.34984	14.69087	15.48423	15.23388	0.898532952	0.947056974	0.931744898
331	hypothetical protein PXO_00498 [Xanthomonas oryzae pv. oryzae PXO99A]	gi|188576065	17.57999992	SQPLTSGTPMLYALR	17.94102	17.5613	16.98787	16.40796	0.978835094	0.946873143	0.914550009
332	signal recognition particle protein [Xanthomonas oryzae pv. oryzae PXO99A]	gi|188578077	7.829999924	MMGKLAGGGMK	15.9564	14.7705	14.78282	15.25449	0.925678725	0.926450829	0.956010754
333	hypothetical protein Mpop_0815 [Methylobacterium populi BJ001]	gi|188580083	7.349999905	FLDMEGPVPAPAR	17.83695	16.12764	16.57049	17.13582	0.904170276	0.928997951	0.960692271
334	hypothetical protein Mpop_2092 [Methylobacterium populi BJ001]	gi|188581344	8.470000267	ISRWLKGHEVGGK	17.47568	15.88163	15.97939	0	0.908784665	0.914378725	0
335	ferritin Dps family protein [Methylobacterium populi BJ001]	gi|188583374	19.61000061	YADTIAERLLAVGVSADGR	17.59952	16.62534	15.73876	15.30367	0.944647354	0.894272117	0.86955042
336	hypothetical protein RALTA_A0496 [Cupriavidus taiwanensis LMG 19424]	gi|188591284	18.20999908	MNDTPAPGSAEAR	0	14.70966	14.53235	15.02563		0.987946016	1.021480442
337	transposase, IS21 family (plasmid) [Cupriavidus taiwanensis LMG 19424]	gi|188591778	3.859999895	AAVEP	17.88262	0	15.30813	15.89204	0	0.856033959	0.888686333
338	aminopeptidase B [Xanthomonas campestris pv. campestris str. B100]	gi|188990106	12.56999969	IAWSGSGTLPNK	17.3449	15.3597	15.61205	15.91082	0.885545607	0.900094552	0.917319789
339	hypothetical protein Glov_0746 [Geobacter lovleyi SZ]	gi|189423815	9.010000229	CSPMGVTASFLNINK	18.0034	0	14.91991	15.94118	0	0.828727352	0.885453859
340	5'-nucleotidase domain-containing protein [Geobacter lovleyi SZ]	gi|189425434	13.03999996	AGVVSGYSAK	17.64246	16.11627	16.84664	16.97918	0.913493356	0.954891778	0.962404336
341	2-oxoglutarate dehydrogenase, E2 component, dihydrolipoamide succinyltransferase [Wolbachia endosymbio	gi|190570534	20.39999962	KIMEENAISAENVKGTGMGGR	17.17716	15.60045	16.06568	18.13195	0.908208924	0.935293145	1.055584858
342	transmembrane anchor protein [Stenotrophomonas maltophilia K279a]	gi|190572596	10	AKTYTVTVRAEINTTLLQTK	0	16.36759	15.82415	16.29545		0.9667978	0.995592509
343	D-lactate dehydrogenase [Stenotrophomonas maltophilia K279a]	gi|190574812	8.010000229	GWGGCDCG	16.84534	12.34655	13.97386	15.75695	0.732935637	0.829538614	0.935389253
344	colony opacity-associated adhesin [Neisseria meningitidis]	gi|190692395	13.94999981	GAKVGGK	16.56164	15.8277	15.37452	0	0.95568434	0.928321108	0
345	hypothetical protein RHECIAT_CH0002438 [Rhizobium etli CIAT 652]	gi|190892027	13.93999958	SNSMMEAVASAEARVAGAFADK	16.58076	14.08114	13.96472	0	0.849245752	0.842224361	0
346	amino acid ABC transporter substrate-binding protein [Rhizobium etli CIAT 652]	gi|190892983	18.54999924	DSGIKEMKDLVGKK	16.61957	14.63756	15.28022	18.30613	0.880742402	0.919411272	1.101480363
347	sensory box protein [Cellvibrio japonicus Ueda107]	gi|192359680	10.93000031	LIIEITETAMLEDLER	0	15.22448	14.92241	14.27485		0.980158928	0.937624799
348	2-isopropylmalate synthase [Cellvibrio japonicus Ueda107]	gi|192361320	5.210000038	SGAVS	16.78068	15.02444	14.72309	15.27189	0.895341548	0.877383396	0.910087672
349	Oxidoreductase (plasmid) [Sinorhizobium meliloti 1021]	gi|193782682	13.43000031	SGATP	16.6405	15.84859	15.43998	15.86046	0.952410685	0.927855533	0.953124005
350	HrpA protein [Neisseria gonorrhoeae NCCP11945]	gi|194099492	16.02000046	TQIRATKEAGYEQIHR	0	16.47677	16.70482	17.45816		1.013840698	1.059562038
351	hypothetical protein RALTA_A2597 [Cupriavidus taiwanensis LMG 19424]	gi|194290682	12.09000015	GPDMFDVEAFPEATYK	16.0874	14.87378	15.07748	0	0.924560836	0.93722292	0
352	hypothetical protein RALTA_B0993 [Cupriavidus taiwanensis LMG 19424]	gi|194291754	16.85000038	DPKSQWLYTVNKDVMRDK	18.14239	16.24761	16.52158	16.97064	0.895560618	0.910661715	0.935413691
353	phage protein [Salmonella enterica subsp. enterica serovar Heidelberg str. SL476]	gi|194449502	20.12000084	EVLISTVLANMFVATASGSMLR	18.94045	15.60097	16.30643	16.35282	0.823685287	0.860931498	0.863380754
354	threonine dehydratase [Phenylobacterium zucineum HLK1]	gi|197103871	12.52999973	MSVTPPGVTLADIEAAAARLK	17.19039	15.34604	14.00081	15.68545	0.892710404	0.814455635	0.912454575
355	hypothetical protein PHZ_c1822 [Phenylobacterium zucineum HLK1]	gi|197105285	11.07999992	DTTIGPPQPLGTRGAVMADTVGEPQ	16.14307	0	14.2907	14.60218	0	0.885252929	0.904547896
356	dienelactone hydrolase [Phenylobacterium zucineum HLK1]	gi|197106897	13.31000042	NAAGIDAAPAGVACAR	17.73763	16.53701	16.96696	17.34955	0.932312265	0.956551693	0.978121091
357	succinyl:acetate CoA transferase [Geobacter bemidjiensis Bem]	gi|197117187	16.18000031	QPSIGDSAAV	16.43963	16.59895	15.22233	15.73518	1.009691216	0.925953321	0.957149279
358	electron transfer flavoprotein subunit beta [Geobacter bemidjiensis Bem]	gi|197117887	11.64000034	DRFGFKVVAISMGPPNAEATLR	17.67676	15.37486	0	16.76755	0.869778172	0	0.948564669
359	late embryogenesis abundant protein 2 [Anaeromyxobacter sp. K]	gi|197120507	8.489999771	AASELMRGAPVDVGMR	16.24927	14.45507	0	14.80434	0.889582732	0	0.911077236
360	radical SAM protein [Anaeromyxobacter sp. K]	gi|197121329	9.25	AATLGIR	16.64318	14.57635	14.8563	14.7569	0.875815199	0.892635903	0.886663486
361	alkaline phosphatase precursor [Halomonas sp. #593]	gi|197267661	12.47999954	TAVGSLVGGMLLASVAVPASAAEVK	17.23616	15.21074	15.46602	15.4618	0.882490067	0.897300791	0.897055957
362	nucleoside permease [Proteus mirabilis HI4320]	gi|197285678	16.86000061	GLNEMQGNVVSR	20.75483	17.96177	19.39287	19.45306	0.865426024	0.934378648	0.937278696
363	hypothetical protein VFMJ11_0311 [Vibrio fischeri MJ11]	gi|197334110	5.110000134	SISTMA	17.04614	14.88245	14.13258	13.91704	0.873068624	0.82907802	0.816433515
364	hypothetical protein VFMJ11_B0092 [Vibrio fischeri MJ11]	gi|197336641	8.050000191	WVPTDINLNK	16.3552	15.56389	15.24099	15.09809	0.951617223	0.931874266	0.923136984

365	sialate-O-acetyltranferase [Vibrio fischeri MJ11]	gi|197336766	16.75	MPSSLKIEENVAFR	16.05927	14.64934	15.10567	15.3359	0.912204602	0.940619966	0.954956234
366	lipoprotein, putative [Vibrio fischeri MJ11]	gi|197337654	11.60999966	INGITSLIAGHMNNGMTAKK	17.58985	16.37471	16.53368	16.68019	0.930918115	0.939955713	0.948284948
367	prevent-host-death family protein [Acidithiobacillus ferrooxidans ATCC 53993]	gi|198282717	11.85000038	NGRPAAKLVPMETSPGGKR	16.572	14.32747	15.73158	15.44339	0.864558895	0.949286749	0.931896573
368	TrmH family RNA methyltransferase [Acidithiobacillus ferrooxidans ATCC 53993]	gi|198283233	19.86000061	TLSSLQEAGFWLVGMAGEGSR	17.53177	16.03268	15.05343	15.20966	0.914492946	0.858637205	0.867548456
369	hypothetical protein Lferr_1309 [Acidithiobacillus ferrooxidans ATCC 53993]	gi|198283430	9.170000076	IKVLIISRR	17.46886	14.98656	15.2075	15.722	0.857901431	0.87054908	0.900001488
370	carboxysome structural protein CsoS2 [Acidithiobacillus ferrooxidans ATCC 53993]	gi|198283506	8.720000267	ISHNSGQPDALAGLSGR	17.28852	15.60547	17.06946	17.05	0.902649272	0.987329164	0.986203562
371	putative type IV pilus assembly protein [Burkholderia cenocepacia J2315]	gi|206558682	12.03999996	TMSGGGA	15.90234	15.31701	15.08704	13.05794	0.963192209	0.948730816	0.821133242
372	hypothetical protein BCAL2965b [Burkholderia cenocepacia J2315]	gi|206561304	4.71999979	GEVLHG	16.40931	15.74135	15.20046	15.89385	0.95929384	0.926331455	0.968587345
373	succinyl-coa synthetase beta chain (scs-beta) protein [Ralstonia solanacearum IPO1609]	gi|207742410	48.81000137	AAETLGGPVWVVK	16.45599	17.76756	13.69967	15.82538	1.079701677	0.832503544	0.961679
374	betaine aldehyde dehydrogenase (badh) protein [Ralstonia solanacearum IPO1609]	gi|207743192	12.14999962	TGQVDINGGPFNMQAPFGGYK	16.22028	0	16.00095	15.64262	0	0.986478039	0.964386558
375	fructose-1,6-bisphosphatase protein [Ralstonia solanacearum IPO1609]	gi|207743755	8.25	IPADTK	17.16431	16.23094	0	16.14225	0.945621467	0	0.94045435
376	PROBABLE TRANSKETOLASE BETA SUBUNIT PROTEIN [Mesorhizobium loti R7A]	gi|20803910	11.02000046	MVVVAENHTMIGGLGEGVAGLLMR	16.62195	0	15.36891	15.39676	0	0.924615343	0.926290838
377	adenylate/guanylate cyclase [Rhizobium leguminosarum bv. trifolii WSM2304]	gi|209546340	14.27000046	AGLYGAFSAALLIVVAARARRR	18.22737	17.31056	17.04374	16.97554	0.949701465	0.93506304	0.931321414
378	hypothetical protein Rleg2_0859 [Rhizobium leguminosarum bv. trifolii WSM2304]	gi|209548462	13.72999954	KLADMAAMEG	16.97959	14.94599	15.65114	16.44955	0.880232679	0.921761951	0.968783699
379	phospholipid N-methyltransferase protein [Rhizobium leguminosarum bv. trifolii WSM2304]	gi|209551894	8.510000229	RFDEEIRFFRGMMQGPK	17.50133	15.7753	16.00803	16.3124	0.90137721	0.914675056	0.932066306
380	acriflavin resistance protein [Oligotropha carboxidovorans OM5]	gi|209885444	12.28999996	SVLMMVLKAGSTSTLDIIDGIK	16.74514	17.3045	14.34066	14.41439	1.033404319	0.856407292	0.86081036
381	sensor signal transduction histidine kinase [Oligotropha carboxidovorans OM5]	gi|209885662	8.880000114	ATRQIVLNLLSNAIK	17.43374	16.29029	16.53658	16.93347	0.934411664	0.948538868	0.971304493
382	MobD-like protein [Escherichia coli]	gi|209947792	9.430000305	ENAALSER	16.17235	0	14.99913	15.76194	0	0.927455194	0.974622736
383	hypothetical protein RC1_1185 [Rhodospirillum centenum SW]	gi|209964497	16.71999931	MSDMSRPGFLLPLAR	18.96729	15.30969	16.22403	16.444	0.807162752	0.855368901	0.866966235
384	hypothetical protein XAC0137 [Xanthomonas axonopodis pv. citri str. 306]	gi|21240911	15.06000042	RRGNGASQATTRAR	16.44288	15.15546	14.9668	15.49223	0.921703497	0.910229838	0.942184702
385	hypothetical protein swp_2793 [Shewanella piezotolerans WP3]	gi|212635581	14.84000015	GMALDIAVPGVKLAEVR	15.78301	14.51098	14.18574	0	0.919405107	0.898798138	0
386	hypothetical protein Tmz1t_0740 [Thauera sp. MZ1T]	gi|217969173	6.039999962	MEAVSSGMNPVSR	0	12.88074	15.00368	14.64689		1.164815065	1.137115569
387	integrase [Thauera sp. MZ1T]	gi|217969499	22.98999977	EEVLLSVDQTK	18.3244	17.24365	17.05658	17.77115	0.941021261	0.930812469	0.969808016
388	carbohydrate-selective porin OprB [Thauera sp. MZ1T]	gi|217969898	14.53999996	TSLVQDPSNIAGLLLTAHFPR	17.32611	16.26482	15.48117	16.5695	0.938746204	0.893516779	0.956331225
389	ATP-binding protein [Thauera sp. MZ1T]	gi|217970515	23.69000053	DAPSSASVKARR	16.12775	15.66593	15.76172	15.79526	0.971364884	0.977304336	0.979383981
390	PpiC-type peptidyl-prolyl cis-trans isomerase [Methylocella silvestris BL2]	gi|217976694	2.519999981	GFGANK	18.27264	17.39016	17.06877	17.58904	0.951704844	0.934116252	0.962588876
391	hypothetical protein Msil_1320 [Methylocella silvestris BL2]	gi|217977500	11.52000046	EGKAAREAMK	17.40512	15.27971	14.93167	15.59039	0.877885932	0.857889518	0.895735852
392	AraC family transcriptional regulator [Methylocella silvestris BL2]	gi|217978798	14.77000046	RGAAKECAEGR	15.41428	14.13737	14.03298	13.74081	0.917160581	0.910388289	0.891433787
393	HypF2 [Ralstonia eutropha H16]	gi|2183281	0.340000004	GYTPR	0	17.39291	16.74741	17.20207		0.962887176	0.989027713
394	precorrin-6y C5,15-methyltransferase subunit CbiE [Methylobacterium extorquens CM4]	gi|218529161	6.400000095	GCGGSR	18.19861	17.59929	16.96646	17.42092	0.967067815	0.93229428	0.957266517
395	inner-membrane translocator [Methylobacterium extorquens CM4]	gi|218530037	11.22999954	TPILG	16.86047	14.09626	16.00523	16.05723	0.836053799	0.949275435	0.952359572
396	penicillin-binding protein 1C [Escherichia fergusonii ATCC 35469]	gi|218548044	10.71000004	DGVIIK	17.69961	15.77143	16.19423	8.656915	0.891060876	0.914948408	0.48910202
397	TonB-denpendent outer membrane receptor [Campylobacter jejuni subsp. jejuni NCTC 11168 = ATCC 70081	gi|218561857	13.02999973	GMSGFGR	17.21152	16.2593	15.46162	16.32089	0.944675427	0.898329723	0.948253844
398	sensory trasnduction histidine kinase [Campylobacter jejuni subsp. jejuni NCTC 11168 = ATCC 700819]	gi|218562509	6.21999979	GIEEDK	18.06254	17.11021	16.84539	17.28888	0.947275965	0.932614682	0.957167707
399	putative molybdate metabolism protein [Escherichia coli IAI39]	gi|218699294	16.02000046	IKFAALQEHASDKINMVAKNR	16.43933	15.1268	14.23694	15.36086	0.920159155	0.866029212	0.934396961
400	electron transfer flavoprotein subunit alpha [Neisseria meningitidis Z2491]	gi|218767263	21.63999939	QVAGVEK	17.31255	16.356	16.1783	16.5477	0.944748174	0.934483944	0.955821066
401	TLDD protein-like protein [Neisseria meningitidis Z2491]	gi|218767363	14.38000011	GTSVFSGR	0	15.3087	14.83999	15.09808		0.969382769	0.98624181
402	metallophosphoesterase [Desulfatibacillum alkenivorans AK-01]	gi|218778720	11.02999973	AMFEAAKASSNPMR	17.4348	16.02171	16.68561	16.94912	0.918950031	0.957029045	0.97214307
403	transcriptional regulator [Desulfatibacillum alkenivorans AK-01]	gi|218779564	13.02000046	ASYLHACLKYVMR	15.98087	15.61153	14.65393	0	0.976888617	0.916966974	0
404	hypothetical protein Dalk_3483 [Desulfatibacillum alkenivorans AK-01]	gi|218781321	11.11999989	DMAAAVAKAER	16.37906	16.15199	15.46712	16.66693	0.986136567	0.944322812	1.01757549
405	hypothetical protein Dalk_3847 [Desulfatibacillum alkenivorans AK-01]	gi|218781683	13.02999973	MAAAKQLWILTGGNGAGK	15.43623	14.02572	14.64071	15.23435	0.908623414	0.9484641	0.986921677
406	NQR2 and RnfD family protein [Desulfatibacillum alkenivorans AK-01]	gi|218782184	15.27000046	VVENE	17.84692	14.67769	16.67981	16.326	0.82242146	0.934604402	0.914779693
407	hypothetical protein Dalk_4466 [Desulfatibacillum alkenivorans AK-01]	gi|218782295	11.89000034	IRTHPEESLGVLAR	16.8272	13.33196	16.32688	15.94138	0.792286298	0.970267186	0.947357849
408	ornithine carbamoyltransferase [Desulfovibrio vulgaris str. 'Miyazaki F']	gi|218885154	16.72999954	TPDLSTVR	16.5627	16.0624	15.46529	15.78228	0.969793572	0.933742083	0.952880871
409	Fis family transcriptional regulator [Desulfovibrio vulgaris str. 'Miyazaki F']	gi|218886588	10.98999977	GQTGMDLPGSAPDGVPPTLRTHK	0	15.31383	14.92943	15.07392		0.974898507	0.984333769
410	phage tape measure protein [Desulfovibrio vulgaris str. 'Miyazaki F']	gi|218886738	10.94999981	GDLGVKVAGGTGGMPTK	16.50281	13.85693	14.26481	14.79348	0.839670941	0.864386732	0.896421882
411	peptidase U32 [Desulfovibrio vulgaris str. 'Miyazaki F']	gi|218887231	18.44000053	KGGGEGGAVDFTPTYPAPGK	17.7664	14.77958	14.62957	15.28936	0.831883781	0.823440314	0.860577269
412	putative periplasmic chaperone [Burkholderia cepacia]	gi|22035178	15.48999977	YRVGGMADVQQK	17.79698	16.23736	16.57943	17.38542	0.912366031	0.931586707	0.976874728
413	integral membrane protein MviN [Desulfovibrio desulfuricans subsp. desulfuricans str. ATCC 27774]	gi|220903929	10.28999996	LPHVLRR	16.93384	16.05403	15.46488	15.15803	0.948044271	0.913252989	0.895132468
414	Peptidase C13, legumain asparaginyl peptidase [Methylobacterium nodulans ORS 2060]	gi|220914712	16.22999954	MLDASGAK	18.03901	16.7471	16.73222	17.06932	0.928382433	0.927557554	0.946244833
415	TonB-dependent receptor plug [Anaeromyxobacter dehalogenans 2CP-1]	gi|220915482	13.10999966	VAADVGTSVGPGAVLLGATAETTSGR	17.62141	0	15.82184	16.33537	0	0.897875936	0.927018326
416	tRNA pseudouridine synthase D TruD [Anaeromyxobacter dehalogenans 2CP-1]	gi|220918870	16.62999916	WLAERMADGLFAAALAGDAMKK	0	19.15086	17.2791	16.92116		0.902262353	0.883571808
417	hypothetical protein Mnod_1292 [Methylobacterium nodulans ORS 2060]	gi|220921295	7.409999847	GFIRG	17.00102	15.6987	15.62762	15.919	0.923397537	0.919216612	0.936355583
418	coenzyme F390 synthetase-like protein [Methylobacterium nodulans ORS 2060]	gi|220924287	6.360000134	TGARRHA	18.36341	17.71065	17.16917	0	0.964453225	0.934966327	0
419	O-methyltransferase family 2 [Methylobacterium nodulans ORS 2060]	gi|220925549	3.339999914	GPSSLAEMR	15.68412	14.42688	14.36423	14.64747	0.91983994	0.915845454	0.933904484
420	tRNA/rRNA methyltransferase SpoU [Methylobacterium nodulans ORS 2060]	gi|220925797	7.989999771	LAARPA	17.4245	15.42467	16.1555	16.64058	0.885228844	0.927171511	0.955010474
421	hypothetical protein Mnod_6673 [Methylobacterium nodulans ORS 2060]	gi|220926436	8.470000267	MSGMIGGTPVSSAR	16.1204	14.22196	15.78926	16.21547	0.882233691	0.979458326	1.005897496
422	major facilitator superfamily protein [Methylobacterium nodulans ORS 2060]	gi|220926696	13.14999962	MTFGSK	0	15.41473	15.09276	17.86308		0.979112836	1.158831845
423	glycerophosphoryl diester phosphodiesterase [Methylobacterium nodulans ORS 2060]	gi|220927197	13.31000042	AEDLARLALMGSAER	15.64487	14.40981	15.87162	15.24114	0.921056551	1.014493569	0.974194097
424	methyl-accepting chemotaxis sensory transducer [Thioalkalivibrio sulfidophilus HL-EbGr7]	gi|220933344	14.46000004	EAAAVMESSRKQASQSVEQAGK	17.3663	15.55389	15.56958	15.42139	0.895636376	0.89653985	0.888006657
425	HAD-superfamily hydrolase [Thioalkalivibrio sulfidophilus HL-EbGr7]	gi|220933470	18.60000038	MLSTMGFR	16.87867	16.56006	15.62756	15.824	0.981123513	0.925876269	0.937514626

426	hypothetical protein Tgr7_1055 [Thioalkalivibrio sulfidophilus HL-EbGr7]	gi|220934230	22.87000084	EIQMLAAATAQGIER	16.5293	16.6446	16.01491	14.9299	1.006975492	0.96888011	0.903238492
427	DNA polymerase III epsilon-like protein [Caulobacter crescentus NA1000]	gi|221234528	12.42000008	ALPQRLGMTPGR	17.07127	16.08099	15.837	16.24662	0.941991428	0.927698994	0.951693694
428	hypothetical protein CCNA_01862 [Caulobacter crescentus NA1000]	gi|221234799	16.71999931	ARSTRLSAGVPDQWLRALHR	0	15.15813	15.40242	15.39536		1.016116104	1.015650347
429	conserved hypothetical protein [Caulobacter crescentus NA1000]	gi|221236034	13.46000004	ATGMGGMTGNR	16.43864	15.34914	15.4999	15.81206	0.933723228	0.942894303	0.961883708
430	Holliday junction DNA helicase RuvB [Yersinia pestis KIM10+]	gi|22126138	10.75	ALDMLNVDAEGFDFMDR	16.91978	14.25239	14.07412	15.2422	0.842350787	0.831814598	0.900850957
431	DNA-binding transcriptional regulator [Yersinia pestis KIM10+]	gi|22126754	11.10999966	GSGTR	17.32849	16.12385	15.87043	16.40898	0.930482114	0.915857643	0.946936519
432	hypothetical protein y3785 [Yersinia pestis KIM10+]	gi|22127656	16.38999939	ASLTPVSIPVR	18.46751	17.99634	0	17.61629	0.974486544	0	0.953907159
433	RhaT family, DMT superfamily transporter [Rhodobacter sphaeroides KD131]	gi|221640097	21.5	SSGAAVLVWGYSRFWRRDR	18.87881	17.67738	17.9404	18.05312	0.936360925	0.950292947	0.956263663
434	phenylacetate--CoA ligase [Geobacter daltonii FRC-32]	gi|222053273	21.96999931	DIKDVVGITVK	0	17.75463	16.44782	16.99242		0.926396101	0.95706979
435	hypothetical protein Geob_0436 [Geobacter daltonii FRC-32]	gi|222053544	10.31999969	EFSFFTDSDR	15.69288	15.23234	14.70344	15.83212	0.970652933	0.93694975	1.008872814
436	phospholipase D/Transphosphatidylase [Geobacter daltonii FRC-32]	gi|222054850	15.55000019	NTDAAASR	17.431	16.65917	16.29356	16.3774	0.955720842	0.934746142	0.939555964
437	chemotaxis protein CheB [Geobacter daltonii FRC-32]	gi|222055260	23.64999962	AAKAAGGGVFAESDESAVVFGMPR	17.21386	15.91332	15.46448	15.19454	0.92444809	0.898373752	0.882692203
438	Ku protein [Agrobacterium vitis S4]	gi|222080132	16.54999924	QAMEAEDVVGVSR	20.41548	16.4007	18.41344	18.57859	0.803346284	0.901935198	0.910024648
439	phosphate transport system regulatory protein PhoU [Agrobacterium radiobacter K84]	gi|222084783	6.699999809	VIAVQGHGVPR	16.90354	15.8323	15.89662	16.43304	0.936626292	0.940431413	0.972165594
440	hypothetical protein Arad_2643 [Agrobacterium radiobacter K84]	gi|222086181	20.53000069	INETLGSGSDAIDGILTSSIER	17.88799	15.84562	15.68874	15.96029	0.885824511	0.877054381	0.892234958
441	transporter protein [Agrobacterium radiobacter K84]	gi|222087173	14.42000008	KAAGGH	18.96995	17.31163	18.24331	18.17426	0.912581741	0.961695207	0.95805524
442	conjugal transfer protein TrbG [Agrobacterium radiobacter K84]	gi|222109058	16.04999924	MDRAALKGMAPNMNIR	18.49521	16.31153	17.80217	17.29016	0.881932673	0.962528676	0.934845292
443	hypothetical protein Dtpsy_2120 [Acidovorax ebreus TPSY]	gi|222111310	2.920000076	HSAIQASR	15.92023	14.83196	14.60388	15.08533	0.931642319	0.917315893	0.94755729
444	hypothetical protein Dtpsy_2674 [Acidovorax ebreus TPSY]	gi|222111845	9	KDGGTIGI	16.48824	15.5147	16.18827	15.98799	0.940955493	0.981807033	0.969660194
445	hypothetical protein Avi_3077 [Agrobacterium vitis S4]	gi|222149263	12.84000015	TEISGAGAAGFAGNVLLGGVIGMGVD/	16.52585	0	15.27755	14.70192	0	0.924463795	0.889631698
446	Sun protein [Agrobacterium vitis S4]	gi|222150167	20.40999985	ESSLGTPGK	19.09779	17.95306	16.88537	18.34246	0.940059557	0.884153088	0.96044935
447	protein MtrB [Desulfobacterium autotrophicum HRM2]	gi|224367834	6.5	LGGYIRLASSKDR	15.82544	14.95203	14.63935	15.05533	0.944809749	0.925051689	0.951337214
448	putative YcaC-related amidohydrolase [Desulfobacterium autotrophicum HRM2]	gi|224370033	14.98999977	DAGGVATSCEMILFELMK	18.55217	16.51511	0	17.66388	0.890198289	0	0.952119348
449	hypothetical protein HRM2_38150 [Desulfobacterium autotrophicum HRM2]	gi|224370873	10.27000046	TMSPGK	16.24254	14.77313	13.98829	14.76634	0.909533238	0.861213209	0.9091152
450	hypothetical protein HRM2_41850 [Desulfobacterium autotrophicum HRM2]	gi|224371241	5.630000114	GCMGGPR	17.12652	16.41026	0	16.37114	0.958178311	0	0.955894134
451	cytoChrome c-553 [Nautilia profundicola AmH]	gi|224373302	10.56999969	NMSDADIEAAAKEFGKK	18.25567	16.69326	17.29569	17.29288	0.914415083	0.947414694	0.947260769
452	GumN family protein [Brucella melitensis ATCC 23457]	gi|225853355	10.86999989	NHLMVDR	15.98249	14.91087	14.97554	15.41934	0.932950373	0.936996676	0.964764564
453	pyridoxamine 5'-phosphate oxidase [Laribacter hongkongensis HLHK9]	gi|226940936	12.89000034	NIADMRKDYK	17.25897	17.55978	15.67794	15.59439	1.017429198	0.908393722	0.903552761
454	hypothetical protein Avin_12270 [Azotobacter vinelandii DJ]	gi|226943356	14.31000042	GRSVSLGELRAGAGR	17.84882	14.78358	16.13181	16.63438	0.828266518	0.903802604	0.931959648
455	dTDP-4-dehydrorhamnose reductase RmlD [Azotobacter vinelandii DJ]	gi|226944176	11.86999989	SAGVPR	19.30112	16.90835	18.46145	18.75381	0.876029474	0.956496307	0.971643614
456	hypothetical protein NGR_b23030 [Sinorhizobium fredii NGR234]	gi|227820530	12.81999969	GGGTTLLTAIAAR	16.48131	15.2949	15.31449	15.57391	0.928014824	0.929203443	0.944943697
457	xylose isomerase [Sinorhizobium fredii NGR234]	gi|227823471	18.12000084	DTMDAAK	16.81428	15.82013	15.80497	16.01938	0.940874661	0.939973047	0.952724708
458	threonyl-tRNA synthetase [Pseudomonas fluorescens SBW25]	gi|229591579	6.380000114	GRPDSE	16.69243	14.355	14.7338	16.03293	0.859970657	0.882663579	0.960491073
459	type I site-specific deoxyribonuclease HsdR family [Vibrio cholerae MJ-1236]	gi|229606287	14.98999977	DGIDSLFTMISGLFNKKR	17.9732	14.30283	16.16812	17.37632	0.795786504	0.899568246	0.966790555
460	family 5 extracellular solute-binding protein [Thauera sp. MZ1T]	gi|237654208	21.95000076	SGPENR	19.51411	18.66992	18.33258	18.8501	0.956739508	0.939452529	0.965972827
461	50S ribosomal protein L3 [Tolumonas auensis DSM 9187]	gi|237806875	9.68999958	NLLLIKGAVPGATNGDVIVKPAVKA	16.10904	15.65159	14.48898	14.44443	0.971602901	0.899431623	0.896666096
462	transport-associated [Tolumonas auensis DSM 9187]	gi|237807199	12.64000034	RSVTAQMDDQKIELNTR	16.7804	14.79654	15.24505	15.77817	0.881775166	0.908503373	0.940273772
463	AsmA family protein [Tolumonas auensis DSM 9187]	gi|237808080	18.89999962	GSTSAPK	17.25924	16.4203	16.42523	15.8324	0.951391834	0.951677478	0.917328921
464	hypothetical protein Tola_2394 [Tolumonas auensis DSM 9187]	gi|237809137	10.43000031	IAHPVITLEAGQAMLVER	17.91084	16.05412	16.26379	16.56397	0.896335404	0.908041722	0.924801405
465	PvdI [Burkholderia glumae BGR1]	gi|238024382	9.600000381	ERVLEAQAHADLPFTK	17.27294	14.88589	14.62384	14.39011	0.86180407	0.846632941	0.833101371
466	hypothetical protein [Burkholderia glumae BGR1]	gi|238025830	11.55000019	DLARELDAAGFDR	16.52436	15.13394	16.65917	15.91859	0.915856348	1.008158258	0.963340789
467	hypothetical protein [Burkholderia glumae BGR1]	gi|238027073	10.86999989	IARAASDMEDK	17.47479	16.38329	16.34251	0	0.937538591	0.935204944	0
468	Ribonuclease R [Burkholderia glumae BGR1]	gi|238027544	12.98999977	AMPGVTSEDEPAVGRR	16.17501	16.20944	0	15.6893	1.002128592	0	0.96997158
469	5-formyltetrahydrofolate cyclo-ligase family protein [Burkholderia glumae BGR1]	gi|238028860	2.869999886	SGSTA	16.23275	14.87207	14.51066	14.84634	0.916176865	0.893912615	0.914591797
470	Glyoxalase family protein [Burkholderia glumae BGR1]	gi|238028956	16.42000008	TLDMTAMTR	18.46867	0	17.12786	16.98869	0	0.927400836	0.919865372
471	PAS/PAC sensor signal transduction histidine kinase [Variovorax paradoxus S110]	gi|239818115	22.46999931	WALSAFARLR	18.35343	16.54217	0	17.52378	0.90131218	0	0.954795916
472	NAD-dependent DNA ligase [Desulfovibrio magneticus RS-1]	gi|239906999	17.26000023	GEVVMTR	19.15192	16.22885	17.85988	18.28958	0.847374571	0.932537312	0.954973705
473	single-stranded-DNA-specific exonuclease [Desulfovibrio magneticus RS-1]	gi|239907925	8.760000229	ILAKNGMLLLAEAR	16.26464	16.62329	14.77275	15.63699	1.022050903	0.908274023	0.961410151
474	3-octaprenyl-4-hydroxybenzoate carboxy-lyase [Desulfovibrio magneticus RS-1]	gi|239907991	3.930000067	VRGGGDAK	16.57623	14.51402	15.57037	15.19021	0.875592339	0.939319133	0.916385089
475	tartronate semialdehyde reductase [Desulfovibrio magneticus RS-1]	gi|239908136	14.77999973	GGLAGSTVLDAKAPLVMDRK	17.81163	14.44814	14.38244	15.18487	0.811163268	0.807474667	0.852525569
476	phage integrase [Bartonella grahamii as4aup]	gi|240850374	16.29000092	GPLAEKCMYIYMR	17.06578	17.66804	15.71939	15.41833	1.035290505	0.921105862	0.903464711
477	amidohydrolase [Rhizobium leguminosarum bv. trifolii WSM1325]	gi|241206583	7.599999905	GKGEGSR	17.8601	17.11834	16.68263	17.11066	0.958468318	0.934072598	0.958038309
478	extracellular solute-binding protein [Rhizobium leguminosarum bv. trifolii WSM1325]	gi|241207018	25.28000069	AGIPLPTPDLTWDEYAK	0	17.04261	18.54508	18.45124		1.088159619	1.08265342
479	Microcystin LR degradation protein MlrC-like protein [Rhizobium leguminosarum bv. trifolii WSM1325]	gi|241666544	6.78000021	MIDVFPTSREPMR	15.55558	0	13.84358	14.8187	0	0.88994303	0.952629217
480	outer membrane receptor FepA [Dickeya dadantii Ech703]	gi|242238132	8.350000381	AEGATK	17.65385	15.89399	16.45931	0	0.900312963	0.93233544	0
481	hypothetical protein Dd703_0687 [Dickeya dadantii Ech703]	gi|242238139	22.46999931	MTTETVTVER	17.21398	17.17448	0	16.48127	0.997705353	0	0.957435178
482	4Fe-4S ferredoxin [Dickeya dadantii Ech703]	gi|242240582	15.10000038	DALTGIVDVNDK	0	17.12324	16.81211	17.24785		0.981829957	1.007277244
483	small multidrug resistance protein [Desulfovibrio salexigens DSM 2638]	gi|242278406	7.079999924	LSGASH	18.3033	17.63898	16.3666	17.58697	0.963704906	0.89418848	0.960863342
484	hypothetical protein Desal_1140 [Desulfovibrio salexigens DSM 2638]	gi|242278613	18.70000076	VPEQLSPMEFEPVR	17.23807	15.1698	15.4694	15.93592	0.880017311	0.897397446	0.924460801
485	hypothetical protein Desal_1653 [Desulfovibrio salexigens DSM 2638]	gi|242279125	15.86999989	QGFEKILAEGMFHAEASNSGK	18.80492	17.63354	0	18.08079	0.937708855	0	0.961492524
486	DSBA oxidoreductase [Desulfovibrio salexigens DSM 2638]	gi|242280620	13.60999966	EALGQGVYLAK	17.00694	15.53464	15.82484	15.88905	0.913429459	0.930493081	0.934268599

487	cyclopropane-fatty-acyl-phospholipid synthase CfaS [Shewanella oneidensis MR-1]	gi|24374890	15.14000034	MENTASQSSVVTAKLPDSLARKLLLK	15.81894	13.35355	14.13574	14.68592	0.844149482	0.893595905	0.928375732
488	ABC-type efflux system permease component 1 [Shewanella oneidensis MR-1]	gi|24375575	13.31999969	AQLPNEPTRLSEPK	16.61252	14.36447	13.85889	14.71501	0.864677364	0.834243691	0.885778317
489	AF440524_59 hypothetical protein [Pseudomonas aeruginosa]	gi|24461703	5.309999943	AGGTP	17.68731	16.62425	16.3633	16.7817	0.939897022	0.925143507	0.948798885
490	galactose-1-phosphate uridylyltransferase [Dickeya zeae Ech1591]	gi|251790467	19.71999931	DDSHSR	16.98798	16.05174	15.44745	16.16476	0.944888091	0.90931647	0.951541031
491	GCN5-related N-acetyltransferase [Dickeya zeae Ech1591]	gi|251791286	24.10000038	MLPDGNGELKSMR	0	16.26548	15.99151	16.53058		0.983156353	1.01629832
492	fructose 1,6-bisphosphatase II [Aggregatibacter aphrophilus NJ8700]	gi|251793663	15.77999973	DDNLVFAATGITNGDLLKGVHR	18.99977	15.93064	13.87404	13.64019	0.838464887	0.730221471	0.717913427
493	type II secretion system protein E [Pectobacterium carotovorum subsp. carotovorum PC1]	gi|253687071	14.36999989	IAAAVGRRIDEASPMVDAR	0	15.47171	16.17485	16.37077		1.045446819	1.058109931
494	hypothetical protein [Franconibacter pulveris]	gi|254262267	9.159999847	EANEG	16.25494	14.03684	15.71696	16.23107	0.863543021	0.9669036	0.998531523
495	peptidase M48 Ste24p [Hirschia baltica ATCC 49814]	gi|254293479	12.93000031	AVMGAFGMGADMGVMKPFSR	17.56282	15.30064	15.55813	16.23126	0.871194945	0.88585603	0.924183018
496	hypothetical protein Hbal_2097 [Hirschia baltica ATCC 49814]	gi|254294454	15.19999981	SGSLVVNSSQGGGTKDTWVLKE	17.35644	16.35466	16.01488	16.57457	0.942281943	0.922705347	0.954952168
497	response regulator receiver domain-containing protein [Teredinibacter turnerae T7901]	gi|254784742	9.659999847	DSLAVGS	18.24685	17.58474	17.1117	17.69257	0.963713737	0.937789262	0.96962325
498	GTP-binding protein LepA [Teredinibacter turnerae T7901]	gi|254785334	14.25	EILGAPVGDTITHSNTADVK	16.89143	16.8209	15.99748	16.27315	0.99582451	0.947076713	0.963396823
499	RtcB protein [Teredinibacter turnerae T7901]	gi|254786555	12.25	ARRGEMGIIPGSMGAR	0	15.39927	14.87474	14.36395		0.965937996	0.932768242
500	ATP-NAD/AcoX kinase [Neorickettsia risticii str. Illinois]	gi|254796691	10.22000027	CIDASEGVKPSMILALGGDGFMLDTLF	15.95774	14.48313	15.0069	0	0.907592804	0.940415121	0
501	thioredoxin [Neorickettsia risticii str. Illinois]	gi|254797223	17.31999969	AVPTLILFK	15.98058	15.08063	14.83486	15.63401	0.943684772	0.928305481	0.978313052
502	hypothetical protein NMO_0058 [Neisseria meningitidis alpha14]	gi|254804082	13.06000042	VIGYLCAK	15.2821	14.08856	14.20635	14.60096	0.921899477	0.929607187	0.9554289
503	DNA-binding transcriptional regulator AsnC [Kangiella koreensis DSM 16069]	gi|256821790	14.47000027	IEKMRQAGIIKGTK	17.43803	15.64977	16.65291	16.71269	0.897450572	0.954976566	0.958404705
504	S-adenosyl-methyltransferase MraW [Kangiella koreensis DSM 16069]	gi|256821906	28.68000031	IARAIVEKRAK	18.89869	16.8246	17.16061	18.72402	0.890252182	0.908031721	0.99075756
505	ribose-phosphate pyrophosphokinase [Kangiella koreensis DSM 16069]	gi|256822532	12.97999954	YGDFEVEVTLPDIALHKHR	16.12349	15.99002	14.8047	0	0.991722016	0.918206914	0
506	ATP-dependent metalloprotease FtsH [Kangiella koreensis DSM 16069]	gi|256823581	9.090000153	GGGAMSFGKSK	15.90655	14.71193	14.67322	14.91862	0.924897605	0.922464016	0.937891623
507	DNA polymerase I [Kangiella koreensis DSM 16069]	gi|256823707	8.569999695	TNPITDR	16.68358	15.97431	14.83819	10.37639	0.957486942	0.889388848	0.621952243
508	VacJ family lipoprotein [Desulfomicrobium baculatum DSM 4028]	gi|256830422	19.84000015	KASVSLGR	18.17263	17.14034	16.45249	17.33826	0.943195344	0.905344466	0.954086448
509	TniA [Pseudomonas putida]	gi|257070307	26.88999939	VAVVEHGR	16.50965	13.73234	16.84419	17.87652	0.831776567	1.020263301	1.082792185
510	primosomal protein N' [Candidatus Accumulibacter phosphatis clade IIA str. UW-1]	gi|257091721	10.61999989	ERGQLLVESSSRR	18.00562	15.20932	12.74671	12.94989	0.844698489	0.707929524	0.719213779
511	hypothetical protein CAP2UW1_1114 [Candidatus Accumulibacter phosphatis clade IIA str. UW-1]	gi|257092736	9.43999958	DIFAAA	17.09712	15.72639	15.88383	15.99498	0.919826848	0.929035416	0.935536511
512	transcriptional regulatory LysR family protein [Bradyrhizobium elkanii]	gi|257796111	11.56999969	VGSNASREMIESWMCSR	17.67458	15.4891	16.1134	16.72928	0.876348971	0.911670886	0.946516409
513	phosphoribosylglycinamide synthetase [Desulfohalobium retbaense DSM 5692]	gi|258404752	7.630000114	AVHALGIDWGPVK	17.1009	16.01441	15.88544	16.18404	0.936465917	0.928924209	0.946385278
514	dinitrogenase iron-molybdenum cofactor biosynthesis protein [Desulfohalobium retbaense DSM 5692]	gi|258405374	14.72999954	GRGPGQGGRGMGGGGGQRR	18.10142	16.07896	17.06144	16.98247	0.888270644	0.942547049	0.938184408
515	conjugal transfer protein TraG/TraD/DotL/TrbC/IcmO [Acetobacter pasteurianus IFO 3283-01]	gi|258513181	20.79999924	QHAAMPGTTGAGK	15.34462	14.72327	14.70076	14.83087	0.95950698	0.958040017	0.96651921
516	hypothetical protein Csp_D30990 [Curvibacter putative symbiont of Hydra magnipapillata]	gi|260222543	10.67000008	NFELACLELSMERWMAQSGK	16.96203	13.79164	14.14946	14.71412	0.813088999	0.834184352	0.867473999
517	CheA signal transduction histidine kinase [Halothiobacillus neapolitanus c2]	gi|261855333	6.510000229	VTPAIMDVMLR	16.24518	15.82471	15.1112	15.13652	0.974117246	0.93019591	0.931754527
518	hypothetical protein Hneap_1637 [Halothiobacillus neapolitanus c2]	gi|261856227	13.22000027	YQFKSPVGRLSIDR	15.69859	15.26595	0	14.90571	0.972440837	0	0.949493553
519	serine/threonine protein kinase [Haliangium ochraceum DSM 14365]	gi|262194763	11.81000042	KRLGVTPFEWTVEASPR	18.53032	15.74168	16.62533	16.75424	0.849509345	0.897196055	0.904152762
520	hypothetical protein [Haliangium ochraceum DSM 14365]	gi|262195538	12.56999969	NGPSGGGQG	16.49319	14.68168	14.78748	15.18482	0.890166184	0.896580953	0.920672108
521	acetyl-CoA carboxylase, carboxyl transferase subunit beta [Haliangium ochraceum DSM 14365]	gi|262196940	22.87999916	TDGLPSEPK	16.14802	15.72035	14.76537	15.35731	0.973515638	0.914376499	0.951033625
522	isochorismate synthase [Haliangium ochraceum DSM 14365]	gi|262198877	12.40999985	LVARSGK	17.71801	16.70783	16.41176	16.92984	0.942985696	0.926275581	0.955515885
523	hypothetical protein [Haliangium ochraceum DSM 14365]	gi|262199312	10.10000038	GEIAQPEVYALIEHADQDLSAVR	15.19836	14.51769	14.37176	0	0.955214247	0.945612553	0
524	alpha/beta hydrolase [Vibrio sp. Ex25]	gi|262393055	22.39999962	FPDNPK	21.07129	20.23051	19.78379	20.00786	0.960098314	0.938897903	0.949531804
525	hypothetical protein c2404 [Escherichia coli CFT073]	gi|26248256	13.65999985	MFSESHLSSISGTKMK	15.50869	14.01969	14.3699	15.21303	0.903989312	0.926570845	0.98093585
526	transposase IS116/IS110/IS902 family protein [Comamonas testosteroni CNB-2]	gi|264677652	10.02999973	AAAIKGLGPLTASAMVATVGDFR	0	16.04698	15.48143	15.22653		0.964756608	0.948872
527	amide-urea binding protein [Methylophilus methylotrophus]	gi|2661835	13.77999973	MSTSNRRGFMK	17.61554	15.70293	16.16217	16.97626	0.891424844	0.917495007	0.963709316
528	FAD-dependent pyridine nucleotide-disulfide oxidoreductase [Sulfurospirillum deleyianum DSM 6946]	gi|268679827	18.72999954	EDKVGGLLTYGIPNFKLPK	17.10538	16.51662	15.95816	14.94739	0.96558042	0.932932212	0.873841446
529	chemotaxis sensory transducer [Sulfurospirillum deleyianum DSM 6946]	gi|268680643	2.690000057	ECGGEK	16.58857	14.72298	14.62428	15.09124	0.887537624	0.881587744	0.909737247
530	binding-protein-dependent transporters inner membrane component [Sulfurospirillum deleyianum DSM 6946]	gi|268680652	8.600000381	ATGVAPLR	15.89626	15.11656	14.69794	15.07508	0.950950727	0.92461623	0.948341308
531	flavocytochrome C [Sulfurospirillum deleyianum DSM 6946]	gi|268680705	21.09000015	GVILASGGFSMDK	16.96862	15.92341	15.58025	15.95779	0.938403359	0.918180147	0.940429452
532	hypothetical protein ETAE_1497 [Edwardsiella tarda EIB202]	gi|269138848	8.649999619	QGRTLIYGGGDK	18.20179	15.27	16.87531	13.20905	0.838928479	0.927123651	0.725700604
533	ATP-dependent transcriptional regulator MalT-like, LuxR family [Edwardsiella tarda EIB202]	gi|269140644	13.39000034	ALPMMQQTEQVARR	16.69167	16.25515	0	15.90687	0.973848033	0	0.952982536
534	3-dehydroquinate synthetase [uncultured SUP05 cluster bacterium]	gi|269467790	7.420000076	SMAVDK	16.09789	15.20008	14.92026	15.34313	0.944228094	0.926845692	0.953114352
535	hypothetical protein PP_0627 [Pseudomonas putida KT2440]	gi|26987363	8.430000305	MTSRLNPEDQR	17.47613	14.81936	14.83869	15.1473	0.847977212	0.849083292	0.866742236
536	RNA polymerase beta subunit [Gluconobacter cerinus]	gi|269912626	12.38000011	MNMRLDVXAPDTLR	15.80083	14.92274	15.9358	15.75213	0.944427603	1.008541956	0.996917883
537	conserved hypothetical protein [Erwinia pyrifoliae Ep1/96]	gi|27228714	11.39000034	MANHSSK	16.59681	15.46675	0	15.70463	0.931911012	0	0.946243887
538	hypothetical protein bsr2105 [Bradyrhizobium diazoefficiens USDA 110]	gi|27377216	3.430000067	GAVGPHG	18.23277	17.25144	16.8147	17.3012	0.946177679	0.922224105	0.948906831
539	site-specific integrase/recombinase [Bradyrhizobium diazoefficiens USDA 110]	gi|27377249	18.87999916	GYLRFLSARGLCR	18.81553	16.17986	13.76411	14.18838	0.859920502	0.731529221	0.754078147
540	hypothetical protein bll8275 [Bradyrhizobium diazoefficiens USDA 110]	gi|27383386	9.420000076	RSVADVMGVSALVMR	17.94676	0	14.64973	15.44259	0	0.816288288	0.860466736
541	electron transport complex protein RnfG [Buchnera aphidicola str. Bp (Baizongia pistaciae)]	gi|27904615	19.05999947	KKQDITAVIFETIAPDGYSGIIK	0	14.97097	14.40601	14.29852		0.962262966	0.955083071
542	hypothetical protein PD1002 [Xylella fastidiosa Temecula1]	gi|28198900	14.14999962	DGTPYLTAADLARALGYADER	17.14367	17.21158	15.48926	16.4314	1.003961229	0.903497326	0.958452887
543	DNA polymerase III subunit epsilon [Xylella fastidiosa Temecula1]	gi|28199104	23.84000015	ASVLDTLVMAR	18.48314	17.0432	17.36913	17.77649	0.922094406	0.939728315	0.96176786
544	phosphoribosylformimino-5-aminoimidazole carboxamide ribotide isomerase [Candidatus Blochmannia ocreat	gi|282555100	7.170000076	IVLGSMSVTQPK	18.64862	17.21274	17.6411	17.81548	0.923003418	0.945973482	0.955324308
545	fimbrial usher protein [Citrobacter rodentium ICC168]	gi|283786592	19.64999962	VENQQRQQGWR	16.80891	15.23492	14.65373	14.80988	0.906359782	0.871783477	0.881073193
546	hypothetical protein ROD_40921 [Citrobacter rodentium ICC168]	gi|283787653	20.45000076	MFFLLSNEMSGKGGER	19.35279	17.67193	0	18.49509	0.913146373	0	0.955680809
547	multidrug efflux system subunit MdtB [Arsenophonus nasoniae]	gi|284006852	11.52000046	TAGMAAGQPAIILVIR	15.89195	15.69725	14.89798	15.36331	0.987748514	0.937454497	0.96673536

548	UDP-N-acetyl-D-mannosaminuronic acid dehydrogenase [Aeromonas hydrophila]	gi|28827174	6.690000057	MAMILPAITCLICQR	16.02031	13.96328	16.01939	14.59995	0.871598615	0.999942573	0.911340043
549	ATP-dependent Clp protease proteolytic subunit ClpP [Allochromatium vinosum DSM 180]	gi|288941151	1.049999952	GPVASK	15.89155	14.36772	14.88252	15.30116	0.904110675	0.93650525	0.96284881
550	membrane-associated zinc metalloprotease [Allochromatium vinosum DSM 180]	gi|288941765	14.26000023	LELIPRALDTDGQTVGR	16.64078	15.87788	14.7058	16.55361	0.954154793	0.883720595	0.994761664
551	SoxXA-binding protein [Allochromatium vinosum DSM 180]	gi|288941883	6.099999905	DTAAMIAR	17.91672	16.80491	16.72417	17.16776	0.937945673	0.933439268	0.958197706
552	Flp/Fap pilin component [Azospirillum sp. B510]	gi|288956966	17.85000038	ISAKTPT	17.38068	16.37895	15.70062	16.57554	0.942365316	0.903337499	0.953676151
553	hypothetical protein AZL_006880 [Azospirillum sp. B510]	gi|288957529	11.19999981	APALTGSEGPK	17.11301	15.79982	15.67904	16.13272	0.923263646	0.916205857	0.942716682
554	zinc protease [Azospirillum sp. B510]	gi|288957544	17.47999954	AVLSQTNHVTGLLLPPTGKDS	17.77691	14.87305	14.1326	14.51186	0.836649901	0.794997556	0.816331972
555	two-component hybrid sensor and regulator [Azospirillum sp. B510]	gi|288958191	18.75	AIPELAGMR	15.534	13.6964	14.04358	14.87297	0.881704648	0.904054332	0.957446247
556	hypothetical protein AZL_a08880 [Azospirillum sp. B510]	gi|288960623	19.82999992	NSITGAPSPAAR	18.04355	14.00982	15.37168	17.30723	0.776444768	0.851921047	0.959192066
557	urea carboxylase [Azospirillum sp. B510]	gi|288961257	25.48999977	IGYPVMIKSTAGGGGIGMQLCR	18.50138	16.65042	17.00848	17.33954	0.899955571	0.919308722	0.937202522
558	methyl-accepting chemotaxis protein [Azospirillum sp. B510]	gi|288961435	10.30000019	SIVGPVRSMTTAMSVLAK	17.7423	14.80989	15.00935	15.56322	0.834722105	0.845964165	0.877181651
559	secreted protein [Legionella longbeachae NSW150]	gi|289164017	13.92000008	LGMLSNESFRISR	16.8459	13.76733	13.71449	14.1983	0.817251082	0.814114414	0.842834161
560	type II protein secretion LspD [Legionella longbeachae NSW150]	gi|289164527	12.86999989	QVDNSSTSGIDMIHLR	14.42506	14.78598	14.10758	12.56581	1.025020347	0.977991079	0.871109722
561	hypothetical protein LLO_3130 [Legionella longbeachae NSW150]	gi|289166439	5.46999979	FKKSA	16.03239	14.85425	15.2547	15.71589	0.926515011	0.951492572	0.980258714
562	capsular exopolysaccharide family protein [Thioalkalivibrio sp. K90mix]	gi|289207806	11.63000011	LAAINSLRGQFEAGQQGR	0	16.14561	16.27628	16.84413		1.008093222	1.043263773
563	Sb5 [Xenorhabdus bovienii SS-2004]	gi|290473739	13.67000008	PMMTKK	16.92566	15.59472	15.61537	16.05027	0.921365548	0.922585589	0.948280303
564	inorganic pyrophosphatase [Helicobacter mustelae 12198]	gi|291276848	16.68000031	VNVVIEVPYMSNIK	0	15.85108	15.89805	16.52452		1.002963205	1.042485433
565	type IV pilus secretin PilQ [Sideroxydans lithotrophicus ES-1]	gi|291612584	15.64000034	ESMGMN	18.22633	17.28853	16.90469	17.11007	0.948546965	0.927487322	0.938755635
566	hypothetical protein PANA_0471 [Pantoea ananatis LMG 20103]	gi|291616024	17.37999916	GRKMTSVKAIAIDLINAGADFR	16.87202	16.4228	0	16.10816	0.973374854	0	0.954726227
567	RapA [Pantoea ananatis LMG 20103]	gi|291616242	13.89999962	VSGIMGTR	16.51051	15.57578	15.05992	0	0.943385759	0.912141418	0
568	30S ribosomal protein S14 [Nitrosococcus halophilus Nc 4]	gi|292492434	11.94999981	EVAMRGDVPGLVKASW	17.55927	16.53319	0	16.86738	0.941564769	0	0.960596881
569	AsmA protein [Sphingobium japonicum UT26S]	gi|294013437	8.640000343	LSPTPMGKLLAR	16.57224	14.85498	0	15.85165	0.896377315	0	0.95651825
570	ApbE family lipoprotein [Candidatus Puniceispirillum marinum IMCC1322]	gi|294084409	22.01000023	SMFGSAEDPVKQAEAGSVAMIK	21.45228	19.87406	20.09705	20.30161	0.92643113	0.936825829	0.946361412
571	OmpA-family protein [Sphingobium japonicum UT26S]	gi|294146707	2.75999999	TAGPEIK	15.54904	14.657	14.31378	14.85746	0.942630542	0.920557153	0.955522656
572	molybdate ABC transporter permease ModB [Rhodobacter capsulatus SB 1003]	gi|294677740	6.730000019	RAPAP	17.62068	16.65822	16.43888	16.94339	0.945378952	0.932931079	0.961562777
573	ABC transporter ATP-binding protein/permease [Rhodobacter capsulatus SB 1003]	gi|294677945	8.819999695	GTSGSGK	18.30132	17.71177	17.12324	17.58132	0.967786477	0.935628687	0.960658575
574	phosphoenolpyruvate carboxykinase [Rhodobacter capsulatus SB 1003]	gi|294678358	9.840000153	FDVLHADMLAHMKGK	16.68964	15.16837	15.29192	15.7353	0.908849442	0.916252238	0.942818419
575	integral membrane sensor signal transduction histidine kinase [Burkholderia sp. CCGE1002]	gi|295676684	23.98999977	TPLAAIKVQAQVALAEPDIGLQR	0	16.4736	16.70675	16.97572		1.014152948	1.030480284
576	hypothetical protein Cseg_0908 [Caulobacter segnis ATCC 21756]	gi|295688339	19.62000084	GLRWPREGFCQVYR	18.24467	15.96238	17.69264	18.43091	0.87490648	0.969742944	1.010207913
577	glutathione S-transferase [Caulobacter segnis ATCC 21756]	gi|295688760	15.60000038	QPDYLAK	16.02071	14.9963	14.67761	15.24432	0.936057141	0.916164764	0.951538353
578	glyoxalase II family Zn-dependent hydrolase [Caulobacter segnis ATCC 21756]	gi|295690071	11.32999992	TMKAAIIDPGGEVDRLLK	16.01669	14.39094	14.85756	0	0.898496506	0.927629866	0
579	hypothetical protein [Burkholderia sp. CCGE1002]	gi|295700059	8.579999924	AYLAGREDFENQPGMPEDVK	16.54217	14.14773	14.24697	14.77197	0.855252364	0.861251577	0.892988647
580	Chain A, Crystal Structure Of Plant Slac1 Homolog Teha	gi|295982514	10.60999966	FSFIALIPITTXLVGDILYR	17.40913	16.40014	16.30084	16.9101	0.94204248	0.936338576	0.971335156
581	hypothetical protein ECL_01934 [Enterobacter cloacae subsp. cloacae ATCC 13047]	gi|296102289	13.56999969	MVEAGASADESAKLAAVK	16.87102	14.22024	15.16914	15.04252	0.842879684	0.89912406	0.891618883
582	DNA gyrase subunit B [Moraxella catarrhalis RH4]	gi|296112234	13.75	GLGEMNKEQLWETTMDPENR	15.63063	0	13.95826	14.33065	0	0.893006872	0.916831247
583	hypothetical protein MCR_0990 [Moraxella catarrhalis RH4]	gi|296113210	11.22000027	DCSKAMAIK	15.40339	14.98618	14.43878	14.85234	0.972914404	0.937376772	0.964225408
584	XRE family transcriptional regulator [Arcobacter nitrofigilis DSM 7299]	gi|296273964	10.43000031	VSIEPNTNVILEVKDFGKHLK	17.40349	14.85432	14.70717	15.15481	0.853525356	0.845070155	0.870791433
585	phosphoserine phosphatase SerB [Methylotenera versatilis 301]	gi|297537989	23.5	LDYAVSNTLEIIDGK	0	19.18072	19.32488	19.93983		1.007515881	1.039576721
586	hypothetical protein DaAHT2_0024 [Desulfurivibrio alkaliphilus AHT2]	gi|297568015	12.89000034	RLTVCKPERFSMK	0	15.14256	14.85913	13.57853		0.981282557	0.896712973
587	sulfite reductase [Starkeya novella DSM 506]	gi|298292523	14.31000042	MPESSPAPGQPIGIDEAAATFDR	18.04322	16.33547	16.60675	0	0.90535226	0.92038727	0
588	FkbM family methyltransferase [Hyphomicrobium denitrificans ATCC 51888]	gi|300021980	12.19999981	AEFVSSGYGVAR	16.41957	15.11921	0	15.81675	0.92080426	0	0.963286493
589	biotin biosynthesis protein BioC [Nitrosococcus watsonii C-113]	gi|300113678	20.02000046	LKESVPQRLGR	17.65566	15.78155	15.98151	16.37455	0.89385217	0.905177716	0.927439133
590	hypothetical protein Nwat_2579 [Nitrosococcus watsonii C-113]	gi|300115107	16.68000031	QAVVTAQR	0	16.11009	15.71184	15.78578		0.975279468	0.979869138
591	hypothetical protein Hsero_2281 [Herbaspirillum seropedicae SmR1]	gi|300311596	5.699999809	GSPMTK	16.66322	15.79581	0	15.95214	0.947944635	0	0.957326375
592	transcription regulator protein [Herbaspirillum seropedicae SmR1]	gi|300312133	18.37999916	MLSSAYASSSR	18.18688	16.53093	16.94884	17.67844	0.908948099	0.931926752	0.972043583
593	transferase [Shigella flexneri 2a str. 2457T]	gi|30064447	3.019999981	STGGK	16.38883	15.72377	15.37781	15.7025	0.959419922	0.938310422	0.958122087
594	prolipoprotein signal peptidase (SPase II) [Ralstonia solanacearum PSI07]	gi|300690676	14.18000031	AKSNNGK	17.96019	16.04399	16.08573	17.06412	0.893308478	0.895632507	0.950107989
595	putative Phytanoyl-CoA dioxygenase [Ralstonia solanacearum PSI07]	gi|300693185	16.12000084	ALARPPECATLLAAAR	16.94126	0	14.42523	15.25886	0	0.851485073	0.900692156
596	putative ABC transporter ATP-binding protein [Ralstonia solanacearum PSI07]	gi|300693527	17.96999931	QDAEADARALLEKVGLADK	17.21376	15.671	16.07809	16.5307	0.91037635	0.934025454	0.960318954
597	protein of unknown function [Ralstonia solanacearum CFBP2957]	gi|300698095	18.45000076	AARRMAHK	16.42575	10.98803	14.52268	15.1036	0.668951494	0.884141059	0.919507481
598	conserved exported protein of unknown function [Ralstonia solanacearum CFBP2957]	gi|300704616	8.18999958	ASGVS	16.01516	14.9238	14.98275	15.2296	0.931854568	0.935535455	0.950948976
599	DNA mismatch repair protein [Erwinia billingiae Eb661]	gi|300718162	13.43000031	AANSKALTEGIGTNGQPIMTLPR	17.50309	17.0207	16.4115	16.83017	0.972439724	0.937634441	0.961554217
600	probable mobilization protein A [Salmonella enterica subsp. enterica serovar Enteritidis]	gi|302122901	14.30000019	GMSMSR	17.85943	16.89431	16.65975	17.08311	0.945960201	0.932826524	0.956531647
601	FAD linked oxidase domain-containing protein [Desulfarculus baarsii DSM 2075]	gi|302342938	9.949999809	GMMNPGK	17.85755	16.22881	16.25231	16.85735	0.90879264	0.91010861	0.943990077
602	NodT family RND efflux system outer membrane lipoprotein [Desulfarculus baarsii DSM 2075]	gi|302343867	11.36999989	ASADQRP	16.61823	13.95859	13.97418	0	0.839956482	0.840894608	0
603	arginyl-tRNA synthetase [Brevundimonas subvibrioides ATCC 15264]	gi|302383831	21.52000046	TDLRTALGEAVAAAFAAEGVDAALAR	18.20971	16.45507	17.32521	17.34038	0.903642617	0.951427013	0.952260085
604	SCO1/SenC [Nitrosomonas europaea ATCC 19718]	gi|30248691	7.409999847	DNASEN	18.45653	17.72091	17.38958	17.78004	0.960143104	0.942191192	0.963346848
605	transcriptional activator, Baf family [Gallionella capsiferriformans ES-2]	gi|302877832	11.39000034	ESGGA	16.59957	15.21793	15.58172	15.5975	0.916766519	0.938682147	0.939632774
606	CzcA family heavy metal efflux pump [Gallionella capsiferriformans ES-2]	gi|302878391	10.52999973	MVVVQSNVRDR	16.76603	14.65038	15.14705	15.63738	0.8738133	0.90343689	0.932682334
607	succinyl-CoA synthetase, alpha subunit [Gallionella capsiferriformans ES-2]	gi|302879758	28.78000069	TLLIGPNCPGLITPDEIK	17.26378	11.92857	16.16935	15.52132	0.690959338	0.936605425	0.899068454
608	hypothetical protein Galf_2601 [Gallionella capsiferriformans ES-2]	gi|302879798	18.13999939	FAGQMGTGSR	16.5488	15.60385	14.99354	15.67113	0.942899183	0.906019772	0.946964735

609	hypothetical protein HDN1F_02150 [gamma proteobacterium HdN1]	gi|304309866	20.13999939	AKIIGPIR	18.40035	0	16.66773	16.04231	0	0.905837661	0.87184809
610	hypothetical protein HDN1F_07770 [gamma proteobacterium HdN1]	gi|304310421	15.10999966	RKDSTTSGAEGNK	16.35262	15.55706	15.06506	15.44422	0.951349692	0.92126277	0.944449269
611	Phosphoenolpyruvate-protein phosphotransferase [Parvularcula bermudensis HTCC2503]	gi|304322088	11.27999973	LDRLATAIAANMVGDVCSIYVRR	16.41236	15.08083	15.28902	15.42136	0.91887029	0.931555243	0.939618678
612	hypothetical protein Dda3937_01230 [Dickeya dadantii 3937]	gi|307130660	8.220000267	GKKFA	16.34633	15.65058	0	15.57675	0.957436929	0	0.952920319
613	hypothetical protein Dda3937_04020 [Dickeya dadantii 3937]	gi|307131825	14.27000046	RHVCLLPPFLSRR	17.93098	14.91117	14.93752	15.80226	0.831587008	0.833056531	0.881282562
614	fimbrial transporter [Dickeya dadantii 3937]	gi|307132897	17.81999969	QGEGSEILAIDK	18.01653	17.51806	16.83132	16.61662	0.97233263	0.934215412	0.922298578
615	DNA segregation ATPase FtsK [Halomonas elongata DSM 2581]	gi|307545647	11.02000046	KSASHGR	16.95342	15.89128	15.85418	17.3861	0.937349514	0.935161165	1.025521694
616	camphor resistance protein CrcB [Halomonas elongata DSM 2581]	gi|307546836	5.190000057	AGTPS	16.65807	10.96134	14.90095	15.47084	0.658019807	0.894518393	0.928729439
617	three-deoxy-D-manno-octulosonic-acid transferase domain-containing protein [Sulfurimonas autotrophica DSM	gi|307721678	11.01000023	ISDKSVNKYMK	17.11543	15.48317	15.64112	0	0.904632253	0.913860768	0
618	Fertility inhibition FinO-like protein [Burkholderia sp. CCGE1003]	gi|307729847	2.029999971	AQSEPAAK	15.33513	14.34908	12.49968	14.38074	0.935699926	0.815101013	0.937764466
619	elongation factor P (EF-P) [Pantoea vagans C9-1]	gi|308187547	11.35999966	KGMAVTHNGKLLLVK	17.04777	0	13.68058	14.14403	0	0.802485017	0.829670391
620	ATP-dependent endonuclease of the old family [Pantoea vagans C9-1]	gi|308189216	17.72999954	APAAGMPS	18.18547	16.84785	16.7719	17.51557	0.926445673	0.922269262	0.963162899
621	hypothetical protein N47_A09530 [uncultured Desulfobacterium sp.]	gi|308270312	9.329999924	LYETFGVKPSTGQTKSK	15.48995	14.67022	13.89872	13.3798	0.947079881	0.897273393	0.863772962
622	hypothetical protein N47_H24770 [uncultured Desulfobacterium sp.]	gi|308271045	6.380000114	DLGMTAK	17.18029	16.30749	15.9575	16.45278	0.949197598	0.928825998	0.957654382
623	hypothetical protein N47_F15720 [uncultured Desulfobacterium sp.]	gi|308273274	28.84000015	LKAMKEIR	16.70715	13.46722	14.84992	15.77857	0.806075243	0.888836217	0.944420203
624	hypothetical protein N47_O12960 [uncultured Desulfobacterium sp.]	gi|308275281	12.89000034	RQTGNNP	17.60769	16.7482	16.42427	16.99846	0.951186669	0.932789594	0.965399777
625	DNA primase [Ketogulonicigenium vulgare Y25]	gi|310816222	10.23999977	ALIMTITDASIR	17.30381	14.72485	17.21476	16.27524	0.850959991	0.994853735	0.940558178
626	hypothetical protein STAUR_1078 [Stigmatella aurantiaca DW4/3-1]	gi|310818351	19.07999992	IKGGSALRAAAGVRYGGER	19.76971	17.28064	18.81407	0	0.874096787	0.951661405	0
627	short-chain dehydrogenase/reductase [Stigmatella aurantiaca DW4/3-1]	gi|310819657	10.05000019	MSFDLEMKDRR	16.63804	15.28737	15.25224	15.7406	0.918820366	0.916708939	0.946060954
628	hypothetical protein STAUR_5589 [Stigmatella aurantiaca DW4/3-1]	gi|310822823	9.539999962	DGNIVRVESITR	17.05259	0	15.48669	16.23789	0	0.908172307	0.952224266
629	adventurous gliding motility protein AgmK [Stigmatella aurantiaca DW4/3-1]	gi|310822882	5.739999771	APGDSMAQEAVK	14.29958	14.47429	13.19697	13.58103	1.012217841	0.922892141	0.949750272
630	helicase [Stigmatella aurantiaca DW4/3-1]	gi|310823354	11.63000011	SSVVIAELGPTNTGKTHRAIER	15.32444	13.94404	15.42036	14.75011	0.909921668	1.006259283	0.962521958
631	TRAP transporter solute receptor, TAXI family protein 2 [Achromobacter xylosoxidans A8]	gi|311103927	8.819999695	GVRDLKGKTIALGPEGSAVR	17.28907	16.18854	16.20977	16.47232	0.936345333	0.937573276	0.952759171
632	pyruvate kinase [Achromobacter xylosoxidans A8]	gi|311104082	14.39000034	ARMVREIAAKQGR	15.35507	15.22693	14.31938	14.67474	0.991654874	0.932550617	0.955693462
633	hypothetical protein AXYL_01653 [Achromobacter xylosoxidans A8]	gi|311104853	4.349999905	MTGAA	16.77896	16.12101	15.24793	15.90071	0.9607872	0.908752986	0.947657662
634	iron uptake receptor [Achromobacter xylosoxidans A8]	gi|311108093	24.51000023	ELNVDAGSFDR	17.08557	15.59933	0	17.55071	0.913011974	0	1.027224143
635	hypothetical protein Rvan_0456 [Rhodomicrobium vannielii ATCC 17100]	gi|312113241	13.02000046	LALQAIGPK	16.64071	15.16415	15.25616	15.44664	0.911268209	0.91679742	0.928244047
636	hypothetical protein Rvan_0538 [Rhodomicrobium vannielii ATCC 17100]	gi|312113322	15.14000034	AVYEKKPTNMR	17.0767	16.08182	16.72138	0	0.9417405	0.979192701	0
637	phenylalanyl-tRNA synthetase subunit alpha [Rhodomicrobium vannielii ATCC 17100]	gi|312113593	20.90999985	TNPAAEGR	16.52006	0	15.39749	15.42654	0	0.932048068	0.933806536
638	hypothetical protein Rvan_2584 [Rhodomicrobium vannielii ATCC 17100]	gi|312115302	16	GVSYDPK	17.0579	14.04303	14.32318	0	0.823256673	0.839680148	0
639	glutamine-dependent NAD(+) synthetase [Burkholderia rhizoxinica HKI 454]	gi|312795378	20.12000084	YTADMSMSDAAEMARRVGVR	17.4146	15.86231	15.39069	15.86029	0.910862724	0.883780851	0.91074673
640	Dimethyladenosine transferase [Burkholderia rhizoxinica HKI 454]	gi|312797254	11.10999966	DLIARLQQRFGSR	16.64859	16.33457	15.42971	15.34881	0.981138343	0.926787794	0.921928524
641	hypothetical protein Sulku_2813 [Sulfuricurvum kujiense DSM 16994]	gi|313669589	15.02999973	VSSTSLIYKNFIK	15.29918	14.71384	14.15967	14.53248	0.961740433	0.92551823	0.949886203
642	site-specific tyrosine recombinase XerD [Candidatus Liberibacter solanacearum CLso-ZC1]	gi|315121780	9.680000305	MFLLIELLYSTGMR	16.68173	15.36005	16.28278	15.63451	0.920770807	0.976084615	0.937223537
643	U1 small nuclear ribonucleoprotein [Helicobacter felis ATCC 49179]	gi|315452869	6.199999809	MAHGGK	16.42562	14.24373	14.72533	15.96487	0.86716544	0.89648549	0.971949308
644	adenine-specific DNA-methyltransferase [Helicobacter felis ATCC 49179]	gi|315453673	16.04999924	NGGSGKNYPDLKLLLNTPSARK	17.64446	16.37021	16.77596	17.02934	0.927781865	0.950777751	0.965138066
645	hypothetical protein Astex_0372 [Asticcacaulis excentricus CB 48]	gi|315497415	16.04000092	RKGRPKSMSGQGR	17.59971	16.44057	15.79912	16.37124	0.934138688	0.897692064	0.930199418
646	hypothetical protein Astex_1306 [Asticcacaulis excentricus CB 48]	gi|315498326	10.18999958	AGGGTDK	14.82274	16.10879	13.53543	14.68801	1.086761962	0.913153034	0.990910587
647	nitrite reductase [Rhodopseudomonas palustris DX-1]	gi|316935652	16.97999954	IYYIGEQDMYVPK	16.17662	14.71044	14.93441	15.16802	0.909364255	0.923209546	0.937650758
648	glycosyl transferase group 1 [Pantoea sp. At-9b]	gi|317054343	16.73999977	ALPEINPPFAGWLIDHK	17.55435	14.97871	0	16.52831	0.853276253	0	0.94155067
649	response regulator receiver [Desulfovibrio aespoeensis Aspo-2]	gi|317152722	6.909999847	SGGSQS	16.23217	15.08235	14.93512	15.48398	0.929164123	0.920093863	0.953906964
650	hypothetical protein Daes_2420 [Desulfovibrio aespoeensis Aspo-2]	gi|317154123	19.06999969	FDEIRDLLADADLQMER	0	17.37515	16.87269	16.8666		0.971081689	0.970731188
651	hypothetical protein BARRO_10265 [Bartonella rochalimae ATCC BAA-1498]	gi|319403747	17.39999962	VTNETEEAQKR	18.08266	17.03859	16.81231	17.51508	0.94226126	0.929747615	0.968611919
652	Chlorite dismutase [Alicycliphilus denitrificans BC]	gi|319760122	1.340000033	TLSMGN	16.94794	14.84784	0	15.91481	0.876085235	0	0.939040969
653	3-hydroxyisobutyrate dehydrogenase [Alicycliphilus denitrificans BC]	gi|319761008	11.06000042	AASVQALAAGADVVFTMLPGPK	18.67801	14.84798	16.98146	0	0.794944429	0.909168589	0
654	phage major tail tube protein [Alicycliphilus denitrificans BC]	gi|319761973	9.420000076	KMEDYRSGGMNAPVK	16.46726	13.21852	14.65524	16.2446	0.802715206	0.889962265	0.986478625
655	acyltransferase [Alicycliphilus denitrificans BC]	gi|319763115	16.19000053	TAPRGGLWRR	16.05444	15.09154	14.99852	15.29835	0.940022822	0.934228787	0.952904617
656	porin [Alicycliphilus denitrificans BC]	gi|319764446	7.630000114	AAGTNDK	15.34363	13.35083	13.98017	14.38625	0.870121999	0.911138368	0.937604074
657	leucyl-tRNA synthetase [Alicycliphilus denitrificans BC]	gi|319764765	10.68000031	DVEFGKEAR	16.38717	15.02706	14.7796	15.12128	0.917001532	0.901900694	0.922751152
658	dihydroxy-acid dehydratase [Taylorella equigenitalis MCE9]	gi|319778973	17.23999977	TSTHGNQMAGARALWRATGVK	18.19911	16.67994	17.41863	17.30567	0.916525039	0.957114386	0.950907489
659	NADH:flavin oxidoreductase [Mesorhizobium ciceri biovar biserrulae WSM1271]	gi|319779830	12.88000011	QMKGETT	16.77687	15.09882	14.69975	15.32562	0.899978363	0.876191447	0.913496975
660	oxidoreductase molybdopterin binding protein [Mesorhizobium ciceri biovar biserrulae WSM1271]	gi|319780154	10.85000038	GFSSGR	16.24411	14.01334	14.1836	15.44094	0.86267207	0.873153408	0.950556232
661	polar amino acid ABC transporter inner membrane subunit [Mesorhizobium ciceri biovar biserrulae WSM1271	gi|319782302	3.339999914	STVGS	0	16.67409	15.98318	16.52059		0.958563856	0.9907941
662	alpha-2-macroglobulin [Mesorhizobium ciceri biovar biserrulae WSM1271]	gi|319782770	12.92000008	VIDHTIDADTSAPR	16.69575	13.54869	15.3055	15.90858	0.811505323	0.916730306	0.952852073
663	PAS sensor protein [Mesorhizobium ciceri biovar biserrulae WSM1271]	gi|319785036	2.789999962	AIGAS	17.07331	16.12779	15.879	16.39604	0.944619995	0.930048128	0.960331652
664	uvrd/rep helicase [Variovorax paradoxus EPS]	gi|319793174	10.69999981	ALGEALTK	16.35149	15.48676	15.10045	15.58616	0.947116134	0.923490764	0.953195091
665	tonb-dependent siderophore receptor [Variovorax paradoxus EPS]	gi|319794503	16.20999908	TDGFMVR	16.5719	15.64856	14.95986	15.83969	0.944282792	0.902724491	0.955816171
666	alpha/beta hydrolase fold protein [Variovorax paradoxus EPS]	gi|319795647	1.519999981	TFPSA	17.65132	15.59083	15.07708	15.53787	0.883267087	0.854161615	0.880266745
667	seryl-tRNA synthetase [Nitratifractor salsuginis DSM 16511]	gi|319956131	13.52999973	DTRGMIRQHQFAK	0	17.85478	17.58216	16.56961		0.98473126	0.928020956
668	hypothetical protein Nitsa_1242 [Nitratifractor salsuginis DSM 16511]	gi|319956981	9.340000153	RGYSAKAASELVGNR	17.33289	16.67081	16.27141	15.84065	0.9618021	0.938759203	0.913907029
669	electron transport complex protein RnfC [Vibrio vulnificus MO6-24/O]	gi|320156874	8.640000343	RAVMDGEPMVNR	17.34725	15.09368	16.79406	16.3185	0.870090648	0.968110796	0.940696652

670	flagellin domain-containing protein [Desulfobulbus propionicus DSM 2032]	gi|320354261	14.26000023	TIAEKINNISNDTGVNATAVTNAK	17.59605	0	16.19636	16.01119	0	0.920454307	0.909930922
671	iron-containing alcohol dehydrogenase [Desulfobulbus propionicus DSM 2032]	gi|320354833	15.36999989	GRALEGRLPMEK	18.67615	16.13209	0	17.41627	0.863780276	0	0.9325407
672	disease-specific protein E [Erwinia amylovora]	gi|321689678	13.82999992	RRNMDDMAGRPMVKGGSGEDK	17.88017	18.12753	16.86902	17.00338	1.01383432	0.943448524	0.950962994
673	cytochrome c [Geobacter sp. M18]	gi|322420215	30.04000092	YDWNKAIAAGMKAAGLPYSGK	19.17203	17.38639	18.00059	18.4139	0.906862236	0.938898489	0.960456457
674	DNA mismatch repair protein MutL [Geobacter sp. M18]	gi|322421005	18.76000023	AEAATASLR	16.38182	15.51542	0	15.61923	0.947112104	0	0.953449006
675	family 3 extracellular solute-binding protein [Burkholderia sp. CCGE1001]	gi|323529851	16.43000031	ALIGAMLGAAAILAAPAQAK	18.05577	16.76069	16.82593	0	0.928273344	0.931886594	0
676	luciferase-like protein [Burkholderia sp. CCGE1001]	gi|323530062	15.47999954	YDDTTSHR	16.41432	15.85435	15.41183	15.36734	0.965885276	0.938925889	0.936215451
677	NADPH:quinone reductase [Agrobacterium sp. H13-3]	gi|325168820	19.62999916	RAAFYER	15.55479	14.94895	14.64468	15.09464	0.961051226	0.941490049	0.970417473
678	binding-protein-dependent transport systems inner membrane component [Burkholderia sp. TJI49]	gi|325529843	5.739999771	VTLPV	17.07969	15.69558	16.02488	16.35993	0.918961644	0.938241853	0.95785872
679	hypothetical protein NAL212_3089 [Nitrosomonas sp. AL212]	gi|325983611	3.029999971	MMGHSK	16.77808	15.5341	15.35054	15.03286	0.925856832	0.914916367	0.895982139
680	type II secretory pathway protein D [Acidiphilium multivorum AIU301]	gi|326403170	34.33000183	IIADPRQNAILVR	18.35116	16.92136	16.50353	15.95754	0.922086669	0.899318081	0.869565739
681	alanine--glyoxylate transaminase [Marinomonas mediterranea MMB-1]	gi|326794952	18.55999947	HGSTLK	16.99215	15.98225	15.19357	16.21683	0.940566673	0.8941523	0.954371872
682	hypothetical protein Marme_1781 [Marinomonas mediterranea MMB-1]	gi|326795053	18.40999985	ATEQLSSYEMMGVDPLR	17.50842	16.01813	16.31283	17.45005	0.914881526	0.931713427	0.996666175
683	protein recA [Hippea maritima DSM 10411]	gi|327398415	17.03000069	KSGSPIK	16.92141	15.84635	10.09353	15.91703	0.936467469	0.596494618	0.940644426
684	nucleoside triphosphate hydrolase [Polymorphum gilvum SL003B-26A1]	gi|328543132	11.15999985	SPGPGRPGSLRAMMERR	17.13046	14.32322	15.10653	15.8931	0.836125825	0.881851976	0.927768431
685	hypothetical protein SL003B_3027 [Polymorphum gilvum SL003B-26A1]	gi|328544645	17.09000015	LGRTATR	17.48988	17.08913	16.36162	16.82011	0.97708675	0.935490695	0.961705283
686	peptidase S58 DmpA [Polymorphum gilvum SL003B-26A1]	gi|328544724	17.19000053	QVAIDGK	17.62253	16.89896	16.38306	16.71502	0.958940629	0.929665604	0.948502854
687	multi-sensor hybrid histidine kinase [Desulfobacca acetoxidans DSM 11109]	gi|328952225	15.30000019	EMGNGTGLGLSTVYGIVK	17.62495	16.02445	16.36835	17.22538	0.909191232	0.928703344	0.977329297
688	glycyl-tRNA synthetase subunit beta [Desulfobacca acetoxidans DSM 11109]	gi|328952742	10.42000008	HAAGRPAHER	16.70074	15.65497	15.50093	16.22842	0.937381817	0.928158273	0.971718618
689	hypothetical protein Desac_2072 [Desulfobacca acetoxidans DSM 11109]	gi|328953748	10.64999962	TDFLAIAAVRWPACL	17.65462	14.65123	16.46414	16.15365	0.82988079	0.932568359	0.914981461
690	NGG1p interacting factor 3 protein, NIF3 [Desulfobacca acetoxidans DSM 11109]	gi|328954513	18	VAGRTPSK	17.14734	15.9347	16.20226	16.39836	0.92928116	0.944884746	0.956320922
691	heavy metal translocating P-type ATPase [Pseudomonas mendocina NK-01]	gi|330503010	18.89999962	LSRTPNM	16.055	16.65975	15.10682	15.32506	1.037667393	0.940941763	0.954535036
692	hypothetical protein [Pseudomonas mendocina NK-01]	gi|330503400	7.019999981	FTPTSKVMLLSAEGR	17.63231	13.73241	15.12159	15.51685	0.778820812	0.857606859	0.880023661
693	SM-20-related protein [gamma proteobacterium IMCC2047]	gi|330720385	8.850000381	VDPPT	16.23091	13.93562	15.90569	16.81616	0.858585255	0.979962923	1.036057744
694	BNR/Asp-box repeat protein [gamma proteobacterium IMCC2047]	gi|330720539	16.48999977	EVHSAVMVK	17.64149	15.47164	16.05264	16.79767	0.87700302	0.909936746	0.952168439
695	toxin [Pseudomonas brassicacearum subsp. brassicacearum NFM421]	gi|330807393	10.26000023	NQLDSVLLVQREDR	19.0921	15.47301	15.88063	15.20967	0.810440444	0.831790636	0.796647304
696	hypothetical protein PSEBR_a4464 [Pseudomonas brassicacearum subsp. brassicacearum NFM421]	gi|330811417	32.13000107	LRLLLGEHRGR	16.94754	15.58974	15.7318	13.86182	0.919882178	0.928264515	0.817925197
697	hypothetical protein PSEBR_a4932 [Pseudomonas brassicacearum subsp. brassicacearum NFM421]	gi|330811896	10.11999989	GYQVTY	16.45346	15.24564	15.20961	15.51361	0.926591732	0.924401919	0.942878276
698	hypothetical protein bgla_1g30360 [Burkholderia gladioli BSR3]	gi|330817898	7.840000153	EPAEGGPR	16.82169	13.54442	13.50032	14.37921	0.805175937	0.802554321	0.854801747
699	helix-turn-helix domain protein [Burkholderia gladioli BSR3]	gi|330819542	5.929999828	PAAGPR	15.95894	15.20933	14.47781	15.17742	0.953028835	0.907191204	0.951029329
700	hypothetical protein bgla_2g15750 [Burkholderia gladioli BSR3]	gi|330820670	9.890000343	NAGKAMEAIEIGK	16.71321	14.73043	15.96676	15.45902	0.881364501	0.955337724	0.924958162
701	coenzyme A transferase domain-containing protein [Burkholderia gladioli BSR3]	gi|330821683	10.56999969	AEPMGIAK	16.61576	15.56857	15.34045	15.887	0.9369761	0.923246966	0.956140435
702	2,4-dihydroxyhept-2-ene-1,7-dioic acid aldolase [Alicycliphilus denitrificans K601]	gi|330823148	14.03999996	GMGGTMR	17.95631	17.42665	16.76939	17.19439	0.970502848	0.93389956	0.95756812
703	type IV secretion system protein TraD [Alicycliphilus denitrificans K601]	gi|330827327	7.78000021	EEKGSAGSFSR	16.3399	14.22756	15.27314	15.58895	0.870725035	0.934714411	0.954041946
704	polyphosphate kinase [Aeromonas veronii B565]	gi|330830293	45.24000168	LIDNELSNAKAGQPSGIILK	19.80416	17.31795	17.63529	18.65268	0.874460214	0.890484121	0.94185666
705	hypothetical protein B565_3425 [Aeromonas veronii B565]	gi|330831125	16.04999924	EMAGPLARLVKVR	16.7174	15.49406	15.42079	15.18826	0.926822353	0.922439494	0.908530035
706	hypothetical protein Pgy4_39765 [Pseudomonas savastanoi pv. glycinea str. race 4]	gi|330884972	0.519999981	TAGDL	16.70787	15.55269	15.13787	15.57687	0.930860128	0.906032307	0.93230735
707	secretion protein HlyD, partial [Pseudomonas syringae pv. japonica str. M301072]	gi|330897580	14.65999985	ISMTAQVHIVR	16.33572	16.89319	15.14642	15.32515	1.03412583	0.927196353	0.938137407
708	hypothetical protein PSYJA_11520 [Pseudomonas syringae pv. japonica str. M301072]	gi|330898145	12	KALDDKVANSR	15.70212	14.44206	14.46204	15.02892	0.919752237	0.921024677	0.957126808
709	Rhs element Vgr protein [Pseudomonas syringae pv. japonica str. M301072]	gi|330901448	10.55000019	EFQAGKISVQTFDYRQPGNR	17.93637	16.06208	16.6705	14.61863	0.895503382	0.929424404	0.815027232
710	hypothetical protein PSYPI_14563 [Pseudomonas syringae pv. pisi str. 1704B]	gi|330940442	3.829999924	ESSGA	16.66577	15.97549	15.1639	15.33334	0.958580972	0.909882952	0.920049899
711	hypothetical protein PSYPI_17772 [Pseudomonas syringae pv. pisi str. 1704B]	gi|330941272	20.61000061	KSAPLT	18.85208	10.66815	15.59241	18.83329	0.565887159	0.827092289	0.999003293
712	ATPase AAA [Alteromonas macleodii str. 'Deep ecotype']	gi|332141044	19.11000061	ESNLPSGK	16.64542	17.75502	16.95278	14.43376	1.066660979	1.018465139	0.867131019
713	murein transglycosylase [Alteromonas macleodii str. 'Deep ecotype']	gi|332141406	1.549999952	SSASG	16.27533	15.84681	15.33771	15.27761	0.97367058	0.942390108	0.938697403
714	hypothetical protein YE105_C1359 [Yersinia enterocolitica subsp. palearctica 105.5R(r)]	gi|332160981	17.45000076	DTDHKK	18.11021	15.29819	16.89419	16.18605	0.844727366	0.932854451	0.893752751
715	DNA topoisomerase III [Pusillimonas sp. T7-7]	gi|332286274	8.489999771	VQTPTLAIVNER	16.99992	16.1741	16.88645	16.01697	0.951422124	0.993325263	0.94217914
716	putative hemolysin-type protein [Gallibacterium anatis UMN179]	gi|332289410	18.55999947	GIIDGSNR	16.08428	15.5914	15.29275	14.75725	0.969356415	0.950788596	0.917495219
717	hypothetical protein Glaag_3893 [Glaciecola sp. 4H-3-7+YE-5]	gi|332308236	26.79999924	DTAKKKGK	18.92403	17.7141	16.7966	18.15906	0.93606383	0.8875805	0.959576792
718	putative transglycosylase [Alteromonas sp. SN2]	gi|333892432	14.64000034	ASLLMVAGVLASIGFAQAEDK	17.82911	15.87184	17.2652	18.14029	0.890220544	0.968371388	1.017453479
719	putative TonB dependent receptor [Alteromonas sp. SN2]	gi|333892825	9.029999733	WDQYSAQLDYGTAIEEGKSGVR	16.17525	14.05207	13.91033	14.60326	0.868738968	0.859976198	0.902815103
720	ABC transporter ATP-binding protein [Alteromonas sp. SN2]	gi|333893803	20.62000084	LSTIVDADTILVLSK	16.8182	16.22132	15.75155	16.43552	0.964509876	0.936577636	0.977246079
721	hypothetical protein Psefu_1459 [Pseudomonas fulva 12-X]	gi|333899656	18.79000092	EVSDGLDAMANDGR	17.03879	16.44263	15.72999	16.24343	0.9650116	0.923187034	0.953320629
722	glucosylglycerol 3-phosphatase [Pseudomonas fulva 12-X]	gi|333901133	22.37000084	ERLRPAQGAAAGTTDLQLMLR	20.12775	15.77935	16.36805	15.56196	0.783959956	0.813208133	0.773159444
723	hypothetical protein Psefu_3409 [Pseudomonas fulva 12-X]	gi|333901592	9.079999924	NIGKNAK	15.65691	14.94292	15.04949	13.74458	0.954397771	0.96120435	0.877860319
724	MotA/TolQ/ExbB proton channel [Marinomonas posidonica IVIA-Po-181]	gi|333908209	12.19999981	GMQSKTGLASIFAAGLVSSKHGR	17.40623	16.25653	16.06385	0	0.933948937	0.922879337	0
725	winged helix family two component transcriptional regulator [Marinomonas posidonica IVIA-Po-181]	gi|333909630	16.86000061	FIMPIMMLTGK	17.70213	14.80145	15.05413	15.38516	0.836139493	0.850413481	0.869113491
726	Pyruvate decarboxylase [Methylomonas methanica MC09]	gi|333985389	13.76000023	LETMGQYLLNR	18.32924	15.65731	16.68709	16.89283	0.854225816	0.910408178	0.921632866
727	group 1 glycosyl transferase [Novosphingobium sp. PP1Y]	gi|334140556	1.889999986	GADLAIK	0	14.87211	14.87598	15.2691		1.000260219	1.02669359
728	MFS permease [Novosphingobium sp. PP1Y]	gi|334140591	15.85000038	SMFGLLAQEPVLRR	17.58698	0	15.6645	17.19643	0	0.890687315	0.977793231
729	DNA gyrase subunit A [Novosphingobium sp. PP1Y]	gi|334142014	12.68999958	SARIVGDTMGK	17.23479	15.40763	15.69919	16.43793	0.893984203	0.910901148	0.95376445
730	UTP-GlnB uridylyltransferase, GlnD [Thioalkalimicrobium cyclicum ALM1]	gi|334144063	14.68999958	ALFIAIFR	16.74876	15.23573	15.14955	15.63279	0.909663163	0.904517708	0.933369993

731	septum site-determining protein minC [Thioalkalimicrobium cyclicum ALM1]	gi|334144174	16.95000076	DKPKPDAIATAEAAQPAEAISR	0	16.11193	15.58386	16.03891		0.967224907	0.995467954
732	UDP-N-acetylenolpyruvoylglucosamine reductase [Thioalkalimicrobium cyclicum ALM1]	gi|334144210	8.43999958	HALVLVNLGGASGSDVLK	15.32735	15.13976	0	14.7457	0.987761094	0	0.962051496
733	tartrate dehydrogenase [Novosphingobium sp. PP1Y]	gi|334145108	8.100000381	AVASLIG	17.93143	17.15158	16.71099	17.25489	0.956509325	0.931938501	0.962270717
734	hypothetical protein Sinme_1059 [Sinorhizobium meliloti AK83]	gi|334315805	6.989999771	DVSELHADVRTRER	16.11006	13.79745	13.31601	14.54685	0.856449324	0.826564892	0.902966842
735	transposase IS4 family protein [Sinorhizobium meliloti AK83]	gi|334317360	11.57999992	SGLILDLVIEAGNPADSERLLPLLER	15.88629	0	14.63078	14.35752	0	0.920968961	0.903767966
736	hypothetical protein Sinme_5288 [Sinorhizobium meliloti AK83]	gi|334318468	22.52000046	GGTFVTAR	19.18309	17.88691	17.87581	18.49964	0.932431115	0.93185248	0.964372267
737	LytTR family two component transcriptional regulator [Sinorhizobium meliloti AK83]	gi|334320641	8.710000038	VGTVPDG	16.8584	0	15.49898	15.73017	0	0.919362454	0.933076093
738	isochorismatase hydrolase [Sphingobium chlorophenolicum L-1]	gi|334343189	16.97999954	KWGGAYGLGITYAKTMFNASEGTH	17.53078	15.15997	16.02639	16.34499	0.864763005	0.914185792	0.932359541
739	regulatory protein TetR [Sphingobium chlorophenolicum L-1]	gi|334344258	11.94999981	RDMGMPEPPSPVG	16.28298	15.00085	14.92402	0	0.921259499	0.916541075	0
740	hypothetical protein Sphch_0921 [Sphingobium chlorophenolicum L-1]	gi|334344566	14.28999996	RSRKFGAYANSDLFLAMVTR	18.34278	16.95062	16.61756	16.52414	0.924103108	0.905945555	0.900852543
741	preprotein translocase subunit SecY [Sphingobium chlorophenolicum L-1]	gi|334345447	9.5	QTNGGILDIFNTFSGGSLSR	16.0606	12.39268	14.72961	16.15249	0.771619989	0.917127006	1.005721455
742	sugar transporter [Sphingobium chlorophenolicum L-1]	gi|334345501	22.39999962	ILNAVIDPLVGLWIDGR	17.5457	14.88475	14.82286	15.28018	0.848341759	0.844814399	0.870878905
743	transmembrane transport protein [Bordetella pertussis Tohama I]	gi|33593068	15.19999981	IPETTPAPAIA	16.46898	14.41222	15.69442	15.68943	0.875113091	0.952968551	0.952665557
744	tRNA 2-methylthioadenosine synthase [Vibrio anguillarum 775]	gi|336124702	14.27000046	TEQEMDLRTVISPTQMMAKTR	16.67528	15.4837	15.69582	16.4048	0.928542129	0.941262755	0.983779583
745	hypothetical protein Mesop_0991 [Mesorhizobium opportunistum WSM2075]	gi|337265516	20.62999916	NTPMRAK	19.96588	13.02293	17.27214	16.39005	0.652259254	0.865082831	0.82090296
746	hypothetical protein Mesop_3740 [Mesorhizobium opportunistum WSM2075]	gi|337268220	15.81999969	AQQDKADAAMK	17.76497	16.70383	16.02539	16.83656	0.940267842	0.902078078	0.947739287
747	NAD-dependent epimerase/dehydratase [Mesorhizobium opportunistum WSM2075]	gi|337268313	16.77000046	QKTEELAAVWRMR	16.75265	16.85238	15.11427	15.23517	1.005953088	0.902201741	0.90941851
748	hypothetical protein Rta_10310 [Ramlibacter tataouinensis TTB310]	gi|337278664	14.10000038	AMAAAHGR	18.34195	17.20426	17.01154	17.38404	0.937973334	0.927466273	0.947774909
749	hypothetical protein Rta_19260 [Ramlibacter tataouinensis TTB310]	gi|337279564	15.31000042	SPEAALATAEAMAR	17.03026	15.97876	15.81561	16.46473	0.938256961	0.928676955	0.966792639
750	hypothetical protein Rta_27650 [Ramlibacter tataouinensis TTB310]	gi|337280415	13.51000023	GMPVDTR	17.47861	15.66614	15.46353	15.76052	0.896303539	0.884711656	0.901703282
751	hypothetical protein F7308_1282 [Francisella sp. TX077308]	gi|337755297	12.81999969	LKAIHLEHSDR	16.63081	15.13547	15.49551	16.7907	0.910086159	0.931735135	1.009614084
752	hypothetical protein LILAB_03850 [Myxococcus fulvus HW-1]	gi|338530439	19.5	KAPARADVPR	17.16769	16.09102	16.11223	16.75307	0.937285098	0.938520558	0.975848818
753	ATP-dependent DNA helicase UvrD [Myxococcus fulvus HW-1]	gi|338533797	13.14000034	AELDAAIARCK	16.72698	16.51342	15.66646	16.0249	0.987232603	0.936598238	0.958027092
754	HAD superfamily hydrolase [Myxococcus fulvus HW-1]	gi|338534246	9.18999958	YRVFPGMREAVKEAR	16.27087	15.03557	15.5951	16.11171	0.924079044	0.958467494	0.990218101
755	thiamine pyrophosphate protein [Myxococcus fulvus HW-1]	gi|338535567	11.5	RLYGYPGDGINGIVGALGR	18.55791	0	17.19657	16.77675	0	0.926643679	0.90402152
756	cobalamin biosynthesis protein cobD [Hyphomicrobium sp. MC1]	gi|338737530	8.489999771	LDDFVNLPASR	0	16.40786	15.97767	16.43315		0.973781468	1.001541334
757	hypothetical protein HYPMC_1684 [Hyphomicrobium sp. MC1]	gi|338738524	20.75	NAFLA	16.76555	15.65014	15.74788	15.89827	0.933470122	0.939299933	0.948270113
758	hypothetical protein HYPMC_2462 [Hyphomicrobium sp. MC1]	gi|338739285	16.71999931	MAEVESSR	14.03281	13.96312	13.22087	13.9262	0.995033782	0.942139885	0.992402805
759	hypothetical protein HYPMC_4389 [Hyphomicrobium sp. MC1]	gi|338741200	12.65999985	IRLRVLDNDIHIKEMK	16.0117	15.36055	13.70586	16.04684	0.959332863	0.855990307	1.002194645
760	granule protein PhaP [Cupriavidus necator N-1]	gi|339325543	83.98000336	QAVEIAETNFQAAATAATK	19.20141	16.54127	0	17.98736	0.861461216	0	0.936772872
761	lipid-A-disaccharide synthase [Cupriavidus necator N-1]	gi|339326108	5.059999943	GGLPTGNGSAADQR	17.51841	15.11236	15.33248	16.48107	0.862655915	0.875220982	0.940785722
762	isopropylmalate isomerase large subunit [Chromobacterium violaceum ATCC 12472]	gi|34498239	24.95000076	LGAGITAK	17.75278	16.84412	16.46114	16.99255	0.948815904	0.927242944	0.957176848
763	hypothetical protein CV_2817 [Chromobacterium violaceum ATCC 12472]	gi|34498272	15.55000019	DGVERLAQAAELMSR	0	17.63887	17.22212	16.6829		0.976373203	0.945803218
764	acetyl-CoA synthetase [Chromobacterium violaceum ATCC 12472]	gi|34499131	23.57999992	EMALAMTRDGEGGEEMLAVAR	18.85106	17.10648	17.29965	17.58252	0.907454541	0.91770171	0.932707232
765	Ferredoxin--NAD(+) reductase [Sinorhizobium fredii NGR234]	gi|36958767	20.25	VAAEPLSR	16.53493	16.41328	14.54373	15.46629	0.992642848	0.879576146	0.935370758
766	Ribose transport system permease protein rbsC [Sinorhizobium fredii NGR234]	gi|36958938	17.09000015	MSFAIGIDEK	17.70776	16.02	16.8858	16.96234	0.904688114	0.953581932	0.957904331
767	putative membrane transport protein [Bacteriovorax marinus SJ]	gi|374287275	12.36999989	ALPMGSICMTGLAVALFTGTLKLK	15.69698	17.11948	13.15669	15.12108	1.090622527	0.83816696	0.963311414
768	hypothetical protein [Desulfovibrio africanus str. Walvis Bay]	gi|374298644	11.47999954	MLNHCTGELK	16.51291	14.88504	14.545	14.93968	0.901418345	0.880825972	0.904727271
769	hypothetical protein [Desulfovibrio africanus str. Walvis Bay]	gi|374301147	13.64999962	ATDGKP	17.64346	16.71419	16.29647	16.72028	0.947330626	0.923654997	0.947675796
770	NLP/P60 protein [Desulfovibrio africanus str. Walvis Bay]	gi|374301201	9.390000343	DTALTSADK	17.50842	15.77447	15.91807	0	0.900964793	0.909166561	0
771	ATP-dependent dsDNA exonuclease [Acinetobacter calcoaceticus PHEA-2]	gi|375133781	16.88999939	QIDEVDQNLKEITLKGQQNNEK	16.0217	13.53759	13.22895	13.85795	0.844953407	0.825689534	0.864948788
772	hypothetical protein BAA13334_II00299 [Brucella abortus A13334]	gi|376270605	9.050000191	RSSSCAAGLFIGMR	17.52872	16.39554	15.80962	15.76609	0.935352952	0.901926667	0.899443314
773	hypothetical protein DND132_0443 [Desulfovibrio desulfuricans ND132]	gi|376295234	11.47999954	VLGQSDRTITTLMSPPKGTPR	18.53759	0	17.61895	16.90424	0	0.950444475	0.911889841
774	Mg chelatase subunit ChlI [Desulfovibrio desulfuricans ND132]	gi|376297078	12.59000015	IATISCAALMGIDAFK	18.37025	16.10363	16.5502	17.04077	0.876614635	0.900924048	0.927628639
775	protein YhiI [Pasteurella multocida subsp. multocida str. HN06]	gi|383310937	9.819999695	KNQLISSSEFEKRLALR	0	15.6701	16.04689	16.42474		1.024045156	1.048157957
776	methyltransferase [Zymomonas mobilis subsp. mobilis ATCC 10988]	gi|384412021	18.23999977	SNIEKMGAQADTR	20.12992	16.16164	17.82649	17.9881	0.802866579	0.885571825	0.893600173
777	peptidyl-tRNA hydrolase [Helicobacter pylori v225d]	gi|384890082	4.070000172	KPLTI	18.40949	17.79773	17.40022	16.87787	0.966769313	0.945176645	0.916802693
778	putative aldehyde dehydrogenase subunit III, partial [Sulfitobacter sp. EE-36]	gi|38490096	24.76000023	HAVQEAWIDLQ	16.5175	14.84605	15.75269	15.46676	0.898807326	0.953696988	0.936386257
779	cation/multidrug efflux pump [Acinetobacter baumannii TCDC-AB0715]	gi|385237584	9.829999924	IMATEGLSPK	16.71497	15.52992	15.46869	15.83061	0.929102475	0.925439292	0.947091739
780	bifunctional protein PyrR [Marinobacter adhaerens HP15]	gi|385333249	10.88000011	TMALESHQR	15.22258	14.90398	14.89892	14.72519	0.979070565	0.978738164	0.967325512
781	insertion element transposase [Neisseria meningitidis NZ-05/33]	gi|385858047	21.45999908	MEMFDGKVEADESYFGGQR	17.5743	15.81155	15.51754	0	0.899697285	0.882967743	0
782	putative flavin-binding monooxygenase [Pseudomonas aeruginosa NCGM2.S1]	gi|386066006	14.82999992	MLAFATFPALMKVMRLSFRR	17.79938	14.62816	14.87516	15.13707	0.821835367	0.835712255	0.850426813
783	hypothetical protein [Shewanella baltica BA175]	gi|386322434	13.47000027	IHPMRLSGGNFSSGYNR	16.8487	0	15.40083	14.55386	0	0.914066367	0.863797207
784	hypothetical protein RSPO_c01813 [Ralstonia solanacearum Po82]	gi|386333479	17.56999969	DDPPAGR	17.12294	16.39238	16.09148	16.44896	0.95733443	0.939761513	0.960638769
785	hypothetical protein RSPO_c02970 [Ralstonia solanacearum Po82]	gi|386334627	11.42000008	RLLADHPAR	16.41041	15.11568	15.5273	15.74161	0.921103129	0.946185988	0.959245381
786	glutamate synthase subunit alpha [Escherichia coli UM146]	gi|386602704	3.960000038	SAAGV	17.4321	16.77611	16.31465	16.30183	0.962368848	0.935896995	0.93516157
787	hypothetical protein UMNK88_3686 [Escherichia coli UMNK88]	gi|386615719	17.72999954	GSTANIAMAK	15.71627	14.91677	14.4338	15.03101	0.949129151	0.918398577	0.956398051
788	serine acetyltransferase [Providencia stuartii MRSN 2154]	gi|386743544	15.23999977	LSSPIMPAMEVR	17.31878	14.94658	15.47888	16.36151	0.863027303	0.893762725	0.944726476
789	hypothetical protein Deval_1293 [Desulfovibrio vulgaris RCH1]	gi|387153155	15.56000042	LIAVIRDTLR	16.50534	15.95909	15.1949	15.66917	0.96690465	0.920605089	0.949339426
790	hypothetical protein FN3523_1484 [Francisella cf. novicida 3523]	gi|387825067	9.130000114	IVASCSQDKGFWSK	17.22781	14.11358	14.2236	14.54006	0.819232392	0.825618578	0.843987715
791	hypothetical protein RPA4548 [Rhodopseudomonas palustris CGA009]	gi|39937606	10.06000042	RLTCVNSKPVYEAAK	18.11234	14.99865	15.29285	15.96018	0.828090131	0.8443332	0.881177142

792	glycosyltransferase [Pelobacter carbinolicus DSM 2380]	gi|404492729	6.070000172	ATGCDVVIAPAK	16.17345	14.8896	14.3096	14.71836	0.920619905	0.884758663	0.910032182
793	subtilase family serine protease [Geobacter metallireducens GS-15]	gi|404495792	6.199999809	KKGMPK	16.73539	15.6314	15.38256	15.7816	0.93403261	0.919163521	0.943007602
794	glycerol-3-phosphate dehydrogenase [Geobacter metallireducens GS-15]	gi|404496538	14.71000004	GGVIYHDGQFDDARLAVALAR	17.13031	13.95467	17.02159	16.32689	0.81461865	0.993653355	0.953099506
795	carbamoyl-phosphate synthase-like protein [Geobacter sulfurreducens KN400]	gi|409913469	12.34000015	SLRMTGPPLKIVGIDR	16.34614	12.83388	12.53377	13.26809	0.785132147	0.766772461	0.811695605
796	gene 2 protein [Enterobacteria phage Sf6]	gi|41057280	15.94999981	EAIGEKMLEK	18.61211	14.93585	16.97483	16.4057	0.802480213	0.912031468	0.881452989
797	putative relaxase, mobilization protein [Pseudomonas savastanoi]	gi|410686350	11.05000019	EQKFYQVHGEHNDNVHTGQR	16.57498	14.28615	13.94093	14.54889	0.861910542	0.841082765	0.877762145
798	S-adenosylmethionine:tRNA ribosyltransferase-isomerase (Queuosine biosynthesis protein queA) [Thiomonas	gi|410692294	13.27000046	AAGEAGAALGGAVEVLVERVR	15.78932	14.73698	14.7604	15.72466	0.933351151	0.934834432	0.995904827
799	hypothetical protein Bd2131 [Bdellovibrio bacteriovorus HD100]	gi|42523598	11.64999962	SESLNYYVTAYYRFTK	16.55101	0	15.6978	14.59186	0	0.948449672	0.88162958
800	hypothetical protein Bd2370 [Bdellovibrio bacteriovorus HD100]	gi|42523816	13.18999958	TENMMTNVASVQMGWML	17.20846	16.44675	15.60631	15.11466	0.955736306	0.906897538	0.878327288
801	hypothetical protein [Vibrio mimicus]	gi|445940548	8.850000381	GLGEMNGDGAIVGRTVEFQVR	0	15.96599	16.17947	16.61756		1.013370922	1.040809871
802	hypothetical protein [Salmonella enterica]	gi|445980672	19.44000053	MSLREQVAMATSMTTLIELLK	16.66232	15.58831	15.4014	15.74709	0.93554259	0.924325064	0.945071875
803	flagellin [Escherichia coli]	gi|446001882	7.329999924	VTIGSK	15.2453	14.60271	13.59723	14.93142	0.95784996	0.891896519	0.97941136
804	hypothetical protein [Shigella dysenteriae]	gi|446051770	12.28999996	CLPELAK	16.47771	15.36317	15.02557	15.52598	0.932360747	0.911872463	0.942241367
805	fimbrial protein [Vibrio mimicus]	gi|446347371	12.94999981	YTFSIASAAAPAYTITATASTSK	18.12535	15.23745	15.98576	16.50843	0.840670663	0.881955935	0.910792343
806	hypothetical protein [Escherichia coli]	gi|446646152	9.800000191	SADNIDNLNKSAEYIK	17.07583	14.44079	15.64518	15.81578	0.845685978	0.916217835	0.926208565
807	hypothetical protein [Salmonella enterica]	gi|446859192	14.27000046	ETDMPLFYSLEHKDERS	17.61603	0	15.11079	15.8405	0	0.857786346	0.899209413
808	phage portal protein [Escherichia coli]	gi|446935673	2.589999914	TISPK	16.98027	15.20917	15.35681	15.68754	0.895696594	0.904391391	0.923868702
809	ADP-ribose pyrophosphatase [Vibrio sp. RC341]	gi|447072776	10.86999989	QDAYQLVLQGR	16.61102	15.67222	16.11344	15.89128	0.943483302	0.970045187	0.956670933
810	beta1,3-glucosyltransferase [Escherichia coli]	gi|447128666	8.920000076	YNLIPCSQGR	17.51505	15.23155	15.61253	15.80798	0.869626407	0.891377986	0.902536961
811	restriction endonuclease [Escherichia coli]	gi|447215788	20.65999985	FSITNVCGLITLK	16.93556	16.13125	15.68428	16.28452	0.952507623	0.926115227	0.961557811
812	predicted acetolactate synthase III large subunit [uncultured marine gamma proteobacterium EBAC20E09]	gi|45644652	13.84000015	KGIEEAFSIKDK	17.3411	16.76017	16.28747	15.75204	0.966499818	0.939240879	0.908364521
813	class I/II aminotransferase [Desulfovibrio vulgaris str. Hildenborough]	gi|46579304	12.07999992	FGVYLDPDTEAVVTMGAK	18.87341	17.03289	17.1452	17.78822	0.902480792	0.908431492	0.942501646
814	Helix-turn-helix-domain containing protein AraC type [Mannheimia haemolytica USDA-ARS-USMARC-183]	gi|472334061	9.170000076	KDSEM	15.91577	14.94188	14.75296	15.0645	0.938809747	0.926939758	0.946514055
815	TonB-dependent receptor [Escherichia coli]	gi|485655757	18.63999939	TTYKNAGKTRR	16.70702	15.95029	15.45422	16.00249	0.954705866	0.925013557	0.957830301
816	hypothetical protein [Escherichia coli]	gi|485663249	11.90999985	RRCRVLSGLQPLPNVGR	16.64436	15.46504	15.58883	16.04063	0.929145969	0.936583323	0.963727653
817	predicted protein [Escherichia coli]	gi|485666805	14.05000019	MPQKSHTTPTGIPGKNMLFIK	17.82059	14.97541	16.8771	16.40874	0.840343109	0.947056186	0.920774228
818	thiamine ABC transporter substrate-binding protein [Escherichia coli]	gi|485682957	13.72000027	TGLFAKSGVAADAVNVPGGWK	16.30305	16.31463	15.31949	15.84638	1.000710297	0.939670184	0.971988677
819	hypothetical protein [Escherichia coli]	gi|485707859	9.380000114	KSAIMS	17.40757	16.55378	16.09905	16.5178	0.950952947	0.924830404	0.948886031
820	type VI secretion protein [Escherichia coli]	gi|485721551	22.37000084	HIMILADAPASHPKSVHDK	0	16.87401	15.86989	16.15625		0.940493102	0.957463579
821	type IV conjugative transfer system coupling protein TraD [Escherichia coli]	gi|485863095	9.199999809	LSLKYQARPK	17.49809	15.69999	14.25651	16.02021	0.897240213	0.814746638	0.915540496
822	hypothetical protein [Salmonella enterica]	gi|486161996	16.13999939	STVMKMTGLTEDDLAQIR	17.97353	15.64615	16.04577	16.46947	0.87051069	0.892744497	0.916318052
823	type IV secretion protein Rhs [Salmonella enterica]	gi|486174332	0.899999976	AGTTS	16.20616	15.8324	15.10918	15.5665	0.976937165	0.932310924	0.960529823
824	hypothetical protein [Helicobacter pylori]	gi|487787012	19.46999931	RVVFTP	17.0594	14.85882	14.11736	14.85394	0.871004842	0.827541414	0.870718783
825	amidinotransferase [Vibrio cholerae]	gi|487815818	12.34000015	AVVMVPPK	16.98899	15.97519	0	16.25106	0.940326058	0	0.956564222
826	glyoxalase [Vibrio cholerae]	gi|487828854	9.520000458	MLGMKAVSFGAGR	15.8095	14.55585	14.95893	15.34839	0.920702742	0.946198805	0.97083336
827	hypothetical protein [Vibrio cholerae]	gi|487829084	12.25	MEAIDFSPIVSK	19.70432	16.99417	17.56954	14.69736	0.862459095	0.891659291	0.745895316
828	ATP-dependent DNA helicase DinG [Vibrio cholerae]	gi|487829697	12.06999969	NLDQKF	17.62175	15.88828	10.38743	15.46128	0.901628953	0.589466426	0.877397534
829	phosphoribosyltransferase [Neisseria meningitidis]	gi|488146813	7.929999828	NAGIKYDAMIAIGGGGFIPARMLR	17.33056	13.89789	15.38519	14.99704	0.801929655	0.887749155	0.865352303
830	thrombospondin [Stigmatella aurantiaca]	gi|488687343	10.89999962	SSVVVGK	16.87451	15.77013	15.02075	16.04443	0.934553359	0.890144366	0.950808646
831	hypothetical protein [Stigmatella aurantiaca]	gi|488688919	7.789999962	GRKNRLAPPADLFGQDFR	18.03542	16.41563	16.3967	16.92703	0.910188396	0.909138795	0.93854371
832	peptidase S9 [Stigmatella aurantiaca]	gi|488691710	14.75	SASYAR	18.46388	17.47939	17.37415	17.90297	0.946680221	0.940980444	0.969621228
833	phosphoribosylformylglycinamidine synthase [Simonsiella muelleri]	gi|488717522	25.35000038	SVVTALRGACALSPFR	17.96974	15.62359	16.33007	16.63249	0.869438846	0.908753827	0.92558323
834	hypothetical protein [Rhodobacter sphaeroides]	gi|488812193	15.72000027	DQSETAMADAAR	17.99837	15.59205	17.15592	17.00003	0.866303449	0.953192984	0.944531644
835	pyrrolo-quinoline quinone [Nitrosococcus oceani]	gi|488899592	11.56999969	DRYLRLPLSIIGDK	17.86728	16.75876	16.18428	15.65348	0.9379581	0.905805472	0.876097537
836	rhodanese [Campylobacter jejuni]	gi|488951680	11.35999966	DEGKKLAFICRSGHR	18.10878	0	14.91339	15.50309	0	0.823544711	0.856109026
837	phage Mu protein F like family protein, partial [Campylobacter jejuni]	gi|488953899	12.02000046	ELLTSKGWYGKKEIVNPK	17.28599	15.13218	16.31904	16.69563	0.875401409	0.944061636	0.965847487
838	epimerase [Pseudoalteromonas haloplanktis]	gi|489049581	17.82999992	SALESLV	17.59911	16.68406	16.29453	16.86913	0.948005893	0.925872388	0.958521766
839	sodium:proton antiporter [Pseudoalteromonas haloplanktis]	gi|489052614	14.97000027	VEFASAGKTRIEK	17.91804	16.27149	16.19466	17.213	0.908106579	0.903818721	0.960651946
840	RNA polymerase sigma factor [Comamonas testosteroni]	gi|489146020	9.489999771	VMLDS	17.96912	17.06353	0	17.25187	0.949602986	0	0.9600843
841	acetaldehyde dehydrogenase [Comamonas testosteroni]	gi|489161724	9.239999771	AARMGVATTHEGVEGLTR	15.78241	14.53583	14.4929	15.39853	0.921014598	0.918294481	0.975676719
842	phytase [Brevundimonas diminuta]	gi|489258404	10.98999977	PASLPLR	17.06021	14.99162	0	14.65248	0.878747682	0	0.858868677
843	molybdenum cofactor guanylyltransferase [Pseudomonas fluorescens]	gi|489264941	1.360000014	SGVSE	16.11672	15.31691	14.8583	15.11457	0.950373897	0.921918356	0.937819234
844	histidine kinase [Pseudomonas fluorescens]	gi|489268357	12.22999954	WAMRNGSVQR	15.93721	15.98721	13.80452	16.03104	1.003137312	0.866181722	1.00588748
845	multidrug MFS transporter [Ralstonia solanacearum]	gi|489357967	11.80000019	VTRGGK	17.27274	15.14641	15.08381	15.60942	0.876896775	0.873272567	0.903702597
846	hypothetical protein [Pseudomonas syringae]	gi|489462895	9.550000191	DDSLI	16.7671	14.21405	16.60352	16.26485	0.847734552	0.99024399	0.970045506
847	hypothetical protein, partial [Pseudomonas syringae]	gi|489471718	14.56000042	EPQPLP	18.57151	17.73023	17.01637	17.81618	0.954700506	0.916262059	0.959328563
848	precorrin-3B synthase [Methylosinus trichosporium]	gi|489706382	3.269999981	IGPPVGR	16.65646	14.96356	15.25881	15.71393	0.898363758	0.916089613	0.943413546
849	conjugal transfer protein TraG [Methylosinus trichosporium]	gi|489707700	17.98999977	STTEDAANGGLRR	18.71166	16.05455	17.20521	19.06658	0.857997099	0.919491376	1.018967852
850	carbamoyl phosphate synthase small subunit [Methylosinus trichosporium]	gi|489708154	14.02000046	FVEMMEKAK	0	17.6461	16.62004	16.74539		0.941853441	0.948956993
851	8-amino-7-oxononanoate synthase [Methylosinus trichosporium]	gi|489708190	2.180000067	KAMAGAGG	15.90392	14.62359	14.0539	14.57374	0.919495948	0.88367522	0.916361501
852	MULTISPECIES: hypothetical protein [Methylosinus]	gi|489708519	6.809999943	GIAVL	17.62772	16.67159	16.38902	16.94824	0.94575986	0.929729993	0.961453892

853	hypothetical protein [Gluconacetobacter hansenii]	gi|489712596	20.82999992	SKAKAL	19.32304	18.50675	18.19812	15.91694	0.957755612	0.941783487	0.823728564
854	hypothetical protein [Gluconacetobacter hansenii]	gi|489718016	11.14000034	VHNVLEAR	17.3993	16.0463	16.1887	16.85465	0.922238251	0.930422488	0.968697017
855	5-oxoprolinase (ATP-hydrolyzing), partial [Gluconacetobacter hansenii]	gi|489718059	21.13999939	LVIKEAVGDGAMRVQR	19.63751	18.21584	0	18.6512	0.927604365	0	0.949774182
856	urease accessory protein [Acinetobacter calcoaceticus]	gi|489749002	15.77000046	ILWHLHGLVPQAIEK	18.41256	0	14.70273	17.19636	0	0.798516339	0.933947262
857	integration host factor subunit alpha [Neisseria]	gi|489777041	14.27999973	GMVEHYYDK	18.12258	13.54232	14.95284	16.76441	0.747262255	0.82509444	0.925056477
858	glycoprotease [Neisseria flavescens]	gi|489778741	12.15999985	LSDYMVGKAAAITAPEGK	18.39626	0	15.13643	15.55319	0	0.822799308	0.845453913
859	tryptophanyl-tRNA synthetase [Neisseria flavescens]	gi|489779670	15.01000023	DTFMSK	16.9489	16.09227	15.70949	15.97375	0.949458077	0.92687372	0.942465293
860	adenylosuccinate synthase [Neisseria mucosa]	gi|489842743	16.55999947	IAPMITDVSRVLNEKNR	16.22678	15.03915	14.84101	15.36952	0.926810495	0.914599816	0.947170049
861	hypothetical protein, partial [Neisseria mucosa]	gi|489844766	10.93000031	GDTGATGAT	16.78416	16.03326	15.58498	15.98147	0.955261389	0.928552874	0.952175742
862	putative GTP pyrophosphokinase [Neisseria sicca]	gi|489853498	7.630000114	DTLSYK	17.09841	16.59212	15.97754	16.43075	0.970389644	0.934445951	0.960951925
863	type II secretion system protein F [Kingella denitrificans]	gi|489878917	19.53000069	MAERKPAR	17.30726	16.37419	16.1103	16.48828	0.946087942	0.930840584	0.952679974
864	4-hydroxythreonine-4-phosphate dehydrogenase [Kingella kingae]	gi|489881745	20.75	VCRLLCYVAER	17.46215	15.43231	16.27222	16.55682	0.883757727	0.931856616	0.948154723
865	hypothetical protein [Kingella kingae]	gi|489883126	9.979999542	MNPSRYRKK	17.37694	14.73734	14.99558	15.62988	0.848097536	0.862958611	0.89946101
866	hypothetical protein [Kingella oralis]	gi|489890198	9.659999847	MSRKWTQEK	16.07083	13.50294	14.7072	14.96972	0.840214227	0.915148751	0.931483937
867	terminase [Kingella oralis]	gi|489891955	8.279999733	EQLMLENAPAQFR	16.92683	15.09928	15.17813	15.87827	0.892032353	0.896690638	0.938053374
868	DNA repair protein RadA [Eikenella corrodens]	gi|489919849	17.95999908	QMGIAMIFVGHVTKDGAIAGPR	18.27371	15.15411	15.43056	15.61243	0.829284803	0.844413094	0.854365643
869	bifunctional proline dehydrogenase/pyrroline-5-carboxylate dehydrogenase [Eikenella corrodens]	gi|489921068	23.97999954	MLGKQFVTGQTIEEALK	18.51066	15.41506	14.80746	15.39557	0.832766633	0.799942304	0.831713726
870	hypothetical protein [Pantoea]	gi|489950016	14.31000042	GGLIVLLALLLAACSSSK	18.35239	14.1466	14.92781	15.05863	0.770831483	0.813398691	0.820526918
871	NLP/P60 family protein [Cardiobacterium hominis]	gi|490241820	12.11999989	TSEKKPTLAK	16.73528	16.34925	15.54834	15.7515	0.976933162	0.929075582	0.941215205
872	adhesin [Proteus mirabilis]	gi|490364780	12.86999989	DPHEDAQDIQYATTSIMGNK	0	16.34135	15.9371	16.39035		0.975262142	1.002998528
873	elongation factor 4 [Campylobacter upsaliensis]	gi|490399501	16.84000015	KTQKLESGEVGVVVLGLK	17.36133	15.73629	15.88348	16.45343	0.906398876	0.914876913	0.947705619
874	DNA methyltransferase [Campylobacter upsaliensis]	gi|490401568	10.25	MISLFSGCGGLDLGFIK	17.14342	15.02969	15.9782	15.63687	0.876703132	0.932031065	0.912120802
875	RecX family transcriptional regulator [Acinetobacter lwoffii]	gi|490405915	15.39000034	GFEMDAIIK	17.96206	14.94135	13.38253	13.93002	0.831828309	0.745044277	0.775524634
876	diaminopimelate epimerase [Campylobacter showae]	gi|490450262	10.60999966	IKGQNIAAGLK	16.63638	15.30878	15.1024	15.94488	0.920198986	0.907793643	0.958434467
877	hypothetical protein [Pseudoalteromonas haloplanktis]	gi|490465050	15.05000019	SMSGDK	15.70618	15.18364	14.75926	15.33477	0.966730293	0.939710356	0.97635262
878	phage integrase family protein [Comamonas testosteroni]	gi|490470422	16.90999985	SVGHNNKQTTK	17.64258	16.20715	16.61715	16.61692	0.918638317	0.941877549	0.941864512
879	peptidase M23 [Vibrio metschnikovii]	gi|490531810	0.899999976	AGSDSDS	16.09069	14.95659	14.90866	15.14334	0.929518249	0.926539508	0.94112434
880	hypothetical protein [Pseudomonas syringae]	gi|490543308	10.38000011	MVVASLMAGCSSTASDSSASAEAPVK	16.08775	13.86915	13.81233	0	0.862093829	0.858561949	0
881	hypothetical protein [Neisseria subflava]	gi|490655781	9.590000153	DGIMSAFIR	17.1701	15.11733	14.97492	15.54063	0.880445076	0.872151007	0.905098398
882	hypothetical protein [Neisseria subflava]	gi|490655870	10.07999992	AGSFNM	16.57503	15.56175	15.45604	15.86092	0.938867079	0.932489413	0.956916518
883	hypothetical protein [Burkholderia pseudomallei]	gi|490661632	16.60000038	TSEEMADLFALAGGQQWR	17.64953	16.68459	0	15.88388	0.945327723	0	0.899960509
884	hypothetical protein [Burkholderia pseudomallei]	gi|490677410	5.760000229	RPGRTAR	16.01718	14.19128	14.55193	15.87199	0.886003654	0.908520102	0.990935358
885	hypothetical protein [Burkholderia pseudomallei]	gi|490680047	16.53000069	ALRDPSGNPMN	18.19012	17.51433	16.99438	16.98609	0.962848513	0.934264315	0.933808573
886	hypothetical protein [Burkholderia pseudomallei]	gi|490681099	4.019999981	GANGGAANRRR	14.86693	12.63372	13.42435	13.26097	0.849786741	0.90296719	0.891977698
887	hypothetical protein [Rhizobium etli]	gi|490806738	3.869999886	AGPRSPP	16.00271	16.18789	15.02372	15.65386	1.01157179	0.938823487	0.978200567
888	filamentation induced by cAMP protein Fic [Rhizobium etli]	gi|490807239	14.92000008	MYGLERLEALPMSLR	16.90544	15.6063	16.06789	16.65237	0.923152547	0.950456776	0.985030262
889	sulfate transporter subunit [Yersinia frederiksenii]	gi|490848451	16.34000015	WGVGLSLLLLASGAIAK	17.60254	15.66097	16.27154	16.36668	0.889699441	0.924385912	0.929790814
890	hypothetical protein [Yersinia frederiksenii]	gi|490850441	27.78000069	HSHQKMEGK	17.67063	16.80193	16.40858	16.87947	0.950839331	0.92857923	0.955227403
891	N-acetyltransferase GCN5 [Yersinia rohdei]	gi|490851039	17.95999908	EEMRQMGITESVVADMIEK	17.15481	16.75904	15.71337	16.21194	0.976929503	0.915974587	0.945037573
892	voltage-gated potassium channel TrkA [Yersinia rohdei]	gi|490851973	3.200000048	GRTSDVERK	16.0491	15.41992	14.78694	15.25364	0.960796556	0.921356338	0.95043585
893	hypothetical protein [Yersinia ruckeri]	gi|490857051	7.909999847	APTTAK	0	17.20205	16.59261	17.07077		0.964571664	0.992368351
894	LysR family transcriptional regulator [Vibrio splendidus]	gi|490870632	21.52000046	DPMPLK	16.64972	16.23589	15.48795	15.84881	0.975144927	0.930222851	0.951896488
895	putative formate dehydrogenase oxidoreductase protein [Vibrio splendidus]	gi|490872708	11.80000019	SKVFIGLGGNFAQATPDTPR	15.70787	14.09337	14.22695	14.64614	0.897217127	0.905721145	0.932407768
896	flagellar cap protein FliD [Vibrio splendidus]	gi|490874191	11.35000038	LVDDQSALDRR	16.85361	15.51053	15.65092	15.9347	0.920309061	0.928639027	0.945476963
897	hemin storage protein [Yersinia mollaretii]	gi|491013926	12.93000031	GPIPFR	17.36945	15.61081	16.57914	16.85995	0.898750968	0.954499999	0.970666889
898	heme ABC transporter ATP-binding protein [Providencia stuartii]	gi|491056235	11.85999966	QERDRIYALPEMSEEDGYR	18.08186	17.00469	16.87554	17.29608	0.940428142	0.933285624	0.956543187
899	hypothetical protein [Providencia stuartii]	gi|491062915	10.36999989	NNNAMV	16.25142	15.3004	15.17335	15.55137	0.941480806	0.933663028	0.956923764
900	peroxidase [Serratia odorifera]	gi|491099991	10.64000034	LANPRTPASQSNLMLR	0	15.1951	15.29097	15.73736		1.006309271	1.035686504
901	formamidopyrimidine-DNA glycosylase [Acinetobacter johnsonii]	gi|491124672	12.73999977	ATARGKTK	17.20401	16.04372	16.04198	16.82125	0.932557003	0.932455863	0.977751699
902	ribosomal subunit interface protein [Rickettsia sibirica]	gi|491137290	3.180000067	YGSGK	17.52678	16.56683	16.3289	16.81999	0.945229529	0.931654303	0.959673711
903	aankyrin [Nitrococcus mobilis]	gi|491140863	18.5	EQALEGGGK	18.10932	16.78524	17.35746	17.29409	0.926884057	0.958482152	0.954982849
904	type VI secretion protein ImpA [Nitrococcus mobilis]	gi|491144499	16.43000031	AGKNVVIKGQK	0	17.60561	17.28245	17.79396		0.981644487	1.010698294
905	kinase [Acinetobacter radioresistens]	gi|491156396	16.5	IGETAVIAAKTGIK	18.3763	13.97856	14.5609	13.69004	0.760684142	0.792373873	0.744983484
906	hypothetical protein [Shigella dysenteriae]	gi|491170548	21.54999924	MIMILTVITIR	17.22206	15.29109	15.78754	16.20916	0.887878105	0.916704506	0.941185898
907	dTDP-glucose 4,6-dehydratase [Shigella flexneri]	gi|491226770	17.51000023	MVILNALEGKALPIYGK	16.43918	16.45113	15.44839	17.16963	1.000726922	0.939729962	1.044433481
908	invasion protein [Shigella flexneri]	gi|491233368	2.400000095	STGDA	16.61155	15.75102	15.22017	15.41892	0.948196887	0.916240206	0.928204773
909	DNA repair ATPase [Yersinia intermedia]	gi|491326240	27.04999924	NTESTHK	17.61828	15.78008	16.05433	16.82192	0.895665184	0.911231403	0.95479922
910	phophatidylinositol-4-phosphate 5-kinase [Helicobacter bilis]	gi|491359124	16.94000053	SEMPYK	17.24095	16.00996	15.93183	16.7165	0.928600802	0.924069149	0.969581143
911	hypothetical protein [Edwardsiella tarda]	gi|491427841	13.44999981	MGGCMKPSPFQLTRARR	18.13726	16.62824	16.82548	16.11535	0.916800002	0.927674853	0.88852175
912	tail protein [Edwardsiella tarda]	gi|491427876	18.40999985	QVTNKDTITR	18.32953	17.03337	17.39895	17.47478	0.929285694	0.949230559	0.953367599
913	hypothetical protein [Edwardsiella tarda]	gi|491430872	20.71999931	LIHFL	17.89437	17.13017	16.6556	17.02278	0.95729383	0.930773198	0.951292501

914	transcription accessory protein [Vibrio]	gi|491521689	10.10000038	DDSSSNSAMGGAFAAAFAKAK	15.52662	11.70794	14.69272	14.50484	0.754055937	0.946292239	0.93419173
915	predicted protein [Acinetobacter junii]	gi|491546401	14.77999973	LYDDYECGLLEK	16.00951	14.74155	12.69003	14.67437	0.920799575	0.79265574	0.916603319
916	NH(3)-dependent NAD(+) synthetase [Sutterella wadsworthensis]	gi|491572329	12.11999989	SDILGISMPGFGTSSGTRASAEALMR	15.25228	15.1992	0	14.67629	0.996519865	0	0.96223581
917	hypothetical protein [Sutterella wadsworthensis]	gi|491574849	18.64999962	KSVEIYGER	17.14861	15.95547	16.13204	16.82153	0.930423515	0.940719977	0.980926734
918	hypothetical protein [Sutterella wadsworthensis]	gi|491574963	17.46999931	LPVVTLPDVLRHLAGPAAEK	17.66263	15.60384	15.79898	16.27037	0.883438084	0.894486268	0.921174819
919	DNA helicase [Sutterella wadsworthensis]	gi|491575064	11.07999992	MSSDILANLNPEQHR	16.25731	13.43359	12.99063	14.36656	0.826310749	0.799063929	0.883698472
920	tetrathionate reductase subunit A [Vibrio harveyi]	gi|491581361	24.30999947	DAYLSLAGENGLEGRSTACAR	17.38371	14.55032	17.07833	17.46009	0.837008901	0.982432979	1.004393769
921	phospholipase [Vibrio harveyi]	gi|491584695	17.30999947	WISEAVAAGKNCLAQAKDIK	17.57255	14.54367	0	14.88926	0.827635716	0	0.847302184
922	sterol desaturase [Shewanella benthica]	gi|491642399	16.55999947	RWSMASLNPLTASIR	15.87571	16.57394	15.05314	15.86726	1.043981025	0.948186884	0.99946774
923	MSHA biogenesis protein MshM [Grimontia hollisae]	gi|491644726	15.14999962	LLGNLETEQRK	16.49952	15.39208	15.09137	15.56314	0.932880472	0.914655093	0.943248046
924	cobalt-zinc-cadmium resistance protein [Grimontia hollisae]	gi|491647392	7.900000095	GEGIFA	18.29802	13.4441	16.28385	17.63478	0.734729769	0.889924156	0.963753455
925	flagellar hook protein [Grimontia hollisae]	gi|491649407	10.51000023	TSLDTMKDLMYDFR	0	16.69481	16.3763	16.21768		0.980921616	0.971420459
926	sialic acid-binding protein [Vibrio mimicus]	gi|491652032	8.409999847	EFDNKMGQPIVSKLAAM	15.29257	12.51379	11.99259	12.74746	0.818292151	0.784210241	0.83357212
927	phage tail protein [Actinobacillus pleuropneumoniae]	gi|491790724	22.12999916	GGARIAPNLLPVTAGTLAQGK	17.2727	15.6551	17.17902	16.94689	0.906349326	0.994576412	0.981137286
928	GntR family transcriptional regulator [Ruegeria]	gi|491823456	17.54000092	APMSQTKNSTQK	16.04556	16.33541	16.19555	13.40781	1.018064187	1.009347757	0.835608729
929	50S rRNA methyltransferase [Actinobacillus ureae]	gi|491829259	16.79999924	VIVVESLYR	17.16775	15.98914	16.01637	16.18683	0.931347439	0.932933553	0.942862635
930	hypothetical protein [Actinobacillus ureae]	gi|491836246	14.60000038	LLNMVS	16.24013	15.06581	14.92853	15.29633	0.927690234	0.9192371	0.941884702
931	predicted bacteriophage tail assembly protein [Haemophilus influenzae]	gi|491883564	10.71000004	VPVGGGNSVK	15.63173	14.9919	14.60172	15.10628	0.95906851	0.934107741	0.966385678
932	malate transporter [Lautropia mirabilis]	gi|491921771	8.619999886	STGLM	16.12622	15.28732	14.95419	15.16501	0.94797913	0.927321468	0.940394587
933	ImcF-like family protein, partial [Pseudomonas amygdali]	gi|492043297	16.26000023	ANFVRELMAR	17.66335	15.60952	15.49904	0	0.883723642	0.877468883	0
934	hypothetical protein [Pseudomonas amygdali]	gi|492053223	14.34000015	SLLQTATPPR	17.9281	13.38861	16.54682	17.22201	0.746794697	0.922954468	0.960615458
935	oxidoreductase [Pseudomonas amygdali]	gi|492056221	2.190000057	DMLGIEG	17.01588	16.08741	15.72422	16.25203	0.945435088	0.924090908	0.95510958
936	methionine aminopeptidase, type I [Pseudomonas amygdali]	gi|492071390	17.06999969	EGMVFTIEPMINAGKAGTR	17.29473	0	14.48	15.18294	0	0.837249266	0.877894017
937	hypothetical protein [Pseudomonas amygdali]	gi|492083506	17.54999924	ASRLLSLSFSISK	17.84871	15.42022	16.53692	16.4604	0.863940307	0.926505053	0.922217908
938	LuxR family transcriptional regulator [Pseudomonas amygdali]	gi|492091592	21.70999908	RSGGSDTK	19.24832	18.00808	17.9999	18.27931	0.935566325	0.935141353	0.949657425
939	NAD-dependent dehydratase [Acidovorax delafieldii]	gi|492261047	9.739999771	TDFGSR	16.37256	15.88533	15.06126	15.30391	0.970241062	0.919908676	0.934729205
940	erythromycin biosynthesis sensory transduction protein eryC1 [Acidovorax delafieldii]	gi|492263029	12.60000038	NAVARVVDSGWVVLGPEVR	17.82193	16.49728	16.64462	16.74809	0.925673033	0.933940376	0.939746144
941	hypothetical protein [Acidovorax delafieldii]	gi|492263521	21.44000053	IKHVPYR	16.51246	15.7293	15.22791	15.80599	0.952571573	0.92220723	0.957215945
942	diguanylate cyclase [Acidovorax delafieldii]	gi|492281117	16.17000008	ESGASAHR	17.26019	12.33333	0	17.31241	0.714553548	0	1.003025459
943	hypothetical protein [Actinobacillus minor]	gi|492360919	11.81000042	AVGSAKGCDMMSLLIKK	17.41709	16.00236	16.65338	16.7602	0.918773458	0.956151688	0.962284744
944	sulfatase [Actinobacillus minor]	gi|492362060	8.06000042	MENMPFVQTGKDAKMK	15.2162	12.76299	12.68822	0	0.838776436	0.833862594	0
945	iron ABC transporter substrate-binding protein [Actinobacillus minor]	gi|492369946	13.98999977	NLTASKK	16.76851	15.75695	15.41002	15.99039	0.939675022	0.918985646	0.953596354
946	glucosyltransferase [Sulfitobacter sp. EE-36]	gi|492459387	12.93999958	DNLRSGAAPEGSSHDTIVQPA	18.26633	17.30989	17.06058	17.5534	0.947639181	0.933990572	0.960970266
947	hypothetical protein [Sulfitobacter sp. EE-36]	gi|492459612	10.36999989	PDHAPSK	16.94015	16.20391	15.71506	16.17629	0.956538767	0.927681278	0.95490831
948	translocation protein TolB precursor [Sagittula stellata]	gi|492469179	17.26000023	YGTPVWSPRGDRIAFTKQNK	19.80691	15.56926	17.85901	0	0.786051938	0.901655533	0
949	segregation protein B [Sagittula stellata]	gi|492488380	15.39000034	VGQISIV	17.27935	16.07224	16.28745	16.70389	0.930141469	0.94259622	0.966696664
950	hypothetical protein [Campylobacter gracilis]	gi|492515890	6.730000019	RGVAGT	17.46768	15.7357	15.04102	15.45984	0.900846592	0.861077144	0.885053997
951	ATPase [Oxalobacter formigenes]	gi|492534489	14.36999989	HTDKNPQGRGKDSNVASASDK	18.68703	16.96849	16.93135	17.8767	0.90803568	0.906048206	0.956636769
952	universal stress protein [Oxalobacter formigenes]	gi|492534832	11.14999962	SDIRLKAGAEMQAFIAALK	16.25366	15.63355	14.00137	15.12506	0.961847978	0.861428749	0.930563332
953	hypothetical protein [Oxalobacter formigenes]	gi|492537729	17.05999947	DLQSTLDVVVTHAVTDELHR	0	16.27214	15.96597	13.73587		0.981184405	0.844134207
954	uroporphyrin-III C-methyltransferase [Oxalobacter formigenes]	gi|492542645	12.38000011	LQSGDDVSMEAKTIAK	17.63562	16.98015	0	16.85578	0.962832608	0	0.955780404
955	general secretion pathway protein G [Pseudomonas coronafaciens]	gi|492582213	7.71999979	QVGGNVDKGKYGAGKAQLASLGMK	17.07498	14.02316	14.03309	14.36275	0.821269483	0.821851036	0.841157647
956	hypothetical protein [Ruegeria lacuscaerulensis]	gi|492825050	9.800000191	SVLEGGIGIFPR	17.20439	15.22828	15.33971	15.74546	0.8851392	0.891616035	0.915200132
957	transposase for insertion sequence element A [Ruegeria lacuscaerulensis]	gi|492825685	5.070000172	KCGSIEADMLMR	15.78735	15.46199	14.39856	0	0.979391095	0.912031468	0
958	dioxygenase [Ruegeria lacuscaerulensis]	gi|492825937	10.34000015	MAFFGKDFERNEVSAVTR	16.73931	15.66884	15.89843	16.24995	0.93605053	0.949766149	0.97076582
959	hypothetical protein [Xanthomonas vesicatoria]	gi|492838104	16.12999916	HSSGNR	19.82188	18.50273	18.43715	18.81903	0.933449804	0.930141339	0.949406918
960	Myo-inositol-monophosphatase [Desulfovibrio fructosivorans]	gi|492838397	21.23999977	AAVAAAGER	18.22103	17.0916	17.12375	17.44372	0.93801503	0.939779475	0.957339953
961	tRNA pseudouridine synthase D [Moritella sp. PE36]	gi|492903359	8.489999771	SFYISAARSLIFNQVVSER	17.49926	16.33413	16.56672	16.95612	0.933418327	0.946709747	0.968962116
962	phosphomannomutase [Rickettsiella grylli]	gi|492904583	15.23999977	SLMWKTGHSIMK	16.59954	15.0268	15.62119	15.50519	0.905254001	0.94106162	0.934073474
963	glycosyl transferase family 1 [Burkholderia graminis]	gi|492928686	16.40999985	QGGSLPK	17.57277	17.20191	16.41086	16.96529	0.978895757	0.933880088	0.965430607
964	hypothetical protein [Acetobacter pomorum]	gi|493059835	8.779999733	QAAEFLCMSR	17.43239	15.55729	15.82059	16.54619	0.892435862	0.90753993	0.949163597
965	phosphotransferase [Enterobacter cancerogenus]	gi|493177927	31.09000015	EVARALIAMHVK	17.97309	0	13.66119	16.17625	0	0.760091337	0.900026095
966	hypothetical protein [Achromobacter piechaudii]	gi|493250031	10.32999992	TGHADDIAR	15.78469	14.91187	14.50451	15.00339	0.944704647	0.918897362	0.950502671
967	glycogen synthase [Achromobacter piechaudii]	gi|493251594	12.90999985	GSMPMPI	16.624	14.29254	13.85661	14.75433	0.859753369	0.833530438	0.887531882
968	hypothetical protein [Achromobacter piechaudii]	gi|493255102	17.85000038	GIRTVAELLARAQAEPGK	18.05677	15.79583	17.03631	17.63021	0.87478713	0.943486017	0.976376727
969	hypothetical protein [Photobacterium profundum]	gi|493273656	11.14999962	FNASYQLIKEK	16.88907	15.3974	16.21841	16.83445	0.911678381	0.960290294	0.996765956
970	5-methyltetrahydropteroyltriglutamate--homocysteine methyltransferase [Photobacterium profundum]	gi|493273670	14.09000015	IARGKAPTGCSCAASDMTK	18.07043	15.68905	16.38034	16.64549	0.86821675	0.906472065	0.921145208
971	hypothetical protein [Mannheimia haemolytica]	gi|493294557	15.10999966	QMLKLYPTTENVQPPFYK	17.2919	16.59493	16.7945	15.80656	0.959693845	0.971235087	0.914101978
972	penicillin-binding protein 2 [Hylemonella gracilis]	gi|493340836	11.26000023	LQHLVEQMFGDRR	17.47554	14.92939	15.0397	15.95137	0.85430207	0.860614322	0.912782667
973	hypothetical protein [Agrobacterium sp. ATCC 31749]	gi|493354828	15.47000027	QIEPGADVGRR	16.76051	15.52354	15.56389	15.86203	0.926197353	0.928604798	0.946393039
974	hypothetical protein [Achromobacter xylosoxidans]	gi|493429754	10.28999996	QGWQLMHGNSGSVFEPR	16.50101	14.7427	15.20048	15.93226	0.89344228	0.921184824	0.965532413

975	phytanoyl-CoA dioxygenase [Burkholderia multivorans]	gi|493447778	11.93000031	TRMCETLYPVLRERR	15.25326	0	13.9517	13.52079	0	0.914670044	0.88641969
976	50S ribosomal protein L6 [delta proteobacterium NaphS2]	gi|493464246	7.860000134	KAGKAGAAK	16.41427	14.92624	15.07352	15.25896	0.909345344	0.918318024	0.929615511
977	conserved hypothetical protein [delta proteobacterium NaphS2]	gi|493466275	11.82999992	LVTFTMER	17.40936	0	15.20939	15.82871	0	0.873632919	0.909206886
978	conserved hypothetical protein [delta proteobacterium NaphS2]	gi|493467440	7.449999809	RETLVNHK	17.6152	17.21957	15.94591	16.33191	0.97754042	0.905235819	0.927148712
979	glycogen/starch synthase, ADP-glucose type [delta proteobacterium NaphS2]	gi|493468036	16.62000084	GMAMAAVLR	15.88199	15.31708	14.87009	15.30373	0.96443078	0.936286322	0.963590205
980	AMP-binding enzyme [delta proteobacterium NaphS2]	gi|493468391	3.450000048	GPMVFK	17.32689	14.07781	14.38547	14.19309	0.812483371	0.830239587	0.819136614
981	DNA repair protein [delta proteobacterium NaphS2]	gi|493469405	4.860000134	QMMGGR	16.68525	15.47234	15.50178	15.68601	0.927306453	0.929070886	0.940112375
982	hypothetical protein [Xanthomonas gardneri]	gi|493495256	9.930000305	DLDTRLMGLGMQR	17.07372	15.1962	15.42829	0	0.890034509	0.903627915	0
983	tRNA uridine 5-carboxymethylaminomethyl modification protein [Xanthomonas gardneri]	gi|493497392	19.26000023	IAGMTPAAISLLLVHLER	17.16083	15.35621	15.65169	15.78037	0.894840751	0.912059032	0.919557504
984	lysine transporter LysE [Ochrobactrum intermedium]	gi|493511513	7.559999943	EVIRSAILVNLLNPK	17.6624	13.79472	13.06536	13.64519	0.781021832	0.73972733	0.772555825
985	5-oxoprolinase [Ochrobactrum intermedium]	gi|493514780	8.989999771	GGTFTDVIGRDPQGQLHAR	17.90551	15.10062	14.82334	15.25219	0.843350455	0.827864719	0.851815447
986	AraC family transcriptional regulator [Burkholderia cenocepacia]	gi|493526637	4.960000038	RTAHR	15.47503	15.46815	14.22605	14.67042	0.999555413	0.919290625	0.948005917
987	hypothetical protein [Proteus penneri]	gi|493581887	14.09000015	ALIMRPYGR	16.16578	15.27705	15.07858	15.49113	0.945023995	0.932746827	0.958266783
988	hydrogenase 2 b cytochrome subunit [Proteus penneri]	gi|493582141	14.72000027	MLFVCALFILIGAAMWR	16.50677	15.22477	15.43792	15.66772	0.922334897	0.93524778	0.949169341
989	beta-N-acetylhexosaminidase [Photobacterium sp. SKA34]	gi|493695732	20.53000069	GITVDDK	15.38422	14.66645	14.23882	14.4514	0.953343751	0.925547087	0.939365142
990	multidrug transporter AcrB [Photobacterium sp. SKA34]	gi|493696524	13.11999989	LYRTANDFK	16.18147	14.23886	14.735	15.46694	0.879948484	0.910609481	0.955842702
991	hypothetical protein [Helicobacter canadensis]	gi|493705562	15.40999985	MVAATPK	18.25093	16.99565	17.10006	17.42373	0.931221039	0.936941844	0.954676282
992	LacI family transcription regulator [Providencia alcalifaciens]	gi|493709768	13.09000015	DPQLELEAAK	16.47078	14.85925	14.90806	15.83546	0.902158246	0.905121676	0.961427449
993	nuclease [Providencia alcalifaciens]	gi|493710680	26.35000038	DLTGVL	19.69964	18.60792	17.84584	18.87098	0.944581728	0.905896758	0.957935272
994	polymerase [Citrobacter youngae]	gi|493736859	10.72999954	RAEDKPRPLFTINR	0	15.24093	14.70336	14.13145		0.96472853	0.927203917
995	hypothetical protein [Candidatus Regiella insecticola]	gi|493755517	16.31999969	SALAQLPDAIK	0	17.69391	19.08815	19.22512		1.078797733	1.086538815
996	hypothetical protein [Candidatus Regiella insecticola]	gi|493755622	15.14000034	LQAVEQSGAGMSLSLIEPGK	17.93918	15.2203	15.78347	16.56821	0.848439003	0.8798323	0.923576774
997	membrane protein insertase [Candidatus Regiella insecticola]	gi|493756195	12.40999985	LSNGLAAIGFKSMPVVIPPGEQK	17.51373	15.30649	0	15.55243	0.873970879	0	0.888013576
998	hypothetical protein [Burkholderia ambifaria]	gi|493802068	18.61000061	CATDQGYGEAANALGINLK	16.71836	16.83738	15.18923	16.21393	1.007119119	0.908535885	0.969827782
999	conjugal transfer protein TrbC [Burkholderia ambifaria]	gi|493809538	27.20999908	HIQTLFSQIKKK	0	16.29799	16.07689	15.23215		0.98643391	0.934602979
1000	diguanylate cyclase [Burkholderia ambifaria]	gi|493810045	3.359999895	SIAYA	16.84603	15.50332	15.6389	16.03075	0.920295167	0.928343354	0.951604028
1001	Alkylated DNA repair protein [Burkholderia dolosa]	gi|493818585	18.57999992	RTAPAGGGISMR	17.72098	14.11231	16.50078	17.23702	0.796361714	0.931143763	0.972689998
1002	Signal transduction histidine kinase [Burkholderia dolosa]	gi|493818602	16.22999954	FTQHGTISVSTTLLDR	16.67023	0	14.53928	15.91984	0	0.87217033	0.954986224
1003	dimethylallyltransferase [Alteromonadales]	gi|493844368	6.460000038	DADGNALVAGKK	17.03318	15.96929	15.55059	13.40596	0.937540142	0.912958708	0.787049746
1004	histidine kinase [Alteromonadales bacterium TW-7]	gi|493847325	9.729999542	LVGHVYMRGSLESLDDYIK	16.43461	15.19873	15.10978	15.46904	0.924800163	0.919387804	0.941247769
1005	conserved hypothetical protein [Helicobacter winghamensis]	gi|493855127	25.42000008	IDAMSVKEAREK	16.04784	16.148	15.28452	15.50255	1.006241338	0.95243472	0.966020972
1006	conserved hypothetical protein [Helicobacter winghamensis]	gi|493855229	17.70000076	AGEAMELERVFGLK	17.16116	15.51022	15.74367	16.3686	0.903797878	0.917401271	0.953816642
1007	dimethyl sulfoxide reductase subunit A [Enterobacter hormaechei]	gi|493862104	14.77999973	RGIKNGDMVRVFNDR	17.34827	14.67309	14.76646	15.48356	0.845795575	0.851177668	0.892513202
1008	RNA helicase [Vibrio brasiliensis]	gi|493933784	16.62999916	KPKTGHQDGQRSGDNAR	15.75865	13.79055	14.67227	15.8812	0.87510986	0.931061354	1.007776681
1009	glutamine amidotransferase [Vibrio brasiliensis]	gi|493934652	30.20999908	RDELSLSLIR	18.16657	15.42591	15.90924	16.32323	0.849137179	0.875742642	0.898531203
1010	hypothetical protein [Labrenzia aggregata]	gi|493993341	8.600000381	GDSAMNR	17.10273	16.21251	15.84566	16.3951	0.947948661	0.926498869	0.958624734
1011	hypothetical protein [Idiomarina baltica]	gi|494014010	6.789999962	GRLFGGS	16.60491	15.60612	15.38863	15.26157	0.939849719	0.926751786	0.919099833
1012	preprotein translocase subunit SecA [Idiomarina baltica]	gi|494014389	13.63000011	KIPHAVLNAK	0	15.84617	16.31715	16.57505		1.029722009	1.045997235
1013	general secretion pathway protein D [Idiomarina baltica]	gi|494014555	6.170000076	GVALMPDK	15.89115	15.09643	14.73852	14.94289	0.949989774	0.927467175	0.940327793
1014	type III secretion protein [Vibrio coralliilyticus]	gi|494016039	23.70000076	GDVKGTKK	17.38445	14.9954	16.25206	17.92983	0.862575463	0.934861902	1.031371714
1015	hypothetical protein [Vibrio coralliilyticus]	gi|494016308	12.18000031	ESEIHVIGVGKAMTPSSFK	15.17849	13.49582	16.5365	14.61714	0.889141146	1.089469374	0.963016743
1016	hypothetical protein [Plesiocystis pacifica]	gi|494027752	7.75	GQVHIDPWEVTTGDK	16.10065	14.92696	13.77757	15.35429	0.927102943	0.855715142	0.953644108
1017	prolyl-tRNA synthetase [Plesiocystis pacifica]	gi|494028558	18.89999962	SGKGGNK	17.80346	15.14597	14.78897	14.87578	0.850731824	0.830679542	0.835555561
1018	hypothetical protein [Plesiocystis pacifica]	gi|494028765	14.02000046	LGMIDGRTGR	16.74701	15.9684	15.55112	15.60474	0.953507522	0.928590835	0.931792601
1019	hypothetical protein [Plesiocystis pacifica]	gi|494029916	21.01000023	QARFGH	17.02125	15.93454	15.6565	15.66288	0.936155688	0.919820812	0.920195638
1020	putative adventurous gliding protein T [Plesiocystis pacifica]	gi|494034354	9.539999962	DIEVYIAMGVAKR	17.82256	15.79264	16.09009	17.11556	0.886103904	0.902793426	0.960331176
1021	glycosyl hydrolase [Candidatus Pelagibacter ubique]	gi|494054849	25.31999969	EMNDVALYSPVISKFIKKK	17.2525	18.01399	15.96711	17.13208	1.044137951	0.925495435	0.993020142
1022	glycosyl transferase [Candidatus Pelagibacter ubique]	gi|494055161	22.87999916	SLRFGH	17.1291	16.17579	15.80018	16.32582	0.944345587	0.922417407	0.953104366
1023	phosphoribosylaminoimidazole carboxylase [Roseomonas cervicalis]	gi|494060867	31.84000015	HHHHILDLSFAPAR	18.32989	15.14281	14.682	15.07412	0.826126616	0.800986804	0.822379185
1024	histidinol-phosphate aminotransferase [Roseomonas cervicalis]	gi|494061246	12.93000031	EAHRYPDGNAKELREAIGAR	16.65793	16.03374	15.63119	16.38656	0.962528958	0.93836329	0.98370926
1025	AMP-dependent synthetase [Roseomonas cervicalis]	gi|494062916	12.11999989	LLAARLRDEEEEQAAR	0	16.03776	15.99472	15.31884		0.997316333	0.955173291
1026	isocitrate lyase [Roseomonas cervicalis]	gi|494062961	9.369999886	HTLAEMGARR	18.38897	17.36868	17.24763	17.66531	0.944516196	0.937933446	0.960647062
1027	hypothetical protein, partial [Roseomonas cervicalis]	gi|494064227	11.67000008	TGRGMPR	16.50685	15.16687	15.29269	15.46768	0.918822792	0.926445082	0.937046135
1028	urease accessory protein UreG [Bermanella marisrubri]	gi|494074615	7	DTRKMR	0	14.74731	14.50496	13.96101		0.983566494	0.946681802
1029	23S rRNA pseudouridine synthase [Bermanella marisrubri]	gi|494075511	0.360000014	GGKPGAK	16.70897	15.63444	0	16.04772	0.935691428	0	0.960425448
1030	probable transcriptional regulator, GntR family protein [Neptuniibacter caesariensis]	gi|494078713	19.02000046	LGYMVLPPK	17.32361	16.53254	16.15231	16.27627	0.95433573	0.932387072	0.939542624
1031	exoribonuclease R [Neptuniibacter caesariensis]	gi|494079081	10.75	LKAMCRDGQLMSNRR	16.8899	14.99614	15.02171	15.39559	0.887876186	0.889390109	0.911526415
1032	Clp protease [Fulvimarina pelagi]	gi|494125712	11.93999958	VSIEGMITEDR	16.67535	15.36084	15.4894	15.91649	0.92117047	0.928880053	0.95449211
1033	malonyl CoA-ACP transacylase [Methylophaga aminisulfidivorans]	gi|494244789	14	MPTTPVIHNASVTSAVDVDAIK	0	15.56409	15.06078	14.92929		0.967662099	0.959213806
1034	ATP-dependent metalloprotease [Methylophaga aminisulfidivorans]	gi|494247672	12.98999977	GILMAGSPGTGK	17.62477	15.52166	15.44157	15.80383	0.880673053	0.87612888	0.896682907
1035	ATP-dependent DNA helicase RecQ [Marinobacter algicola]	gi|494260416	9.460000038	ELDGA	17.1522	15.41589	15.16711	15.73564	0.89877042	0.884266158	0.917412344

1036	allophanate hydrolase [Marinobacter algicola]	gi|494263655	11.67000008	LETFKTSLAGP	16.26499	14.78132	15.50311	15.96434	0.908781376	0.953158287	0.981515513
1037	gluconate kinase [Marinobacter algicola]	gi|494264297	8.93999958	MGVSGCGK	16.97658	16.20269	15.92488	15.72964	0.954414258	0.938049949	0.926549399
1038	glutamate synthase [Marinobacter algicola]	gi|494265716	16.79000092	GTGFTPVK	19.59113	18.51932	18.42244	18.98229	0.945291058	0.940345963	0.968922671
1039	transcriptional regulator, ArsR family protein [Erythrobacter sp. NAP1]	gi|494285712	23.19000053	IGQPALSQQLGVLR	18.67651	16.07478	16.09365	16.15872	0.860695066	0.861705426	0.865189481
1040	hypothetical protein [Erythrobacter sp. NAP1]	gi|494285782	15.42000008	MTEKTSHSDKGGGGSYWR	17.52842	14.90427	14.93001	15.73894	0.850291698	0.85176017	0.897909794
1041	hypothetical protein [Erythrobacter sp. NAP1]	gi|494289743	21.38999939	GGRRGGGGMAMLRR	0	16.36876	15.41484	15.97238		0.941723136	0.97578436
1042	PII uridylyl-transferase [Erythrobacter sp. NAP1]	gi|494289777	16.96999931	TVADTMRMAKEDLTIR	16.06247	13.94331	15.9725	15.96022	0.868067614	0.994398744	0.993634229
1043	hypothetical protein [Erythrobacter sp. NAP1]	gi|494289900	12.98999977	WTALVEAKVR	18.39126	16.62928	12.65969	16.78889	0.90419471	0.688353598	0.912873289
1044	C4-dicarboxylate ABC transporter permease [Hoeflea phototrophica]	gi|494371160	24.38999939	DDELPK	16.70105	10.68654	15.6201	15.97624	0.639872343	0.935276525	0.956600932
1045	Mn2+-dependent serine/threonine protein kinase [Hoeflea phototrophica]	gi|494373641	13.97000027	AGSQQVKS	16.55139	16.00306	15.42846	15.97001	0.96687106	0.932154943	0.964874249
1046	N-methylproline demethylase [Loktanella vestfoldensis]	gi|494385072	10.44999981	ALQDKDVTFTVTRR	18.4551	16.66536	14.77655	18.16721	0.903021929	0.800675694	0.984400518
1047	cobaltochelatase [Loktanella vestfoldensis]	gi|494387405	18.75	MAKKGPK	15.63361	14.75024	14.51781	15.23006	0.943495456	0.928628129	0.974187024
1048	flagellar scaffolding protein FlgD [Loktanella vestfoldensis]	gi|494388140	11.94999981	MSETMSGIADQMGQFR	0	16.06901	17.18381	17.39278		1.069375774	1.082380308
1049	ATPase AAA [Loktanella vestfoldensis]	gi|494389724	7.170000076	VTAAA	16.39515	15.61592	15.08283	15.65527	0.95247192	0.919956816	0.95487202
1050	putative toxin secretion transmembrane protein [marine gamma proteobacterium HTCC2143]	gi|494427221	11.31999969	SIFVKNGVR	17.30171	16.60144	0	16.34454	0.959525966	0	0.944677723
1051	short-chain dehydrogenase [marine gamma proteobacterium HTCC2143]	gi|494429549	8.479999542	GQAIANR	0	15.72573	16.62731	17.18633		1.05733152	1.092879631
1052	hypothetical protein [marine gamma proteobacterium HTCC2143]	gi|494432820	21	TDQEPPK	17.63876	16.41241	16.62734	16.96469	0.930474138	0.942659235	0.961784729
1053	16S rRNA methyltransferase [marine gamma proteobacterium HTCC2143]	gi|494433724	12.36999989	VAEKIA	18.05053	17.38163	16.74861	17.22767	0.962942916	0.927873586	0.954413527
1054	hypothetical protein [marine gamma proteobacterium HTCC2148]	gi|494435235	15.05000019	NLRNMKVARDALAPGYYMR	18.44091	15.275	14.83603	15.15301	0.828321379	0.804517239	0.821706196
1055	damage-inducible protein CinA [marine gamma proteobacterium HTCC2080]	gi|494440110	5.610000134	LAEKLQAVLRNQNK	15.36878	15.44001	14.28647	14.95065	1.004634721	0.929577364	0.972793546
1056	DEAD/DEAH box helicase [Pseudomonas syringae group genomosp. 3]	gi|494457088	18.35000038	EQLIGLIQADAKFMNER	16.6311	16.12873	15.33741	15.95594	0.969793339	0.922212602	0.959403768
1057	hypothetical protein [Pseudomonas syringae group genomosp. 3]	gi|494459490	9.920000076	APAMMMAK	16.85171	16.11902	15.39366	16.17927	0.956521326	0.913477623	0.960096631
1058	beta-hexosaminidase [Oceanicola granulosus]	gi|494464427	15.02999973	ADAALAQRK	16.2033	15.67724	15.0712	15.55684	0.967533774	0.930131516	0.960103189
1059	branched-chain amino acid ABC transporter ATPase [Oceanicola granulosus]	gi|494464688	12.06999969	VSKGMGFVPQVR	16.24118	15.43873	14.99681	15.46018	0.950591644	0.923381799	0.951912361
1060	general secretion pathway protein [Oceanicola granulosus]	gi|494465510	9.270000458	GAPSRALQVPMNR	17.20427	15.50496	15.64135	16.22647	0.901227428	0.909155111	0.943165272
1061	isoleucyl-tRNA synthase [Oceanicola granulosus]	gi|494466945	13.86999989	AGGARGK	16.63847	11.22426	15.32685	15.53744	0.674596883	0.921169434	0.933826247
1062	pH regulation protein F [delta proteobacterium MLMS-1]	gi|494503462	10.86999989	SGGTP	0	16.05169	15.77928	15.55185		0.983029201	0.9688606
1063	hypothetical protein [delta proteobacterium MLMS-1]	gi|494504707	13.5	GAPTMPTK	16.42132	15.65517	15.23344	15.32402	0.953344189	0.927662332	0.933178332
1064	hypothetical protein [Neisseria bacilliformis]	gi|494579732	15.85999966	KPSNGR	19.28514	18.31213	18.07825	18.49483	0.949546127	0.937418655	0.959019743
1065	hypothetical protein [Neisseria bacilliformis]	gi|494580113	20.77000046	FTTPPR	16.60251	16.0773	15.49281	15.9949	0.968365627	0.933160709	0.963402522
1066	FAD-binding protein [Marinobacter sp. ELB17]	gi|494590783	8.590000153	DHDLDGHFIR	17.60421	15.22242	15.38242	15.65931	0.864703386	0.873792121	0.889520745
1067	23S rRNA pseudouridylate synthase C [Marinobacter sp. ELB17]	gi|494593189	18.62999916	YEMPNGERRVK	18.19233	16.97741	17.29864	14.47758	0.93321801	0.950875451	0.795806804
1068	ribonuclease III [Gluconacetobacter sp. SXCC-1]	gi|494641216	24.30999947	TGQGVAGSK	17.68201	16.675	16.3566	16.85607	0.943048895	0.925041893	0.953289247
1069	cell division protein FtsA [Gluconacetobacter sp. SXCC-1]	gi|494642229	9.210000038	DRLEMAAVGPASDGR	16.32574	16.32064	15.26301	15.89063	0.99968761	0.934904635	0.973348222
1070	ABC transporter permease [Idiomarina sp. A28L]	gi|494661694	14.84000015	YPVSHVTGLR	20.11572	18.02117	19.33564	18.9804	0.895874967	0.961220379	0.943560559
1071	aminodeoxychorismate lyase (pabC), partial [Caminibacter mediatlanticus]	gi|494738755	14.85000038	QIFTSSYKQHLK	15.79379	15.27143	14.91642	14.77365	0.966926241	0.944448419	0.93540879
1072	hypothetical protein BQ03360 [Bartonella quintana str. Toulouse]	gi|49473980	8.100000381	RRASC	18.47859	17.97489	17.26951	17.63831	0.972741427	0.934568601	0.954526833
1073	nitrogen regulatory protein p-II [Bartonella quintana str. Toulouse]	gi|49474369	22.54999924	GAKYVVDFLPK	16.32321	15.68665	15.42774	17.11129	0.961002768	0.945141035	1.048279719
1074	AsnC family transcriptional regulator [Wolbachia endosymbiont of Drosophila ananassae]	gi|494813305	17.28000069	SNHITQHGKGKATWYVI	17.30031	16.15762	16.25917	0	0.933949738	0.939819575	0
1075	ribonuclease [alpha proteobacterium BAL199]	gi|494943805	9.659999847	LAEQLAAAAMLEAIPQ	17.2558	14.61849	14.83324	15.41495	0.847163852	0.859608943	0.893319927
1076	molybdate ABC transporter permease [alpha proteobacterium BAL199]	gi|494943924	4.380000114	LDGGS	16.15665	15.69209	15.24056	16.20994	0.971246515	0.943299508	1.003298332
1077	sensory box histidine kinase/response regulator [alpha proteobacterium BAL199]	gi|494945985	15.93999958	LINLLTNAIKFSHR	17.70569	18.47567	15.18998	16.64945	1.043487715	0.857915167	0.940344601
1078	histidine ammonia-lyase [alpha proteobacterium BAL199]	gi|494948531	15.88000011	GAIADSAATVAR	0	16.33631	15.96109	16.07933		0.977031533	0.984269397
1079	GDSL family lipase [Sphingobium]	gi|494962131	17.28000069	RQIPVLLTGMLAAPNMGK	19.27966	15.62074	16.37475	17.55537	0.810218645	0.849327737	0.910564294
1080	alkylphosphonate utilization protein [Pelagibaca bermudensis]	gi|495067155	12.38000011	LIGTMRGARLAAE	18.26769	14.88341	14.66391	14.94545	0.814739576	0.802723826	0.818135736
1081	F0F1 ATP synthase subunit beta [Pelagibaca bermudensis]	gi|495072722	10.13000011	ARGGASEPFRTGIK	18.20234	16.58244	17.37952	0	0.911005948	0.954795922	0
1082	putative isomerase [Pelagibaca bermudensis]	gi|495077497	15.10000038	EPSGNR	19.18166	18.32743	18.04179	17.98736	0.955466315	0.940575008	0.937737401
1083	serine ammonia-lyase [Roseobacter sp. AzwK-3b]	gi|495086818	10.75	SGKTIAQMKK	16.63844	11.87044	15.00556	16.0647	0.713434673	0.901860992	0.9655172
1084	3-beta-hydroxy-delta(5)-steroid dehydrogenase [Roseobacter sp. AzwK-3b]	gi|495087110	13.22000027	AVMNGADAVVNCVGTFDRK	16.13972	15.6155	15.7958	15.33294	0.967519883	0.97869108	0.950012764
1085	hypothetical protein [Roseobacter sp. AzwK-3b]	gi|495087581	11.28999996	KPPKHA	16.20094	15.13066	15.1692	15.36003	0.933937167	0.936316041	0.948094987
1086	30S ribosomal protein S6 modification protein [Roseobacter sp. AzwK-3b]	gi|495088603	18.52000046	RAGSSEVE	16.91366	15.562	15.34209	16.64784	0.920084713	0.907082796	0.984283709
1087	serine/threonine dehydratase [Roseobacter sp. AzwK-3b]	gi|495092463	26.61000061	IVMEASCAPPLAAILK	17.64274	15.37196	15.70517	16.22708	0.871290967	0.890177489	0.919759629
1088	membrane-fusion protein [Xanthomonas fuscans]	gi|495237246	16.78000069	VHTTKQMDADTK	15.92946	13.95893	14.41591	15.78069	0.876296497	0.904984224	0.9906607
1089	UDP pyrophosphate synthase [Xanthomonas fuscans]	gi|495238136	12.25	ATESSSA	17.91451	16.15116	16.64257	16.69684	0.901568617	0.928999454	0.932028841
1090	NAD(P)H quinone oxidoreductase [Xanthomonas fuscans]	gi|495248409	5.730000019	MVLEM	15.58363	14.59569	14.64171	14.94542	0.936603988	0.939557087	0.959046127
1091	hypothetical protein [Rhodobacter sp. SW2]	gi|495303580	8.880000114	SARTDSMRDPLTSEEVKSILGI	16.93501	14.07837	14.08802	0	0.83131749	0.831887315	0
1092	DNA-binding protein [Rhodobacter sp. SW2]	gi|495307061	6.829999924	GAADV	16.58916	15.62307	15.33867	15.8513	0.941763778	0.924620053	0.955521557
1093	peptide ABC transporter substrate-binding protein [Rhodobacter sp. SW2]	gi|495308255	17.62999916	QGGVMLMTYK	19.93572	17.75078	19.31201	19.12813	0.890400748	0.968713947	0.959490302
1094	RNA methyltransferase [Rhodobacterales bacterium HTCC2255]	gi|495308753	14.60000038	AKSMAAGAGR	15.61288	15.79546	15.31329	15.9323	1.011694191	0.980811356	1.020458749
1095	alanine dehydrogenase [Rhodobacterales bacterium HTCC2255]	gi|495310363	11.85000038	SEMIVK	16.95421	0	15.93144	16.4379	0	0.939674571	0.969546797
1096	aspartate kinase [Reinekea blandensis]	gi|495320636	13.60999966	LAVLTGAMEAVIHK	17.50165	15.96874	16.07957	0	0.912413401	0.918745947	0

1097	copper resistance protein CopD [Reinekea blandensis]	gi|495320674	7.21999979	SVSRLLAFESAYER	16.99409	13.27261	13.82564	13.77253	0.781013282	0.813555771	0.810430567
1098	hypothetical protein [Methyloversatilis universalis]	gi|495333398	11.97999954	ELYWAPGQRTQGNHPDK	16.64487	15.52367	15.54455	15.87518	0.932639906	0.933894347	0.953758125
1099	hypothetical protein [Methyloversatilis universalis]	gi|495333668	4.800000191	GSAGGGGSPSGR	16.40601	14.82982	15.52464	0	0.903926061	0.946277614	0
1100	hypothetical protein [Methyloversatilis universalis]	gi|495333832	11.53999996	AAGGTGR	0	16.83052	16.34328	16.96931	0	0.971050211	1.008246329
1101	hypothetical protein [Methyloversatilis universalis]	gi|495335134	6.039999962	SVDAPEAVMLR	17.29372	15.05086	15.20557	15.48585	0.870307834	0.879253856	0.895460896
1102	MFS transporter [Methyloversatilis universalis]	gi|495335558	19.45000076	MRSTLQAPTR	18.64442	17.25594	17.55346	17.79171	0.925528389	0.941485978	0.9542646
1103	hypothetical protein [Methyloversatilis universalis]	gi|495335685	7.570000172	KNMLSIR	16.59433	0	15.47386	14.90032	0	0.932478744	0.897916336
1104	nitrate ABC transporter substrate-binding protein [Methyloversatilis universalis]	gi|495336171	12.55000019	IVAALGLGKDQCNIFLARK	18.49906	14.84139	15.41215	0	0.802278062	0.833131521	0
1105	oxidoreductase [Novosphingobium nitrogenifigens]	gi|495341967	26.23999977	GIGAATAKALAAAGAHVILVAR	17.79635	16.25777	16.54437	17.06147	0.913545193	0.929649619	0.958706139
1106	(dimethylallyl)adenosine tRNA methylthiotransferase [Novosphingobium nitrogenifigens]	gi|495345498	18.37999916	RIDSTPGA	17.95856	16.91154	16.74354	17.0689	0.941697998	0.932343128	0.950460393
1107	signal recognition particle [Novosphingobium nitrogenifigens]	gi|495346240	11.55000019	GAGMQVQDVNKLIKMHQEMAR	0	14.60623	14.58582	14.59352		0.998602651	0.999129823
1108	pilus assembly protein CpaB [Novosphingobium nitrogenifigens]	gi|495346956	31.93000031	ALLTGGSTPQAIAAAKVLDGPK	20.05986	16.24983	17.45084	19.20613	0.81006697	0.869938275	0.957440879
1109	exodeoxyribonuclease III [Rhodobacteraceae bacterium HTCC2150]	gi|495457444	15	DWDTADKGRR	17.85347	15.51441	15.45663	15.86728	0.868985693	0.865749347	0.888750478
1110	helicase UvrD [Rhodobacteraceae bacterium HTCC2150]	gi|495457785	10.19999981	ALAGDGGLDEDAAMAR	17.31629	15.85647	15.91013	16.76876	0.915696723	0.918795539	0.96838064
1111	beta-galactosidase [Rhodobacteraceae bacterium HTCC2150]	gi|495458010	20.18000031	SGNMSYLAGWPDTAAFQRILK	17.80137	18.09765	17.53857	15.935	1.016643663	0.985237091	0.895155822
1112	C4-dicarboxylate ABC transporter [Rhodobacteraceae bacterium HTCC2150]	gi|495461323	17.90999985	YLMTKIGGEPR	17.19848	15.40686	16.2892	16.618	0.895826841	0.947130212	0.96624818
1113	6-phosphogluconate dehydrogenase [Rhodobacteraceae bacterium HTCC2150]	gi|495462457	11.43999958	LINNFMGMTTAVTMSQAFAVAER	16.45971	13.13476	13.38634	14.53355	0.797994618	0.813279213	0.882977282
1114	polyketide synthase [Labrenzia alexandrii]	gi|495466513	13.22000027	AAAAIGSDK	15.58525	16.10716	0	14.32146	1.033487432	0	0.91891115
1115	hypothetical protein [Labrenzia alexandrii]	gi|495471121	11.39999962	KAVTLRFAQHTAHR	17.33398	14.68796	14.98044	16.23288	0.847350695	0.864223912	0.93647737
1116	Periplasmic serine protease [Roseobacter sp. SK209-2-6]	gi|495479253	21.97999954	GGMMAR	18.28346	17.20123	17.05999	17.60128	0.94080825	0.933083235	0.962688681
1117	hypothetical protein [Vibrionales bacterium SWAT-3]	gi|495493795	6.78000021	VFEIPELSASFDRGR	15.83732	14.38815	13.22946	14.51916	0.908496513	0.835334514	0.916768746
1118	hypothetical protein [Vibrionales bacterium SWAT-3]	gi|495494612	5.380000114	SAAFS	16.70222	15.76673	15.47055	15.84146	0.94399008	0.926257108	0.948464336
1119	RTX (repeat in toxin) cytotoxin [Vibrionales bacterium SWAT-3]	gi|495498650	15.22999954	FSMEEMVSK	16.7512	15.83693	15.63871	16.02576	0.945420627	0.933587444	0.956693252
1120	hypothetical protein [Roseobacter sp. GAI101]	gi|495500355	12.5	VVWLCNQPELIENYATVHPNLK	16.69021	13.62668	15.01659	15.04495	0.816447486	0.899724449	0.901423649
1121	hypothetical protein [Roseobacter sp. CCS2]	gi|495506394	21.73999977	MSPGVQSDDK	17.86849	17.0275	16.64266	17.09936	0.952934467	0.931397113	0.956956072
1122	endoglucanase [Roseobacter sp. CCS2]	gi|495509182	11.73999977	TMLASGGQDGAAAQQAAAGAR	0	15.72953	15.96979	15.826		1.015274455	1.00613305
1123	hypothetical protein [Limnobacter sp. MED105]	gi|495526791	12.22000027	KDLMIRTVGHSSFK	0	16.34137	16.86958	16.41811		1.032323483	1.004696057
1124	hypothetical protein [Roseovarius sp. TM1035]	gi|495555481	8.489999771	MEGANISASRMEGGCLSGVR	17.09773	15.67547	15.83216	16.20963	0.916815858	0.925980232	0.948057432
1125	phosphocarrier protein HPr [gamma proteobacterium HTCC5015]	gi|495558841	18.45000076	SIMGVMMLAASQGTTLK	18.88454	15.69715	17.04875	18.44462	0.831216964	0.902788736	0.976704754
1126	catabolite repression sensor kinase for PhoB	gi|495559416	12.56999969	LNEQDPTSSLMYVAAPLK	15.55358	13.76056	14.62344	15.7992	0.884719788	0.940197691	1.015791863
1127	cyanophycin synthetase [Congregibacter litoralis]	gi|495571579	30.84000015	RDDNLRGREPGEVPGLLK	17.44936	15.57352	15.62639	16.7789	0.892498063	0.895527974	0.961576814
1128	sterol carrier family protein, partial [Maritimibacter alkaliphilus]	gi|495607939	16.62000084	VSGDMSVAMK	15.63937	15.43153	14.41555	14.72154	0.986710462	0.921747487	0.941312853
1129	ABC transporter HlyB/MsbA family protein [Maritimibacter alkaliphilus]	gi|495609580	24.67000008	GGNLSGGQLQGLSLARLILR	0	17.58932	17.45044	18.05716		0.9921043	1.026597958
1130	T-DNA border endonuclease [Maritimibacter alkaliphilus]	gi|495611031	12.44999981	ADPLALYTSVMGRLWEDER	16.60291	14.41108	13.92326	14.22817	0.867985191	0.838603594	0.856968447
1131	ribonucleotide reductase [Candidatus Pelagibacter sp. HTCC7211]	gi|495820657	13.61999989	FGMPDRR	18.24504	16.11715	0	17.54563	0.88337159	0	0.961665746
1132	phosphoribosyl-AMP cyclohydrolase [Pseudovibrio sp. JE062]	gi|495823250	15.47999954	ADGFAPRADKK	17.78719	16.704	16.32537	16.64071	0.939102804	0.917816136	0.935544625
1133	imidazole glycerol phosphate synthase [Pseudovibrio sp. JE062]	gi|495824111	18.06999969	AEGLDWISGDVTAMKPANPELK	18.60927	16.90412	17.11911	17.30178	0.908370936	0.91992378	0.929739855
1134	alanine racemase [Rhodobacterales bacterium Y4I]	gi|495831631	22.71999931	SARTYLG	17.45231	16.87232	16.40027	16.42076	0.96676715	0.939719155	0.940893211
1135	cytochrome P450 [Rhodobacterales bacterium Y4I]	gi|495832644	15.60999966	FDPSRPPKVNK	0	16.16895	15.68197	16.03671		0.96988178	0.991821361
1136	hypothetical protein [Rhodobacterales bacterium Y4I]	gi|495833451	8.109999657	GKRWHGAGHGRD	17.00276	15.81656	15.63813	16.10776	0.930234856	0.919740677	0.947361487
1137	N-acylglucosamine 2-epimerase [Xanthomonas perforans]	gi|495851958	22.87000084	DRALVLAENMTR	17.04029	16.3214	15.90325	16.25006	0.957812338	0.933273436	0.953625789
1138	50S ribosomal protein L35 [Desulfovibrio]	gi|495957292	8.489999771	RAAAKRFQLTGSGK	15.71894	14.86781	0	15.39311	0.945853219	0	0.979271503
1139	peptide synthetase [Desulfovibrio sp. 3_1_syn3]	gi|495959127	17.56999969	AGMDGPHAR	15.97721	15.26463	14.75908	15.20707	0.955400223	0.923758278	0.951797592
1140	DNA-directed RNA polymerase subunit beta [Desulfovibrio]	gi|495960168	17.81999969	VGYMRDR	16.398	15.48655	15.15799	15.57163	0.944417002	0.924380412	0.94960544
1141	hypothetical protein [Rhodobacteraceae bacterium KLH11]	gi|496034675	5.480000019	TNAENN	16.06069	15.03227	14.89691	0	0.935966637	0.927538605	0
1142	putrescine transporter [Burkholderiales]	gi|496086948	12.31999969	CGMFSKRPGMGGYSEYAFGK	17.27935	16.21426	16.16841	0	0.938360529	0.935707072	0
1143	cell division protein FtsH [Enterobacteriaceae bacterium 9_2_54FAA]	gi|496090341	18.18000031	LSKKLGGLGGK	18.46094	15.81765	16.58704	18.06615	0.85681715	0.898493793	0.978614848
1144	hypothetical protein [Enterobacteriaceae bacterium 9_2_54FAA]	gi|496091148	14.38000011	KNNGGY	17.60695	16.69857	16.24949	16.49433	0.948407873	0.922902036	0.936807908
1145	phosphomethylpyrimidine synthase [Desulfonatronospira thiodismutans]	gi|496143890	0.5	GRAVLPK	16.432	13.89375	15.55824	15.62252	0.845530063	0.946825706	0.950737585
1146	methyl viologen-reducing hydrogenase [Desulfonatronospira thiodismutans]	gi|496146422	13.01000023	EVLLEMRKK	15.37156	14.86729	14.24959	13.99874	0.967194611	0.927010011	0.910690912
1147	phosphoribulokinase [Citreicella sp. SE45]	gi|496160849	11.30000019	TLPGAGQVDPAAR	17.63286	15.66032	16.34895	16.23785	0.888132725	0.927186514	0.920885778
1148	hypothetical protein [Citreicella sp. SE45]	gi|496163646	18.32999992	LLAQGGDR	17.84253	16.6059	16.61582	17.11409	0.930692004	0.931247979	0.959173951
1149	CRISPR-associated protein Cas3 [Rheinheimera sp. A13L]	gi|496174283	17.72999954	MNIMLVSQCSKNALIETR	17.94222	0	14.64738	14.61738	0	0.816363861	0.814691827
1150	TetR family transcriptional regulator [Rheinheimera sp. A13L]	gi|496174817	16.62999916	NQVLTAAADCFRRK	16.9175	16.26308	15.71743	0	0.961316979	0.929063396	0
1151	4-hydroxy-3-methylbut-2-en-1-yl diphosphate synthase [Rheinheimera sp. A13L]	gi|496175995	9.649999619	MSGFYLKGQRQK	16.2881	14.94337	15.2107	15.34494	0.917440954	0.93385355	0.942095149
1152	NAD synthetase [Rheinheimera sp. A13L]	gi|496176041	17.18000031	QSAVGPK	18.06725	16.7654	16.85276	17.22804	0.927944208	0.932779477	0.953550762
1153	lytic transglycosylase [Burkholderia sp. H160]	gi|496200725	6.059999943	FSDGNMFNPR	16.14903	15.50391	14.85519	15.12226	0.96005209	0.919881256	0.936419091
1154	peptidase C39 [Burkholderia sp. H160]	gi|496202535	15.39999962	SVGAKR	19.36475	18.37496	17.75599	18.41961	0.948887024	0.916923276	0.95119276
1155	(Fe-S)-binding protein [Burkholderia sp. H160]	gi|496202558	16.54999924	TPLLDAK	17.65201	14.33363	14.25173	14.5138	0.81201121	0.807371512	0.82221798
1156	WbcU protein [Brucella]	gi|496222337	9.539999962	GLGVKANK	17.44991	16.49043	16.0965	16.5051	0.945015189	0.922440288	0.945855881
1157	peptidase [Acinetobacter]	gi|496226906	17.36000061	AIKNNGLRAK	17.27568	15.75658	16.36619	16.33106	0.912067137	0.947354315	0.945320821

1158	phosphotransacetylase [Pseudogulbenkiania ferrooxidans]	gi|496240498	11.84000015	HAAVDK	18.02118	17.05417	16.7283	17.3122	0.946340362	0.92825775	0.960658514
1159	Mu P family protein [Pseudogulbenkiania ferrooxidans]	gi|496241061	10.52999973	IDAEMLIASVTYLK	18.2592	14.00718	15.79937	16.12479	0.767129995	0.865282707	0.883104955
1160	excinuclease ABC subunit B [Pseudogulbenkiania ferrooxidans]	gi|496241877	9.399999619	VAMMDEKALAK	16.28582	15.35039	15.04296	15.45961	0.942561689	0.92368453	0.949268136
1161	cyclase [Luminiphilus syltensis]	gi|496310109	18.32999992	TVLDAVALVKQGK	16.98693	13.92628	16.56954	16.54278	0.819823241	0.975428756	0.973853427
1162	general secretion pathway protein F [gamma proteobacterium NOR5-3]	gi|496314005	8.960000038	AEAANSSLR	17.05191	15.26326	14.99289	15.46111	0.895105592	0.87924989	0.906708398
1163	hypothetical protein [gamma proteobacterium NOR5-3]	gi|496315030	12.81999969	QALLKDQEKTP	15.92382	14.99568	14.76766	15.21118	0.941713735	0.927394306	0.955246919
1164	polyphenol oxidase [Thalassiobium sp. R2A62]	gi|496448808	25.95000076	LIAAISM	21.27561	20.4833	20.03035	20.03301	0.962759705	0.941470068	0.941595094
1165	hypothetical protein [Neisseria sp. oral taxon 014]	gi|496466164	9.149999619	KNGGFGGAAYSITPSKLAK	17.28599	0	15.93767	15.47635	0	0.921999261	0.895311752
1166	hypothetical protein [Silicibacter sp. TrichCH4B]	gi|496468242	1.350000024	TCASK	0	15.2673	14.62609	15.14984		0.958001087	0.992306433
1167	hypothetical protein [Silicibacter sp. TrichCH4B]	gi|496469558	1.649999976	QVASSM	16.09007	14.75934	14.67099	15.62255	0.917294953	0.911803988	0.97094357
1168	permease [Aurantimonas manganoxydans]	gi|496500696	8.770000458	AGGLPWISLVLAFSFGTYGLLR	16.19951	15.58625	15.66901	14.82822	0.962143299	0.967252096	0.915349909
1169	hypothetical protein [Aurantimonas manganoxydans]	gi|496501144	9.649999619	AADIIPGADNMAVAAYVR	17.68152	16.20064	16.44962	16.70957	0.916247019	0.930328388	0.945030178
1170	xanthine permease [Aurantimonas manganoxydans]	gi|496502725	12.94999981	MTQKTAAPLSGMIYR	17.28557	15.98071	17.32521	16.91282	0.924511601	1.002293242	0.97843577
1171	hypothetical protein [Ralstonia]	gi|496531392	30.55999947	MPDGGLLAEGKAAYDHGK	16.80322	0	15.30277	15.99674	0	0.910704615	0.952004437
1172	cyclic nucleotide-binding protein [Afipia sp. 1NLS2]	gi|496697951	12.13000011	LEAQVAALSGKSAS	17.55932	15.16973	15.11881	15.69719	0.863913295	0.861013411	0.893952044
1173	peptidyl-prolyl cis-trans isomerase [Ahrensia sp. R2A130]	gi|497082027	10.22999954	VIDGFMAQGGDPTGSGMGGSK	0	16.44901	15.69883	15.35303		0.954393608	0.933371066
1174	transcriptional regulatory protein [Ahrensia sp. R2A130]	gi|497086702	14.76000023	EDMQIDWLAK	17.87313	13.44117	15.93849	16.37993	0.752032241	0.891757068	0.916455596
1175	molecular chaperone Hsp70 [Roseibium sp. TrichSKD4]	gi|497092177	2.420000076	GTTYSSMGK	15.4912	14.81009	14.29519	14.87869	0.956032457	0.922794232	0.960460778
1176	uronate isomerase [Roseibium sp. TrichSKD4]	gi|497093880	22.59000015	DWFMN	17.27657	16.31341	15.71347	16.35573	0.944250508	0.909524865	0.946700068
1177	conserved hypothetical protein [Roseibium sp. TrichSKD4]	gi|497095797	2.839999914	AVNGKK	16.34414	15.21563	15.03967	0	0.930953235	0.920187296	0
1178	hypothetical protein [Roseibium sp. TrichSKD4]	gi|497098077	15.85000038	NTPQALAS	18.03078	17.02484	16.623	17.13838	0.944209846	0.921923511	0.950506855
1179	secretion activator protein [Roseibium sp. TrichSKD4]	gi|497098571	15.01000023	ALEADTGAVAVVAEAAK	17.97448	15.08129	15.12416	15.63156	0.839039015	0.841424063	0.869652975
1180	Nudix-like NDP and NTP phosphohydrolase YmfB [gamma proteobacterium IMCC3088]	gi|497261167	17.12000084	GQIYPLTMLAHYES	16.86554	0	15.33959	16.08749	0	0.909522612	0.953867472
1181	B12-dependent methionine synthase [gamma proteobacterium IMCC3088]	gi|497261208	15.97999954	GDGAATKK	15.70518	14.51088	14.13151	0	0.923955026	0.899799302	0
1182	microcin C ABC transporter permease YejB [Vibrio caribbenthicus]	gi|497287196	21.79999924	ADDTGSGYKGSR	17.67239	16.16026	16.67635	16.82567	0.914435456	0.943638636	0.952087975
1183	RpiR family transcriptional regulator [Oxalobacteraceae bacterium IMCC9480]	gi|497351546	11.78999996	SAQVSEPTVVR	16.5504	15.01885	16.35895	16.54592	0.907461451	0.988432304	0.999729312
1184	poly D(-)-3-Hydroxybutyrate depolymerase [Oxalobacteraceae bacterium IMCC9480]	gi|497352756	20.17000008	DPMLPK	17.46578	16.5995	16.26908	16.48425	0.950401299	0.931483163	0.943802682
1185	putative minor tail protein [Oxalobacteraceae bacterium IMCC9480]	gi|497353275	7.449999809	VIKGAAK	15.92616	14.66015	15.0149	15.40361	0.920507517	0.94278219	0.967189203
1186	urea ABC transporter ATP-binding protein [Oxalobacteraceae bacterium IMCC9480]	gi|497353788	8.020000458	TTLFKTLMGVLPK	17.85931	15.67427	16.45597	16.45223	0.877652608	0.921422496	0.921213082
1187	succinate dehydrogenase flavoprotein subunit [gamma proteobacterium IMCC1989]	gi|497354190	12.21000004	MAIRTVSFDGIVIGGGGAGMR	0	15.71915	15.92478	16.59999		1.013081496	1.056036109
1188	hypothetical protein [gamma proteobacterium IMCC1989]	gi|497354943	26.61000061	LEDIMASYAPK	16.54516	16.00679	15.54842	15.5392	0.967460575	0.9397564	0.939199137
1189	protein-L-isoaspartate O-methyltransferase [gamma proteobacterium IMCC1989]	gi|497355348	32.22000122	MTEILIAATSKRDK	19.19563	13.4555	12.59584	15.29205	0.700966835	0.656182683	0.796642257
1190	gamma-glutamyltransferase [Ahrensia sp. R2A130]	gi|497443883	12.10000038	DHDVCGMGPPSSGALTVGQILK	16.33956	15.65913	14.6604	15.81614	0.958356896	0.897233463	0.96796609
1191	5-keto-4-deoxy-D-glucarate aldolase [Ahrensia sp. R2A130]	gi|497444490	4.079999924	EMAAS	16.53497	0	15.27083	15.65238	0	0.923547488	0.946622824
1192	arginase [Nitrobacter sp. Nb-311A]	gi|497484078	15.80000019	TNMTGNPK	13.74401	14.10872	11.34886	12.81407	1.026535924	0.825731355	0.932338524
1193	probable chemotaxis protein [Nitrobacter sp. Nb-311A]	gi|497485397	24.44000053	VREMMESVAAGSEELNTSVR	19.16863	15.93376	15.82222	16.01023	0.831241461	0.825422578	0.835230791
1194	hypothetical protein [Oceanicaulis sp. HTCC2633]	gi|497486286	21.01000023	IMKEAGAR	18.7987	17.62495	16.64846	17.77193	0.937562172	0.885617622	0.945380798
1195	alpha/beta hydrolase [Oceanicaulis sp. HTCC2633]	gi|497488512	3.720000029	GGMPR	17.0504	16.40352	15.84479	16.3548	0.962060714	0.929291395	0.959203303
1196	hypothetical protein [Oceanicola batsensis]	gi|497490847	8.920000076	VTGVEVTTEGETR	0	17.69884	16.95712	16.10694		0.958092169	0.910056252
1197	1-deoxy-D-xylulose-5-phosphate synthase [Oceanicola batsensis]	gi|497492346	22.35000038	GEGAGVDMPDKGVPLEIGK	17.05615	16.58111	0	16.12419	0.972148463	0	0.945359299
1198	cell wall associated biofilm protein [Oceanicola batsensis]	gi|497493217	18.48999977	DTGGAIFK	17.63412	16.61225	16.36392	0	0.942051546	0.927969187	0
1199	transposase [Roseobacter sp. MED193]	gi|497497092	4.130000114	GGVGV	17.51828	17.19941	16.30768	16.97966	0.981797871	0.930895042	0.969253831
1200	hypothetical protein [Roseovarius nubinhibens]	gi|497498065	3.349999905	EALGMG	18.36981	16.6689	16.98196	17.48354	0.907407317	0.924449409	0.951753992
1201	L-aspartate dehydrogenase [Roseovarius nubinhibens]	gi|497498564	16.75	SSALAAMSAVAAIER	17.42258	16.44238	15.77461	16.66492	0.943739676	0.905411828	0.956512755
1202	ectoine synthase [Roseovarius nubinhibens]	gi|497498665	7.099999905	MIVRDFNK	17.33101	15.36335	15.59462	16.18461	0.886465936	0.899810225	0.933852672
1203	3,4-dihydroxy-2-butanone 4-phosphate synthase [Roseovarius sp. 217]	gi|497501393	22	LAGEMEDEDAPR	16.74569	15.69264	15.74839	16.17104	0.937115162	0.940444377	0.965683707
1204	acetyl-CoA carboxylase subunit alpha [Roseovarius sp. 217]	gi|497505766	9.220000267	IDSTPFARTAHLRRAK	0	14.86127	14.13744	14.47656		0.951294203	0.974113249
1205	alpha-glucuronidase [Sphingomonas sp. SKA58]	gi|497507292	10.06000042	GKGSTVAKVIDGSLDGHK	17.80652	15.69659	15.22548	13.14031	0.881508009	0.855050847	0.737949358
1206	CBS [Sphingomonas sp. SKA58]	gi|497507681	19.70000076	AEGSPK	0	16.80482	16.14251	16.90678		0.960588093	1.006067307
1207	MULTISPECIES: MFS transporter [Sphingomonadaceae]	gi|497509504	5.460000038	ASGDA	16.65398	15.62899	15.44641	0	0.938453751	0.927490606	0
1208	transposase [Sphingomonas sp. SKA58]	gi|497509822	18.98999977	ALAMIVEAHAALPMAAF	18.47252	15.27923	15.17196	15.74597	0.827132952	0.821325948	0.852399673
1209	monooxygenase [Sulfitobacter sp. NAS-14.1]	gi|497512764	8.539999962	AAAGNAAR	15.08419	14.61656	11.24241	14.83881	0.969131256	0.745310819	0.983732637
1210	methylase [Sulfitobacter sp. NAS-14.1]	gi|497512841	15.32999992	AFLTMIER	17.12625	15.92339	16.16995	16.30661	0.929765127	0.94416174	0.952141304
1211	DNA-binding protein [Sulfitobacter sp. NAS-14.1]	gi|497513198	13.93999958	ATPDGIK	17.11281	14.98818	15.8266	15.64706	0.875845638	0.924839345	0.91434779
1212	molecular chaperone DnaJ [Marinomonas sp. MED121]	gi|497518908	7.670000076	LLGIKENPTKSEIK	15.25097	13.69816	14.94355	15.4899	0.89818287	0.979842594	1.015666544
1213	hypothetical protein [Marinomonas sp. MED121]	gi|497519046	16.45999908	GYAKKLVLIIPKNDPSVSLSK	16.99395	15.79475	15.97688	16.10824	0.92943371	0.940151054	0.947880863
1214	acetyltransferase [Marinomonas sp. MED121]	gi|497520385	25.59000015	VNGKYQSILTMAKEIV	17.40895	15.68334	16.98482	16.41482	0.900877997	0.975637244	0.942895465
1215	hydrolase, alpha/beta fold family protein [Vibrio sp. MED222]	gi|497531820	9.109999657	TLSQGPK	16.20599	15.88558	15.10212	15.53059	0.980228915	0.931885062	0.958324052
1216	hypothetical protein [Mariprofundus ferrooxydans]	gi|497535351	30.45999908	NVPEHTNSLQVGGCLFSQPK	21.95913	18.32658	18.929	18.93794	0.83457678	0.862010471	0.862417591
1217	membrane protein [Mariprofundus ferrooxydans]	gi|497535814	12.65999985	DNFSPSLAMGMAGK	17.14273	14.8581	14.81324	15.2588	0.866728928	0.864112075	0.890103268
1218	NADH dehydrogenase subunit D [beta proteobacterium KB13]	gi|497538602	16.26000023	EDMESMIHHFK	16.84427	16.12657	15.61117	16.21338	0.957392039	0.926794097	0.96254572

1219	cytidyltransferase [Rubrivivax benzoatilyticus]	gi|497542338	1.529999971	SRGGASS	17.32543	14.81329	0	15.35663	0.855002733	0	0.886363571
1220	membrane protein [Rubrivivax benzoatilyticus]	gi|497542376	8.380000114	ASGPKK	16.89799	15.25695	15.93673	16.22862	0.902885491	0.943113944	0.960387596
1221	23S rRNA methyltransferase [Burkholderia thailandensis]	gi|497576627	17.02000046	EGALAVSKALAEIAQSGEGPLPAR	18.20573	15.86046	17.36752	17.73102	0.871179568	0.953959001	0.973925242
1222	dTDP-4-rhamnose reductase [Desulfotalea psychrophila LSv54]	gi|51246074	18.32999992	RPGNSQLDTSK	16.40239	15.42387	15.39402	15.7119	0.94034284	0.938522984	0.957903086
1223	hypothetical protein DP2661 [Desulfotalea psychrophila LSv54]	gi|51246513	12.67000008	LAIDSLLEKRSATELAK	18.46325	15.63336	16.21241	16.92435	0.846728501	0.878090802	0.916650644
1224	cell division protein [Wolbachia endosymbiont of Asobara tabida]	gi|51847978	19.20999908	TLGTKPAEQVS	18.04278	17.35082	0	17.27241	0.961648925	0	0.957303143
1225	LysR protein [Mannheimia succiniciproducens MBEL55E]	gi|52426207	16.35000038	AIVNSLV	16.86782	15.75354	0	15.98683	0.933940485	0	0.947770963
1226	hypothetical protein BMAA1969 [Burkholderia mallei ATCC 23344]	gi|53716130	25.55999947	ARGGRAVRR	15.68523	14.65778	14.66299	14.95156	0.934495701	0.93482786	0.953225423
1227	Ser/Thr protein phosphatase family protein [Burkholderia mallei ATCC 23344]	gi|53716592	10.64000034	PRPILR	16.85533	15.90969	15.39685	15.71133	0.943896678	0.913470694	0.932128294
1228	hypothetical protein BPSL1153 [Burkholderia pseudomallei K96243]	gi|53718788	7.460000038	AEVSRG	16.35181	12.88611	14.65286	15.07624	0.788054044	0.896100187	0.921992122
1229	4'-phosphopantetheinyl transferase [Burkholderia pseudomallei K96243]	gi|53720031	4.300000191	SGAAS	15.75244	14.69129	10.59075	14.65986	0.932635833	0.672324415	0.930640586
1230	elongation factor G [Burkholderia pseudomallei K96243]	gi|53720824	37.16999817	AEVPLSEMFGYSTSLR	17.11984	11.5277	13.44638	13.8241	0.673353256	0.785426733	0.807490023
1231	molecular chaperone GroEL [Burkholderia pseudomallei K96243]	gi|53721514	15.18000031	AAVEEGIVPGGGVALIR	15.64376	16.32345	14.25773	14.56746	1.043447995	0.911400456	0.931199405
1232	hypothetical protein MCA0201 [Methylococcus capsulatus str. Bath]	gi|53802631	5.289999962	GSADAGDMEAVVR	16.30125	15.90358	14.88166	15.22484	0.975604938	0.912915267	0.933967641
1233	hypothetical protein MCA0069 [Methylococcus capsulatus str. Bath]	gi|53802728	19.11000061	VRGMDAVVPAMPPPR	16.33303	0	17.16991	15.79266	0	1.051238503	0.966915508
1234	hypothetical protein lpp2016 [Legionella pneumophila str. Paris]	gi|54297961	15.86999989	ESQVLDEAKLSK	18.97064	16.4648	17.20857	0	0.86790957	0.907115943	0
1235	hypothetical protein G157_07140 [Campylobacter coli CVM N29710]	gi|543941344	17.88999939	ITLIAFSMGVCVASRVLK	0	15.51265	15.99115	16.41375		1.030845794	1.058088077
1236	acetoin(diacetyl) reductase [Idiomarina loihiensis L2TR]	gi|56459900	13.47000027	SIQNKVALVTGAGQGIGR	18.58572	17.03527	17.26536	0	0.916578427	0.928958362	0
1237	bifunctional aspartate kinase II/homoserine dehydrogenase II [Idiomarina loihiensis L2TR]	gi|56461566	16.96999931	LSELDEPMTAMLDEAAK	17.73451	15.0358	14.83995	15.27457	0.847827202	0.836783762	0.861290783
1238	lipase [Aromatoleum aromaticum EbN1]	gi|56475455	13.81000042	MPLSSQAK	16.54409	14.84635	15.03994	15.92462	0.897380877	0.909082337	0.962556417
1239	hemin receptor , TonB-dependent outer membrane uptake protein [Aromatoleum aromaticum EbN1]	gi|56476606	14.14999962	GLKKESVVLLVDGMR	17.55438	17.01469	15.85485	16.80757	0.969256106	0.903184846	0.957457341
1240	quinohemoprotein amine dehydrogenase, 60 kDa subunit [Aromatoleum aromaticum EbN1]	gi|56476668	13.59000015	VGGGGGPIPK	16.49645	13.67928	13.85774	13.97443	0.829225682	0.840043767	0.84711741
1241	30S ribosomal protein S4 [Aromatoleum aromaticum EbN1]	gi|56477618	11.35000038	MGFGGSR	16.86243	16.26831	15.78929	16.44328	0.964766644	0.936359113	0.975142966
1242	hypothetical protein ebA6614 [Aromatoleum aromaticum EbN1]	gi|56479216	11.73999977	DELMALRDSMSSTLR	17.51283	14.81973	14.60453	15.35213	0.846221313	0.833933179	0.876621882
1243	hypothetical protein ebA7090 [Aromatoleum aromaticum EbN1]	gi|56479458	23.82999992	MTRAEFGAAMSKIDARFASVEGR	18.17633	15.54858	16.59923	16.85845	0.855430112	0.913233309	0.927494714
1244	branched-chain amino acid ABC transporter, ATP-binding protein [Ruegeria pomeroyi DSS-3]	gi|56696810	23.29000092	KSGPTTK	17.61347	16.86923	16.55889	16.42522	0.957745975	0.940126505	0.932537427
1245	periplasmic glucan biosynthesis protein MdoG [Ruegeria pomeroyi DSS-3]	gi|56697727	19.37999916	VIRTAMGDKTFGEGR	17.58096	14.6904	14.36892	0	0.83558577	0.817300079	0
1246	ribosomal RNA small subunit methyltransferase B, putative [Ruegeria pomeroyi DSS-3]	gi|56698200	3.289999962	AADGPE	16.79988	13.16431	15.32673	15.07497	0.783595478	0.912311874	0.897326052
1247	hypothetical protein CJE1639 [Campylobacter jejuni RM1221]	gi|57238489	7.659999847	HESVTPSVEEKNK	15.7496	15.7063	14.70982	0	0.997250724	0.933980546	0
1248	ATP synthase F0F1 subunit alpha [Gluconobacter oxydans 621H]	gi|58040602	13.89999962	EVSLLTGRPPGR	17.14751	0	15.75236	15.63411	0	0.918638333	0.911742288
1249	virulence regulator [Xanthomonas oryzae pv. oryzae KACC 10331]	gi|58582367	10.93000031	NDIAAYLGTSAARK	16.51813	16.72476	15.68404	16.15899	1.012509285	0.949504575	0.978257829
1250	chromosome partitioning protein ParA [Vibrio fischeri ES114]	gi|59714060	9.600000381	KILIMNSKGGAGK	15.9713	15.27715	0	15.15943	0.956537664	0	0.949166943
1251	IcmP [Legionella brunensis]	gi|61814359	7.869999886	IDFSAAK	16.78061	15.9909	14.79883	15.91546	0.952939136	0.881900598	0.948443471
1252	homogentisate 1,2-dioxygenase [Pseudomonas syringae pv. syringae B728a]	gi|66046555	9.520000458	IKPSAAHSR	17.43548	16.83458	16.10679	16.43628	0.965535793	0.923793896	0.942691569
1253	hypothetical protein PFL_1012 [Pseudomonas protegens Pf-5]	gi|70728394	12.68999958	VTRVDTGATAPLDDCRR	17.80526	0	14.6606	15.45832	0	0.823385898	0.868188389
1254	cytochrome P450 monooxygenase [Pseudomonas protegens Pf-5]	gi|70730359	20.5	AARPLAANIKIKDGFPVMPGK	17.27859	15.79052	15.96426	16.1837	0.913877811	0.923933029	0.93663314
1255	hydrolase transmembrane protein [Ralstonia eutropha JMP134]	gi|73537515	18.80999947	GDVYFASLAGDYIAR	16.80811	14.37906	15.49288	16.38818	0.855483454	0.921750274	0.975016227
1256	hypothetical protein Reut_B4705 [Ralstonia eutropha JMP134]	gi|73538530	6.690000057	LAASVPAGPASFMDPR	16.47778	15.10122	14.95021	15.63792	0.91645962	0.907295157	0.949030755
1257	signal peptide protein [Ralstonia eutropha JMP134]	gi|73541905	0.050000001	GKGKGPK	16.58065	15.46768	14.73144	15.35836	0.93287537	0.888471803	0.926282142
1258	heat shock protein DnaJ, N-terminal, partial [Ehrlichia canis str. Jake]	gi|73667414	1.919999957	DTLLG	17.32422	15.52147	15.69404	16.04328	0.895940481	0.90590168	0.92606074
1259	endo-beta-1,4-xylanase [Cellvibrio mixtus]	gi|757809	11.39000034	SGNSSIYIEK	15.81524	14.83121	14.6037	14.94242	0.937779635	0.923394144	0.94481146
1260	coproporphyrinogen III oxidase [Nitrosococcus oceani ATCC 19707]	gi|77166276	18.55999947	GAVDRY	21.48246	20.69679	0	20.27403	0.963427373	0	0.943748062
1261	aldehyde dehydrogenase [Pseudomonas fluorescens Pf0-1]	gi|77458488	16.59000015	ISFTGSTATGKLIARAGIETMK	15.64913	15.07735	14.37994	14.84969	0.963462506	0.918897089	0.948914732
1262	triacylglycerol lipase [Pseudomonas fluorescens Pf0-1]	gi|77458910	8.149999619	VIASKFYGLTHK	17.38027	14.96144	15.01247	15.63684	0.860828974	0.863765062	0.89968913
1263	anthranilate synthase, component II [Rhodobacter sphaeroides 2.4.1]	gi|77462549	5.579999924	AAVTA	16.1335	15.46433	14.81289	15.33847	0.958522949	0.918144854	0.95072179
1264	short chain oxidoreductase precursor [Xanthomonas campestris pv. vesicatoria str. 85-10]	gi|78045615	16.95000076	NAGVAQAMADLADK	18.50631	15.37444	16.86879	18.64017	0.830767452	0.911515586	1.007233209
1265	hypothetical protein XCV0617 [Xanthomonas campestris pv. vesicatoria str. 85-10]	gi|78046173	11.53999996	DQYQNASNTSLSGNALTR	17.78677	16.63452	16.22569	16.1613	0.935218705	0.912233643	0.908613537
1266	hypothetical protein XCV1554 [Xanthomonas campestris pv. vesicatoria str. 85-10]	gi|78047110	10.68999958	APSMRSTGCCCG	17.53895	0	16.50819	16.20269	0	0.941230233	0.923811859
1267	carboxynorspermidine decarboxylase [Desulfovibrio alaskensis G20]	gi|78355620	14.06999969	HVSMGIR	17.45529	16.70002	16.13199	16.31649	0.956731169	0.924189171	0.934759033
1268	glycine dehydrogenase subunit 2 [Desulfovibrio alaskensis G20]	gi|78356734	12.48999977	KTVFSQSVPGR	16.55619	15.58067	14.9273	15.69162	0.941078231	0.901614441	0.947779652
1269	heme biosynthesis protein [Desulfovibrio alaskensis G20]	gi|78358219	8.039999962	MAQTFTDTER	17.27175	15.57715	15.65089	16.08914	0.901886028	0.906155427	0.931529231
1270	chaperonin Cpn10 [Thiomicrospira crunogena XCL-2]	gi|78484690	19.52000046	AADNGSIIPMTVKVGDK	17.27387	15.30662	16.30406	0	0.886114113	0.94385682	0
1271	phage exclusion protein Lit [Thiomicrospira crunogena XCL-2]	gi|78484712	10.71000004	IQDYSAKSGYPEGQVLMK	17.16678	15.96961	16.30633	16.44275	0.930262402	0.94987703	0.957823774
1272	dATP pyrophosphohydrolase [Nitrosospira multiformis ATCC 25196]	gi|82701737	19.20999908	VFSPSNAEAIRR	19.17191	16.13833	17.83095	19.11734	0.841769547	0.930056004	0.997153648
1273	hypothetical protein amb0327 [Magnetospirillum magneticum AMB-1]	gi|83309426	16.57999992	ATLGPANDMPHTQK	19.71671	15.62798	17.13095	17.24107	0.792626153	0.868854388	0.874439498
1274	hypothetical protein amb0428 [Magnetospirillum magneticum AMB-1]	gi|83309527	9.899999619	FVCGAR	18.30504	16.9277	17.01823	17.71754	0.924756242	0.929701874	0.967905014
1275	polyferredoxin [Magnetospirillum magneticum AMB-1]	gi|83310158	17.28000069	MKAVGDFLHR	17.37759	16.5696	16.91087	16.6972	0.953503909	0.973142421	0.9608467
1276	ankyrin repeat-containing protein [Magnetospirillum magneticum AMB-1]	gi|83310794	8.039999962	GAAINDK	15.44571	14.00779	13.46217	14.0822	0.906904895	0.871579876	0.911722414
1277	putative alpha-isopropylmalate/homocitrate synthase family transferase [Magnetospirillum magneticum AMB-1	gi|83311229	3.039999962	LTAFGMTR	15.54923	14.85844	14.32871	14.82052	0.955574006	0.921506081	0.9531353
1278	Acyl-CoA synthetase (AMP-forming)/AMP-acid ligase II [Magnetospirillum magneticum AMB-1]	gi|83311600	18.48999977	EMAKKLNG	12.48951	15.27028	13.31195	14.38461	1.222648447	1.065850462	1.151735336
1279	membrane protease subunit stomatin/prohibitin-like protein [Magnetospirillum magneticum AMB-1]	gi|83312588	16.61000061	VNQLLLGSRMGGDVRGGGR	0	16.43013	16.79282	17.16711		1.022074688	1.044855397

1280	ATP:corrinoid adenosyltransferase [Magnetospirillum magneticum AMB-1]	gi|83313574	11.51000023	RVLEGFGELVTFR	18.19493	17.40234	16.876	16.77767	0.956438964	0.927511125	0.922106873
1281	anticodon-binding protein [Rhodospirillum rubrum ATCC 11170]	gi|83592514	14.60000038	GGTCAAHR	15.30294	11.68883	15.06996	16.00331	0.763829042	0.984775475	1.045767023
1282	malto-oligosyltrehalose trehalohydrolase [Rhodospirillum rubrum ATCC 11170]	gi|83593630	13.05000019	DLGVTCLEIMPIAEFSGR	0	16.76715	16.12538	13.0483		0.961724563	0.778206195
1283	lyso-ornithine lipid acyltransferase [Rhodospirillum rubrum ATCC 11170]	gi|83595098	8.229999542	TVAPPAGP	18.07823	9.77875	15.45164	18.06292	0.540913021	0.854709781	0.999153125
1284	aspartate racemase [Hahella chejuensis KCTC 2396]	gi|83643337	9.909999847	VAPEVERR	17.09422	15.35203	15.54621	16.00161	0.898083095	0.90944249	0.936083074
1285	phosphoglyceromutase [Hahella chejuensis KCTC 2396]	gi|83644207	17.77000046	GEDDEFVKATLIK	0	16.07054	15.68525	15.3555		0.976025074	0.955506162
1286	acetyl-CoA acetyltransferase [Hahella chejuensis KCTC 2396]	gi|83644219	11.67000008	KDGTVTAGNASSLNDGAAAVMLCSEE	16.92267	15.11932	16.17368	0	0.893435847	0.955740436	0
1287	hypothetical protein HCH_02698 [Hahella chejuensis KCTC 2396]	gi|83645475	12.60000038	AARRASGMACQNTK	16.92815	14.57443	14.44451	14.9159	0.860958226	0.853283436	0.881129952
1288	2-oxoglutarate dehydrogenase complex, dehydrogenase (E1) component subunit alpha [Hahella chejuensis K	gi|83646416	19.44000053	SAGRMTHG	19.13999	18.1235	17.41301	18.37215	0.946891822	0.909771113	0.959882947
1289	chemotaxis protein [Burkholderia thailandensis E264]	gi|83717148	16.14999962	HMTSIAHDDLSGDIGVDER	0	15.74411	16.09552	16.84187		1.022320093	1.069725123
1290	D-alanyl-D-alanine carboxypeptidase family protein [Burkholderia thailandensis E264]	gi|83719638	17.36000061	FLHSGLTKIGK	17.81483	15.17572	15.40817	16.20488	0.851858817	0.864906934	0.909628663
1291	LysR family transcriptional regulator [Burkholderia thailandensis E264]	gi|83721499	15.80000019	FMSTH	17.04081	0	15.67209	16.34297	0	0.919679874	0.959048895
1292	Chain A, Crystal Structure Of Nicotinic Acid Mononucleotide Adenylyltransferase From Pseudomonas Aerugin	gi|83753741	18.46999931	GSHMGKRIGLFGGTFDPVHIGHMR	15.67533	13.17615	12.93705	0	0.840566036	0.825312769	0
1293	2-octaprenyl-6-methoxyphenyl hydroxylase [Sodalis glossinidius str. 'morsitans']	gi|85059982	7.630000114	NLGLLAMAHIPSLR	16.95636	15.36409	0	16.37961	0.906096002	0	0.965986214
1294	soxZ [Paracoccus denitrificans]	gi|8517645	6.480000019	VPSSAK	17.52505	16.35458	0	16.46728	0.933211603	0	0.939642398
1295	hypothetical protein ELI_01500 [Erythrobacter litoralis HTCC2594]	gi|85373127	18.39999962	ELAVVVIGAR	17.4878	16.10463	16.60183	16.75785	0.920906575	0.949337824	0.958259472
1296	hypothetical protein ELI_10810 [Erythrobacter litoralis HTCC2594]	gi|85374989	23.62000084	RVLKWSHKDVEK	17.72984	15.89684	0	16.91828	0.896614972	0	0.954226321
1297	calcium-transporting ATPase [Syntrophus aciditrophicus SB]	gi|85858735	21.40999985	ESQIPSR	17.08254	16.46723	16.26638	16.28932	0.963980181	0.952222562	0.953565453
1298	cytoplasmic protein [Syntrophus aciditrophicus SB]	gi|85859871	8.550000191	ETYMSK	14.85811	14.25156	13.94125	14.47527	0.959177177	0.938292286	0.9742336
1299	exonuclease SbcC [Anaeromyxobacter dehalogenans 2CP-C]	gi|86156861	13.60000038	DEALAAARRAEADAGAALATAR	17.47246	15.65483	16.48983	0	0.895971718	0.94376121	0
1300	aminodeoxychorismate lyase [Anaeromyxobacter dehalogenans 2CP-C]	gi|86157657	14.14000034	RLRAGEYAFSGPLTPDQVLDK	17.36349	17.45474	15.9026	16.40986	1.00525528	0.915864265	0.945078438
1301	RND efflux transporter [Anaeromyxobacter dehalogenans 2CP-C]	gi|86160559	12.85000038	ARAAAARAAATR	15.915	14.76944	14.79706	15.49424	0.928020107	0.929755577	0.973562048
1302	hypothetical protein RHE_CH01002 [Rhizobium etli CFN 42]	gi|86356648	14.92000008	HEAGPMGRR	17.29363	16.13195	16.29985	16.70722	0.932826133	0.94253491	0.966090983
1303	urea or short-chain amide ABC transporter, permease [Rhizobium etli CFN 42]	gi|86358915	17.30999947	STMEAARAVMMLKSDSAIEDK	17.67157	17.09374	16.57049	17.44308	0.967301717	0.93769201	0.987070192
1304	Formyl transferase-like [Rhodopseudomonas palustris HaA2]	gi|86749978	2.74000001	GIAVPR	15.70942	16.47742	14.3985	15.09132	1.048887865	0.91655198	0.960654181
1305	twin-arginine translocation pathway signal [Rhodopseudomonas palustris HaA2]	gi|86750233	12.63000011	RGFLTLAAGATMAAPGILR	17.26082	15.31272	15.77288	15.83044	0.887137459	0.913796679	0.917131399
1306	microcin-processing peptidase 1 [Novosphingobium aromaticivorans DSM 12444]	gi|87198319	4.860000134	GASGFR	17.56491	16.79261	17.21644	16.77843	0.956031656	0.980161014	0.955224365
1307	hypothetical protein Saro_1622 [Novosphingobium aromaticivorans DSM 12444]	gi|87199639	17.12000084	ALTAGQARAMTAASPAGLDK	18.31146	15.88406	16.4176	16.87073	0.867438205	0.89657515	0.921320856
1308	cytochrome P450 [Novosphingobium aromaticivorans DSM 12444]	gi|87200263	0.159999996	AVTMK	15.75752	14.14883	13.91024	13.94576	0.897909696	0.882768354	0.885022516
1309	dihydroorotase [Novosphingobium aromaticivorans DSM 12444]	gi|87201074	9.649999619	AWIGDSGVMLR	16.71398	12.6221	16.00663	16.56354	0.755182189	0.95767914	0.990999152
1310	hypothetical protein APH_0043 [Anaplasma phagocytophilum str. HZ]	gi|88606722	11.57999992	TNTEFR	16.41715	15.56138	15.21606	15.4835	0.947873413	0.926839311	0.943129593
1311	hypothetical protein NSE_0504 [Neorickettsia sennetsu str. Miyayama]	gi|88608030	25.93000031	IYSIF	17.92869	17.11989	16.53828	17.23587	0.954887948	0.922447764	0.961356909
1312	methylmalonate-semialdehyde dehydrogenase [Jannaschia sp. CCS1]	gi|89054404	13.35000038	EHGKTIPDAKGDVQR	0	15.48517	16.24358	16.44115		1.048976537	1.048976537
1313	hypothetical protein Jann_1962 [Jannaschia sp. CCS1]	gi|89054453	15.31000042	RVVADPGAVAEGR	16.87583	16.18884	0	16.07812	0.959291484	0	0.952730621
1314	carbon monoxide dehydrogenase, large subunit [Jannaschia sp. CCS1]	gi|89054586	4.800000191	GMGCSR	16.70586	15.86788	15.40383	15.79585	0.949839158	0.92206148	0.945527498
1315	Serine-type D-Ala-D-Ala carboxypeptidase [Jannaschia sp. CCS1]	gi|89056340	7.340000153	GEERIIATVFGGRSSAWR	16.61171	14.09876	15.32482	15.35764	0.848724183	0.922531154	0.924506869
1316	tRNA (guanine-N(1)-)-methyltransferase [Rhodoferax ferrireducens T118]	gi|89900197	12.65999985	DFAAGNYRR	17.83187	15.67774	16.52699	16.95111	0.879197751	0.926823154	0.950607536
1317	MerR family transcriptional regulator [Rhodoferax ferrireducens T118]	gi|89901059	15.93000031	AVGDIPTMLLWINTPK	16.97292	14.61778	14.49638	15.24417	0.861241319	0.854088748	0.898146577
1318	hypothetical arginine/serine rich protein [Burkholderia pseudomallei]	gi|90018712	10.19999981	FLEMSAQPAR	15.62154	15.03515	14.53004	13.98449	0.962462728	0.930128528	0.895205594
1319	hypothetical protein Sde_3306 [Saccharophagus degradans 2-40]	gi|90022946	17.95000076	ENLHLAWVTIR	17.88813	0	16.39106	16.17473	0	0.916309307	0.904215812
1320	methylase/helicase [Rhodopseudomonas palustris BisB18]	gi|90425054	13.06000042	GADDPR	18.34611	17.39905	17.18938	17.70374	0.948378158	0.936949577	0.964986038
1321	transport system permease [Rhodopseudomonas palustris BisB18]	gi|90425727	1.350000024	TLGGGQA	15.86201	15.01704	14.54951	14.88907	0.946729954	0.917255127	0.93866225
1322	alpha/beta hydrolase fold protein [Rhodopseudomonas palustris BisB18]	gi|90425892	23.95000076	AVLIGAVPPIMVK	17.69091	16.45656	17.6414	17.10406	0.930226879	0.997201388	0.966827597
1323	30S ribosomal protein S20 [Rickettsia bellii RML369-C]	gi|91205851	6.190000057	IKTMN	0	15.14142	15.009	15.54297		0.991254453	1.02651997
1324	PhoH-like protein [Methylobacillus flagellatus KT]	gi|91775005	17.85000038	HAQPNAGNPGADKD	18.74486	15.82469	16.81957	16.90215	0.844214894	0.89728971	0.901695185
1325	cardiolipin synthetase 2 [Methylobacillus flagellatus KT]	gi|91775522	13.52000046	LESLFDAARDASGEISAK	16.61904	0	15.58714	15.8064	0	0.937908568	0.951101869
1326	cell division protein FtsZ [Methylobacillus flagellatus KT]	gi|91776615	10.39999962	VTMVATGLNGVASR	16.66907	14.17623	14.82149	15.37746	0.850451165	0.889161183	0.922514573
1327	putative type II and III secretion system outermembrane protein, secretin [Burkholderia xenovorans LB400]	gi|91777217	12.40999985	DMAGTVK	16.26014	14.95418	15.08591	15.32066	0.919683348	0.927784755	0.9422219
1328	tripartite tricarboxylate transporter(TTT) family, periplasmic ligand binding protein TctC [Burkholderia xenovora	gi|91777990	3.599999905	AGVEPV	18.58676	17.79675	17.48899	17.6793	0.957496089	0.940938066	0.951177074
1329	YD repeat-containing protein [Burkholderia xenovorans LB400]	gi|91778818	21.36000061	TTCEAK	18.27972	17.33892	17.05944	17.4816	0.948533129	0.933244054	0.9563385
1330	IclR family transcriptional regulator [Burkholderia xenovorans LB400]	gi|91779398	13.60999966	GITLGPK	17.78633	17.09928	16.70899	16.85985	0.96137202	0.939428764	0.947910558
1331	precorrin-4 C(11)-methyltransferase [Polaromonas sp. JS666]	gi|91788641	16.97999954	DTAAPGK	19.49136	18.85857	18.40544	18.36655	0.967534846	0.944286411	0.942291867
1332	LacI family transcriptional regulator [Polaromonas sp. JS666]	gi|91790198	24.36000061	QIYKGEMPSTK	16.78493	15.87872	15.66014	16.03926	0.946010499	0.932988103	0.955575031
1333	hypothetical protein Bpro_5416 [Polaromonas sp. JS666]	gi|91791218	11.43000031	GRPHSWCKACYTNSPSTAAKR	16.53022	0	14.01545	14.46178	0	0.847868328	0.874869179
1334	FAD linked oxidase-like protein [Shewanella denitrificans OS217]	gi|91793363	13.27999973	DEYHEILGKSRGDFEVK	17.77383	14.74662	14.67233	15.24809	0.829681616	0.825501876	0.857895569
1335	hypothetical protein Sden_2148 [Shewanella denitrificans OS217]	gi|91793502	15.86999989	AESVTSDIQNLRK	17.6423	0	16.4743	15.82132	0	0.933965526	0.896783299
1336	short-chain dehydrogenase/reductase SDR [Rhodopseudomonas palustris BisB5]	gi|91977792	22.68000031	DATMAAFGK	15.20829	14.51669	14.03498	14.64112	0.954524802	0.922850629	0.962706524
1337	2OG-Fe(II) oxygenase [Rhodopseudomonas palustris BisB5]	gi|91978060	10.64000034	HMMTPGGHAMSVAMTSCGRVGWVT	15.99351	14.87696	0	15.17006	0.930187307	0	0.948513491
1338	hypothetical protein Csal_0882 [Chromohalobacter salexigens DSM 3043]	gi|92113010	10.46000004	AVMFCVMDVSGSMTQGHKDIAK	15.90652	15.86084	14.68886	0	0.997128222	0.923449001	0
1339	ATP-dependent helicase HrpA [Chromohalobacter salexigens DSM 3043]	gi|92113593	12.96000004	LPIDPRLARMALAGAEQGSLR	16.69475	15.52769	15.80957	16.01708	0.930094191	0.946978541	0.959408197
1340	hypothetical protein Nham_1133 [Nitrobacter hamburgensis X14]	gi|92116711	14.69999981	STSDMLYLDSLYR	16.85867	14.08356	13.9482	14.48559	0.835389743	0.82736064	0.85923682

1341	potassium efflux system protein [Psychrobacter cryohalolentis K5]	gi|93006835	3.450000048	AAGIKK	16.44711	15.05571	15.17579	15.68432	0.915401551	0.92270253	0.953621639
1342	hypothetical protein Pcryo_2277 [Psychrobacter cryohalolentis K5]	gi|93007101	11.59000015	DLIPLLVHMVRDEEEK	18.10592	16.13454	16.47549	14.93878	0.89111959	0.909950447	0.825077102
1343	putative inner membrane protein translocase component YidC [Psychrobacter cryohalolentis K5]	gi|93007297	6.679999828	LAALKEEHGDDRMK	17.50245	14.62887	15.58727	16.96663	0.835818414	0.890576462	0.969386
1344	phosphoglycerate kinase [Cupriavidus metallidurans CH34]	gi|94309446	49.31999969	AFSIAGGGDTLAAIAK	20.49421	0	19.45442	18.40023	0	0.949264207	0.897825776
1345	Zinc-binding dehydrogenase superfamiy protein [Cupriavidus metallidurans CH34]	gi|94309869	35.18000031	AEIPLGDILR	18.37656	16.34377	17.50716	17.28963	0.889381364	0.952689731	0.940852368
1346	acetyl-CoA:acetoacetyl-CoA transferase, subunit alpha [Cupriavidus metallidurans CH34]	gi|94310098	20.21999931	AGGSGIPAFFTK	17.66991	16.39995	16.61245	17.08379	0.928128666	0.94015476	0.966829486
1347	pyruvate dehydrogenase subunit E1 [Cupriavidus metallidurans CH34]	gi|94310141	43.79999924	VQLLGSGTIFR	18.94513	16.18685	15.2172	16.8313	0.854406911	0.803224892	0.888423568
1348	outer membrane protein assembly factor,outer membrane protein, surface antigen OMA87 [Cupriavidus metall	gi|94310385	60.22999954	VEPGTVFGYLPVR	18.84388	0	14.16985	14.61264	0	0.751960318	0.775458133
1349	putative protease, membrane anchored [Cupriavidus metallidurans CH34]	gi|94310397	41.29000092	VAEEYVAAFGNLAK	18.66266	16.16318	16.74973	17.64311	0.866070539	0.897499606	0.945369524
1350	periplasmic L-asparaginase II [Cupriavidus metallidurans CH34]	gi|94310525	48.75	SPFGPLGYVVEGR	19.51149	17.23076	18.14935	18.74047	0.883108363	0.930187802	0.960483797
1351	aconitate hydratase [Cupriavidus metallidurans CH34]	gi|94311428	49.77999878	VDEIPFVVAR	19.17962	16.58219	16.92156	17.23733	0.864573438	0.88226774	0.89873157
1352	ribulose-phosphate 3-epimerase [Cupriavidus metallidurans CH34]	gi|94312107	58.38999939	IAPSILSADFAR	20.80664	19.42318	19.4767	19.72539	0.933508726	0.936080982	0.948033416
1353	cytochrome c family protein [Cupriavidus metallidurans CH34]	gi|94312404	5.559999943	GKGVMPPK	18.11598	17.43172	16.84505	17.31254	0.962228927	0.929844811	0.955650205
1354	squalene cyclase [Cupriavidus metallidurans CH34]	gi|94313328	22.80999947	LAAARASAMQTAK	17.50412	15.70142	16.62204	17.03609	0.897012818	0.949607292	0.973261724
1355	hypothetical protein Rmet_5653 [Cupriavidus metallidurans CH34]	gi|94314572	14.88000011	NRGPKK	15.58361	13.16054	14.60822	15.32056	0.844511638	0.93740924	0.983120086
1356	putative, partial [Methylobacillus flagellatus]	gi|945407	17.89999962	KTALVGP	17.37505	12.58976	16.88351	17.43563	0.724588418	0.971710009	1.003486609
1357	hypothetical protein LI0638 [Lawsonia intracellularis PHE/MN1-00]	gi|94987080	14.13000011	AHTDMIAIAAVTPH	17.13008	15.60823	15.38691	15.8655	0.911159201	0.898239238	0.926177811
1358	ATP phosphoribosyltransferase [Ruegeria sp. TM1040]	gi|99080345	0.430000007	SAAMK	16.62964	15.89594	15.43203	15.83332	0.955879983	0.927983408	0.952114417
1359	ABC transporter [Ruegeria sp. TM1040]	gi|99081642	13.68000031	IMLMDEPFSGLDNRLRDGIR	17.72833	15.15288	14.2822	0	0.854726869	0.805614516	0
1360	pyridoxine 5'-phosphate synthase [Ruegeria sp. TM1040]	gi|99082403	2.829999924	SGADA	15.86189	16.84096	14.9813	15.62014	1.061724675	0.944483917	0.984759067
1361	predicted primosomal protein N' (replication factor Y) - superfamily II helicase [uncultured marine gamma prote	gi|9971884	14.43999958	AGAIKK	16.32522	14.87897	15.17596	15.57748	0.911410076	0.929602174	0.954197248

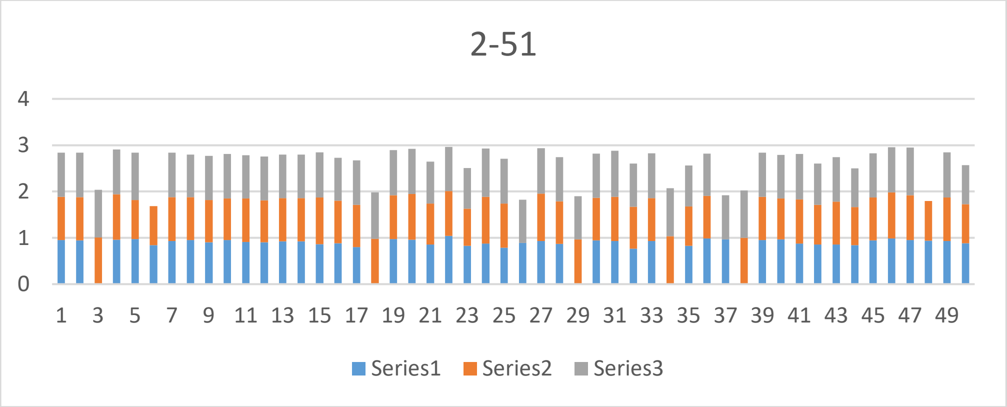

2-51
4
3
2
1
0
1 3 5 7 9 11 13 15 17 19 21 23 25 27 29 31 33 35 37 39 41 43 45 47 49
Series1
Series2
Series3

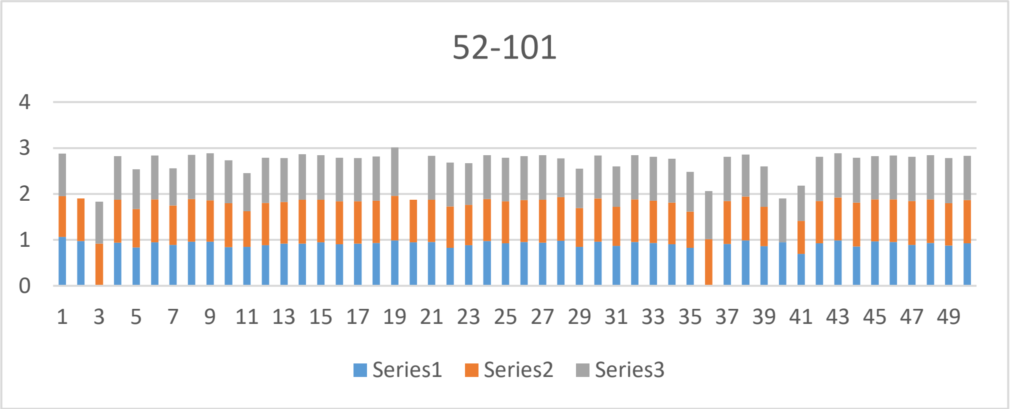
52-101
4
3
2
1
0
1 3 5 7 9 11 13 15 17 19 21 23 25 27 29 31 33 35 37 39 41 43 45 47 49
Series1 Series2 Series3

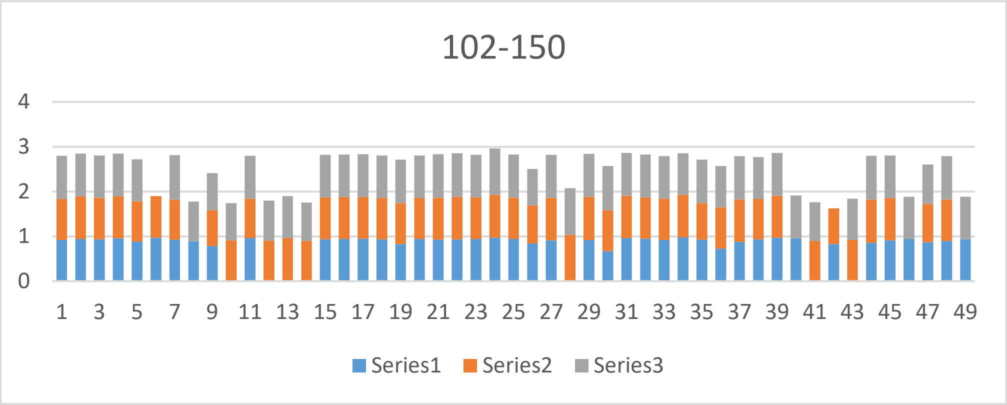
102-150
4
3
2
1
0
1 3 5 7 9 11 13 15 17 19 21 23 25 27 29 31 33 35 37 39 41 43 45 47 49
Series1 Series2 Series3

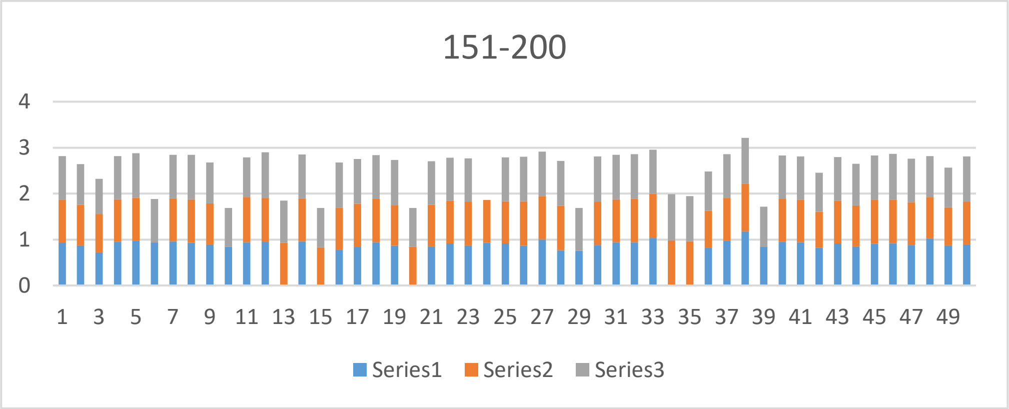
151-200
4
3
2
1
0
1 3 5 7 9 11 13 15 17 19 21 23 25 27 29 31 33 35 37 39 41 43 45 47 49
Series1 Series2 Series3

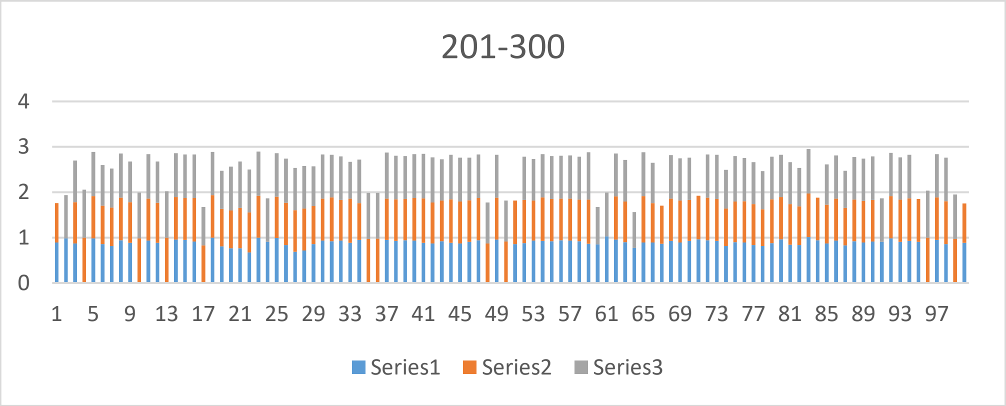
201-300
4
3
2
1
0
1 5 9 13 17 21 25 29 33 37 41 45 49 53 57 61 65 69 73 77 81 85 89 93 97
Series1 Series2 Series3

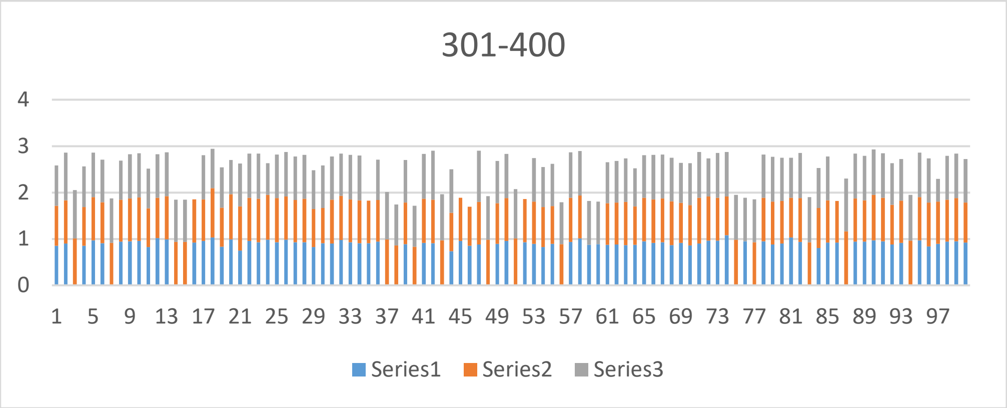
301-400
4
3
2
1
0
1 5 9 13 17 21 25 29 33 37 41 45 49 53 57 61 65 69 73 77 81 85 89 93 97
Series1 Series2 Series3

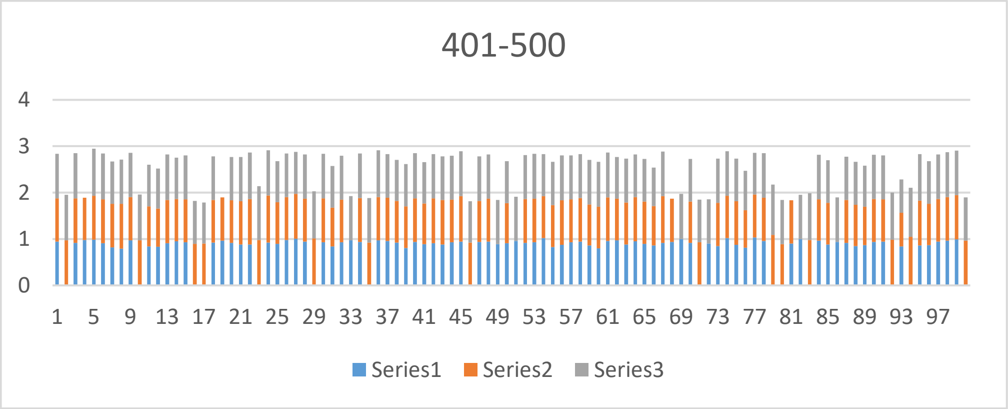

401-500
4
3
2
1
0
1 5 9 13 17 21 25 29 33 37 41 45 49 53 57 61 65 69 73 77 81 85 89 93 97
Series1 Series2 Series3

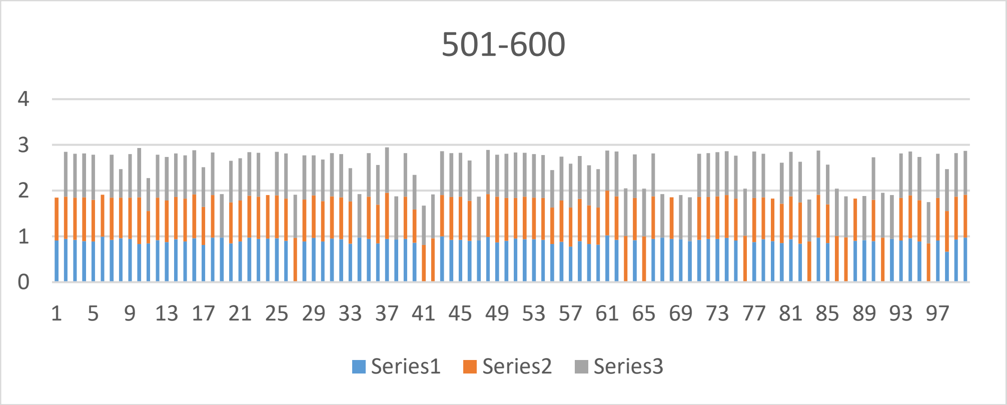
501-600
4
3
2
1
0
1 5 9 13 17 21 25 29 33 37 41 45 49 53 57 61 65 69 73 77 81 85 89 93 97
Series1 Series2 Series3

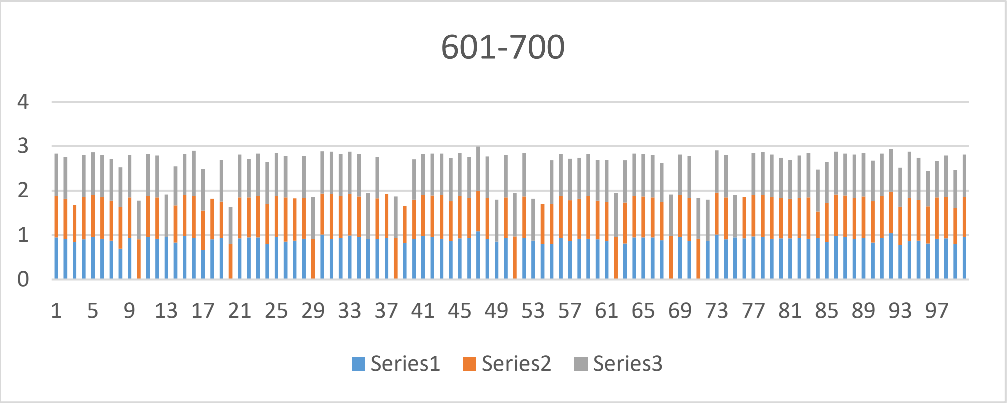
601-700
4
3
2
1
0
1 5 9 13 17 21 25 29 33 37 41 45 49 53 57 61 65 69 73 77 81 85 89 93 97
Series1 Series2 Series3

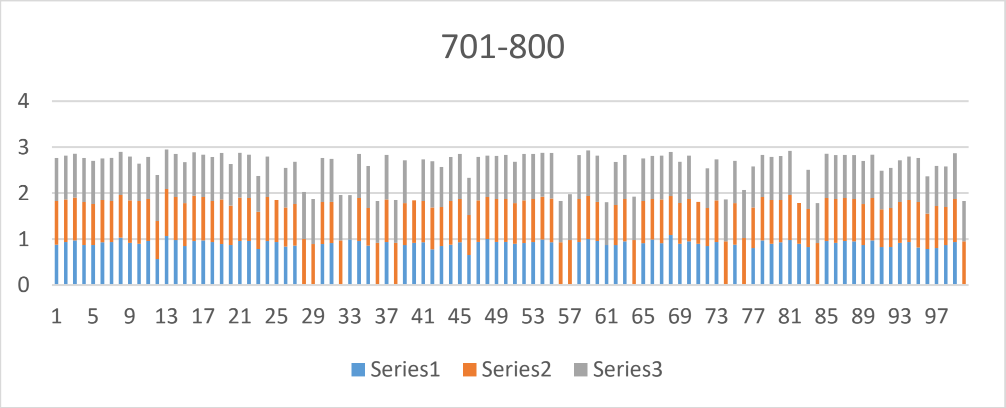

701-800
4
3
2
1
0
1
5
9
13
17
21
25
29
33
37
41
45
49
53
57
61
65
69
73
77
81
85
89
93
97
Series1
Series2
Series3

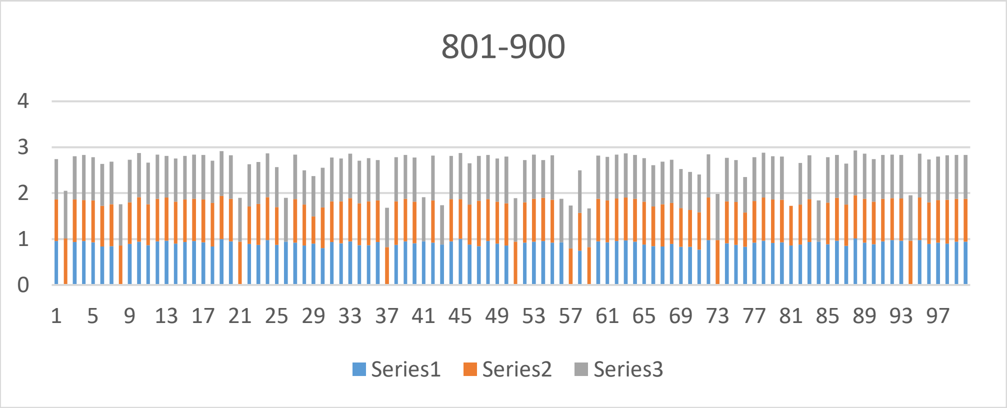
801-900
4
3
2
1
0
1 5 9 13 17 21 25 29 33 37 41 45 49 53 57 61 65 69 73 77 81 85 89 93 97
Series1 Series2 Series3

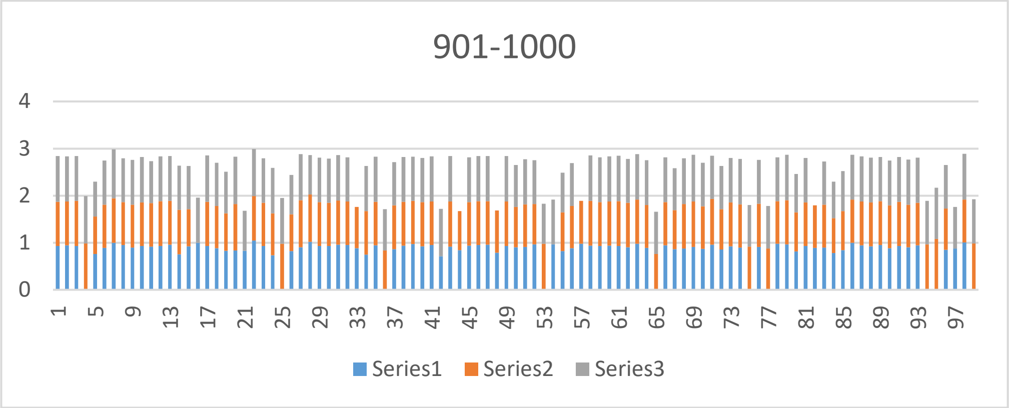

901-1000
4
3
2
1
0
1
5
9
13
17
21
25
29
33
37
41
45
49
53
57
61
65
69
73
77
81
85
89
93
97
Series1
Series2
Series3

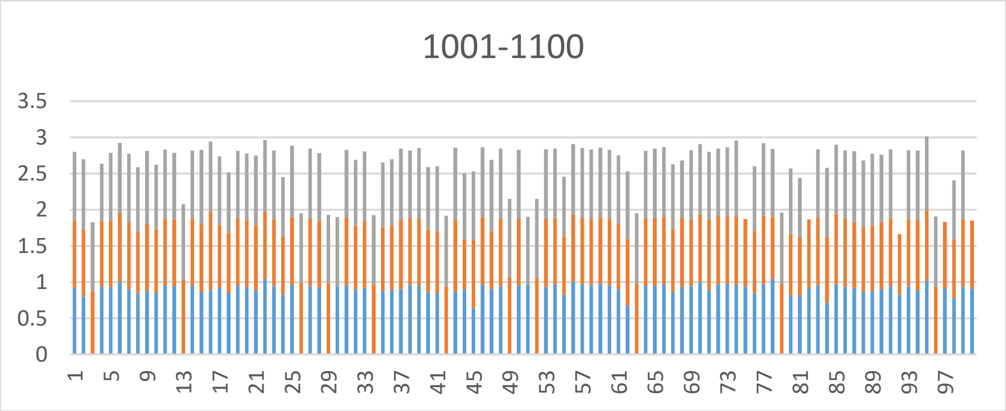
1001-1100
3.5
3
2.5
2
1.5
1
0.5
0
1
5
9
13
17
21
25
29
33
37
41
45
49
53
57
61
65
69
73
77
81
85
89
93
97

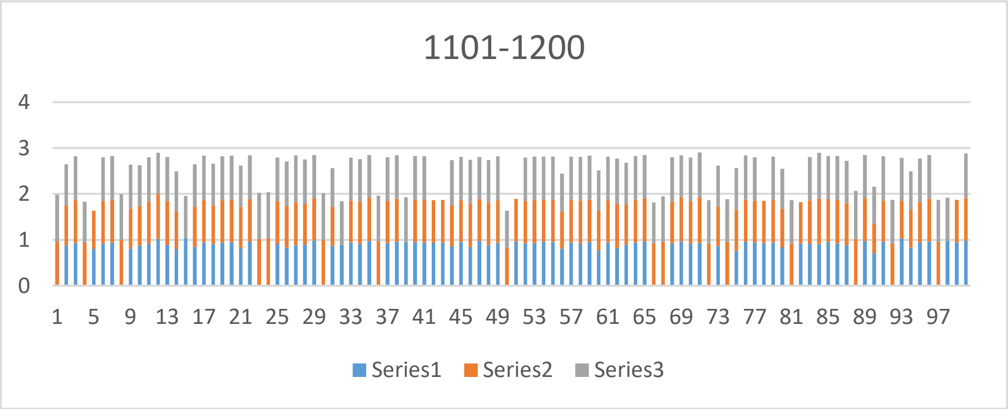

1101-1200
4
3
2
1
0
1 5 9 13 17 21 25 29 33 37 41 45 49 53 57 61 65 69 73 77 81 85 89 93 97
Series1
Series2
Series3

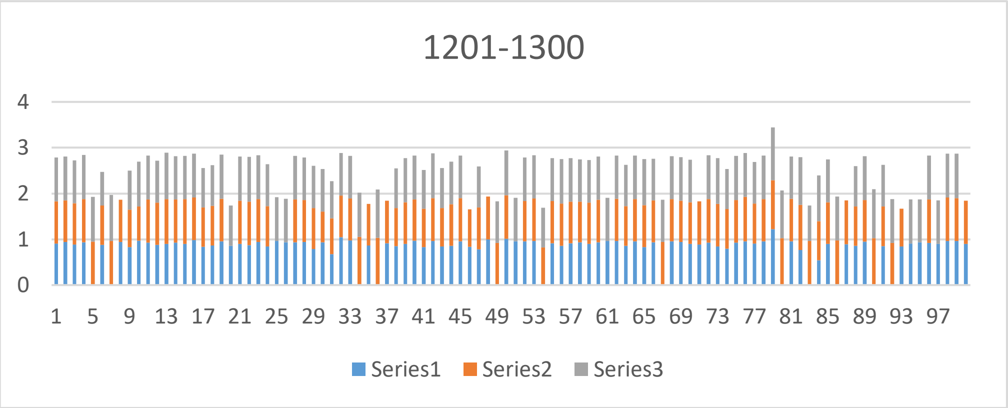
1201-1300
0
1
2
3
4
1 5 9 13 17 21 25 29 33 37 41 45 49 53 57 61 65 69 73 77 81 85 89 93 97
Series1 Series2 Series3

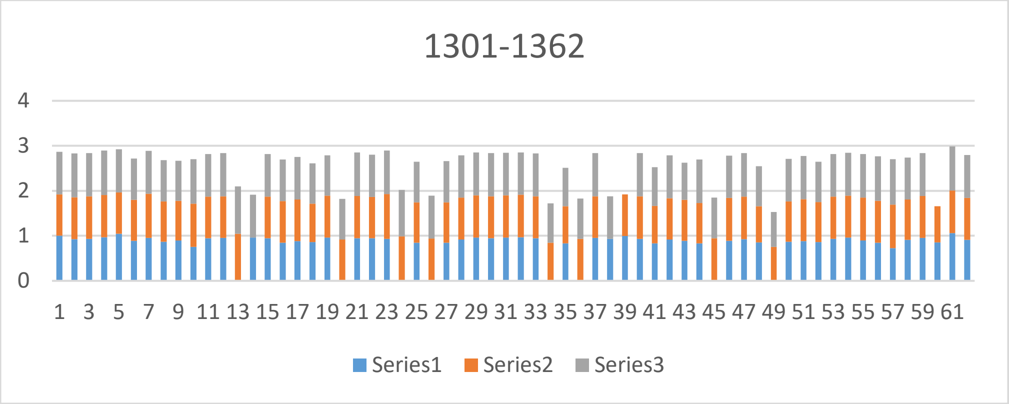
1301-1362
4
3
2
1
0
1 3 5 7 9 11 13 15 17 19 21 23 25 27 29 31 33 35 37 39 41 43 45 47 49 51 53 55 57 59 61
Series1
Series2
Series3

Table S2 : Analysis of the MEV program of 93 proteins having expression patterns in correlated with glucose or PHA formation.

Number	glucose	
1	Chain A, Heterotetrameric Sarcosine	Structure Of A Diflavin Metaloenzyme At 1.85 A Resolution
2	uridylate kinase	Herminiimonas arsenicoxydans]
3	PAS/PAC sensor hybrid histidine kinase	Magnetococcus marinus MC-1]
4	nitrogen regulatory protein p-II	Bartonella quintana str. Toulouse]
5	amino acid ABC transporter substrate-binding protein	Rhizobium etli CIAT 652]
6	2-oxoglutarate dehydrogenase, E2 component, dihydrolipoamide succinyltransferase	Wolbachia endosymbiont of Culex quinquefasciatus Pel]
Number	PHA	
1	hypothetical protein Tmz1t_0740	Thauera sp. MZ1T]
2	glycosyl transferase family protein	Burkholderia vietnamiensis G4]
3	signal recognition particle	Novosphingobium nitrogenifigens]
4	hypothetical protein RALTA_A0496	Cupriavidus taiwanensis LMG 19424]
5	urease accessory protein UreG	Bermanella marisrubri]
6	acetyl-CoA carboxylase subunit alpha	Roseovarius sp. 217]
7	elongation factor G domain-containing protein	Anaeromyxobacter sp. Fw109-5]
8	group 1 glycosyl transferase	Novosphingobium sp. PP1Y]
9	electron transport complex protein RnfG	Buchnera aphidicola str. Bp (Baizongia pistaciae)]
10	30S ribosomal protein S20	Rickettsia bellii RML369-C]
11	hypothetical protein DaAHT2_0024	Desulfurivibrio alkaliphilus AHT2]
12	hypothetical protein CCNA_01862	Caulobacter crescentus NA1000]
13	peroxidase	Serratia odorifera]
14	hypothetical protein CKO_02459	Citrobacter koseri ATCC BAA-895]
15	sensory box protein	Cellvibrio japonicus Ueda107]
16	polymerase	Citrobacter youngae]
17	hypothetical protein	Silicibacter sp. TrichCH4B]
18	TLDD protein-like protein	Neisseria meningitidis Z2491]
19	Fis family transcriptional regulator	Desulfovibrio vulgaris str. 'Miyazaki F']
20	RtcB protein	Teredinibacter turnerae T7901]
21	major facilitator superfamily protein	Methylobacterium nodulans ORS 2060]
22	type II secretion system protein E	Pectobacterium carotovorum subsp. carotovorum PC1]
23	methionyl-tRNA formyltransferase	Campylobacter curvus 525.92]
24	methylmalonate-semialdehyde dehydrogenase	Jannaschia sp. CCS1]
25	hypothetical protein G157_07140	Campylobacter coli CVM N29710]
26	malonyl CoA-ACP transacylase	Methylophaga aminisulfidivorans]
27	putative mercury transport protein MerC	Nitrosomonas eutropha C91]
28	beta-lactamase class C-like protein	Shewanella amazonensis SB2B]
29	protein YhiI	Pasteurella multocida subsp. multocida str. HN06]
30	succinate dehydrogenase flavoprotein subunit	gamma proteobacterium IMCC1989]
31	short-chain dehydrogenase	marine gamma proteobacterium HTCC2143]
32	endoglucanase	Roseobacter sp. CCS2]
33	chemotaxis protein	Burkholderia thailandensis E264]

34	amidase	Ralstonia eutropha H16]
35	preprotein translocase subunit SecA	Idiomarina baltica]
36	inorganic pyrophosphatase	Helicobacter mustelae 12198]
37	hypothetical protein	Vibrio mimicus]
38	lytic murein transglycosylase	Roseobacter denitrificans OCh 114]
39	AMP-dependent synthetase	Roseomonas cervicalis]
40	transposase IS116/IS110/IS902 family protein	Comamonas testosteroni CNB-2]
41	pH regulation protein F	delta proteobacterium MLMS-1]
42	flagellar scaffolding protein FlgD	Loktanella vestfoldensis]
43	phosphoglyceromutase	Hahella chejuensis KCTC 2396]
44	acyl-CoA dehydrogenase domain-containing protein	Methylobacterium radiotolerans JCM 2831]
45	hypothetical protein Nwat_2579	Nitrosococcus watsonii C-113]
46	septum site-determining protein minC	Thioalkalimicrobium cyclicum ALM1]
47	capsular exopolysaccharide family protein	Thioalkalivibrio sp. K90mix]
48	cytochrome P450	Rhodobacterales bacterium Y4I]
49	aldo/keto reductase	Escherichia coli E24377A]
50	GCN5-related N-acetyltransferase	Dickeya zeae Ech1591]
51	hypothetical protein	Oxalobacter formigenes]
52	conjugal transfer protein TrbC	Burkholderia ambifaria]
53	histidine ammonia-lyase	alpha proteobacterium BAL199]
54	adhesin	Proteus mirabilis]
55	hypothetical protein	Limnobacter sp. MED105]
56	pilin accessory protein PilO	Yersinia pseudotuberculosis IP 31758]
57	transmembrane anchor protein	Stenotrophomonas maltophilia K279a]
58	hypothetical protein	Erythrobacter sp. NAP1]
59	cobalamin biosynthesis protein cobD	Hyphomicrobium sp. MC1]
60	membrane protease subunit stomatin/prohibitin-like protein	Magnetospirillum magneticum AMB-1]
61	peptidyl-prolyl cis-trans isomerase	Ahrensia sp. R2A130]
62	integral membrane sensor signal transduction histidine kinase	Burkholderia sp. CCGE1002]
63	HrpA protein	Neisseria gonorrhoeae NCCP11945]
64	aromatic hydrocarbon degradation membrane protein	Sinorhizobium medicae WSM419]
65	superoxide dismutase	Burkholderia cenocepacia AU 1054]
66	polar amino acid ABC transporter inner membrane subunit	Mesorhizobium ciceri biovar biserrulae WSM1271]
67	flagellar hook protein	Grimontia hollisae]
68	malto-oligosyltrehalose trehalohydrolase	Rhodospirillum rubrum ATCC 11170]
69	CBS	Sphingomonas sp. SKA58]
70	hypothetical protein	Methyloversatilis universalis]
71	type VI secretion protein	Escherichia coli]
72	CarD family transcriptional regulator	Methylobacterium sp. 4-46]
73	extracellular solute-binding protein	Rhizobium leguminosarum bv. trifolii WSM1325]
74	4Fe-4S ferredoxin	Dickeya dadantii Ech703]

75	beta-ketoadipate pathway transcription regulator	Sinorhizobium medicae WSM419]
76	hypothetical protein	Yersinia ruckeri]
77	peptidyl-tRNA hydrolase	Methylobacterium sp. 4-46]
78	substrate-binding protein	Yersinia enterocolitica subsp. enterocolitica 8081]
79	hypothetical protein Daes_2420	Desulfovibrio aespoeensis Aspo-2]
80	ABC-type transporter, auxilary periplasmic component involved in toluene tolerance	[Ralstonia eutropha H16]
81	HypF2	Ralstonia eutropha H16]
82	ABC transporter HlyB/MsbA family protein	Maritimibacter alkaliphilus]
83	type VI secretion protein ImpA	Nitrococcus mobilis]
84	hypothetical protein CV_2817	Chromobacterium violaceum ATCC 12472]
85	carbamoyl phosphate synthase small subunit	Methylosinus trichosporium]
86	hypothetical protein	Candidatus Regiella insecticola]
87	hypothetical protein	Oceanicola batsensis]
88	phenylacetate--CoA ligase	Geobacter daltonii FRC-32]
89	seryl-tRNA synthetase	Nitratifractor salsuginis DSM 16511]
90	tRNA pseudouridine synthase D TruD	Anaeromyxobacter dehalogenans 2CP-1]
91	phosphoserine phosphatase SerB	Methylotenera versatilis 301]
92	Acyl-CoA synthetase (AMP-forming)/AMP-acid ligase II	Magnetospirillum magneticum AMB-1]
93	purine nucleoside phosphorylase	Sinorhizobium medicae WSM419]

MEV for PHA

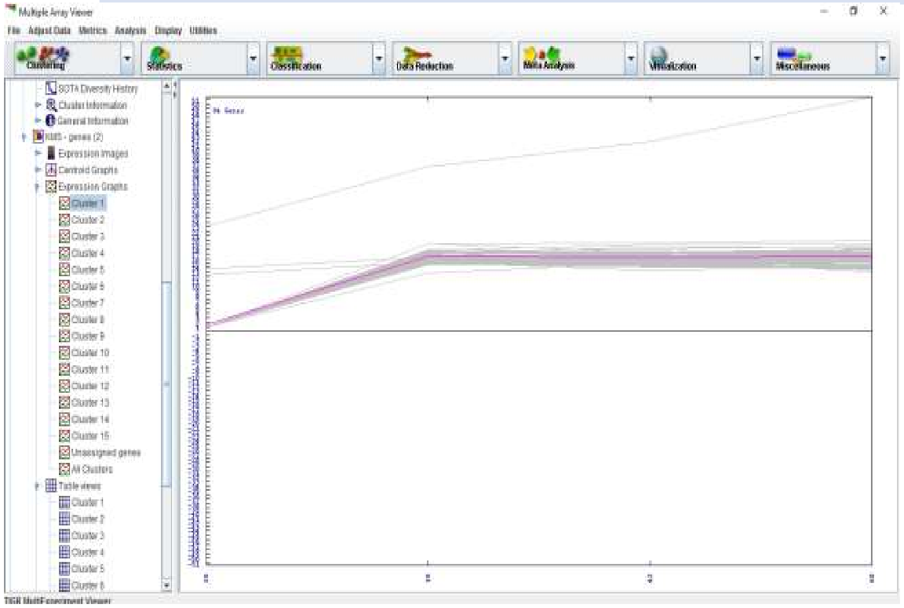

MEV for glucose

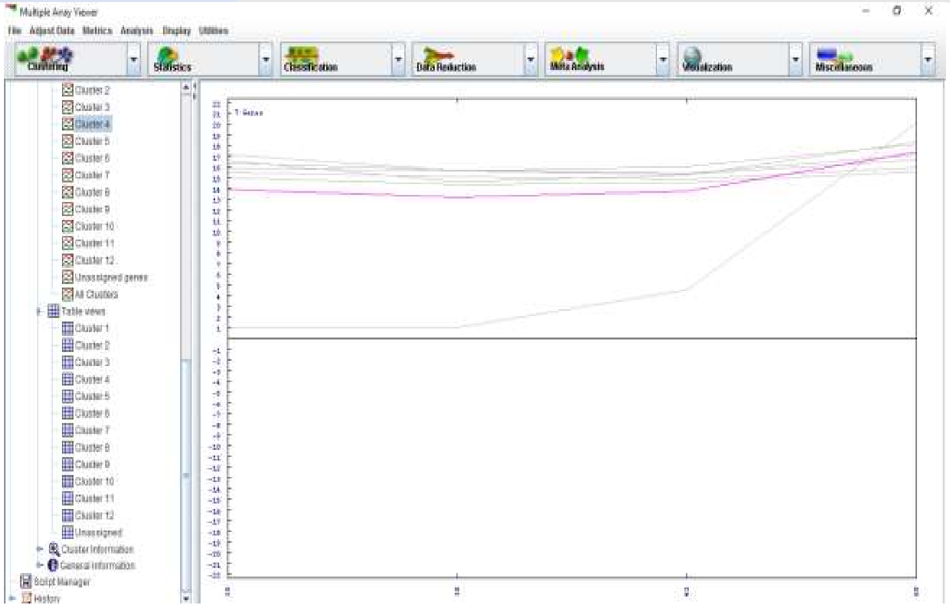

Supplement: Supplementary file 1 [file bioengineering-07-00154-s001.pdf]
